# Validation and Correction of PM_2.5_ Mass Concentrations from Consumer-Grade Personal Exposure and Outdoor Fixed Monitors: A Two-Year Colocation Study in Colorado

**DOI:** 10.1021/acsenvironau.6c00020

**Published:** 2026-08-04

**Authors:** Aniya Khalili Hollo, Dulce Gonzalez-Beltran, Allison Heckman, Sophie Castillo, Nicholas Clements, Esther Sullivan, Shivakant Mishra, Shelly L. Miller

**Affiliations:** † Department of Mechanical Engineering, 1877 University of Colorado Boulder, 427 UCB, 1111 Engineering Drive, Boulder, Colorado 80309, United States; ‡ Environmental Engineering Program, 122701 University of Colorado Boulder, 4001 Discovery Drive, Boulder, Colorado 80309, United States; § Department of Sociology, 12226 University of Colorado Denver, 1380 Lawrence Street, Suite 420, Denver, Colorado 80204, United States; ∥ Department of Computer Science, 122701 University of Colorado Boulder, 430 UCB, 1111 Engineering Drive, Boulder, Colorado 80309, United States

**Keywords:** fine particulate matter, low-cost sensor, urban air pollution, monitor performance, air quality monitoring, personal exposure, sensor evaluation

## Abstract

Consumer-grade (CG) air quality monitors with low-cost sensors that measure different pollutants and environmental factors are used to assess personal exposure and indoor and outdoor air quality in many communities. These devices empower citizens to actively participate in understanding their air quality and reducing their exposure to air pollution by accessing data in real time. As part of the Social Justice and Environmental Quality Denver project (SJEQ-D), we evaluated the performance of Atmotube Pro personal exposure monitors (ATMs) and stationary QuantAQ Modulair-PM outdoor monitors (QAQs) by colocating them with a GRIMM EDM180 (GRIMM) PM_2.5_ reference instrument four times for multiple weeks before their use within communities. The significance of this evaluation is that it is one of the first to colocate a large number of CG air quality monitors (50 ATMs and 5 QAQs) over two years during different seasons in the Mountain West region of the U.S. This study is intended as a large-scale, field-based performance evaluation to establish fitness-for-purpose use in community exposure studies, rather than as a sensor development, mechanistic, or regulatory equivalence analysis. The mean correlation coefficient and root mean squared error (RMSE) between the 1-h averaged GRIMM and raw ATM data were ρ = 0.770 and RMSE = 5.12 μg m^–3^; the corresponding values for the raw QAQ data were ρ = 0.720 and RMSE = 5.51 μg m^–3^. These correlation coefficients indicate that both ATM and QAQ monitors capture a substantial portion of the temporal variability in PM_2.5_ measured by the reference instrument. However, both monitors underestimated PM_2.5_ concentrations, particularly above 30.0 μg m^–3^. In addition, temporal drift was observed in the raw ATM and QAQ data sets, with individual monitors showing measurable changes in response across colocation time periods, indicating differences in long-term performance stability. The mean intramonitor correlation for the ATMs and QAQs was ρ = 0.980 and ρ = 0.780, respectively. When compared to each other, 5–11% of the ATMs were not working. A multilinear regression correction model incorporating relative humidity and dew point was applied to the ATM and QAQ colocation data. This correction increased the mean correlation coefficient by 6.5–8.3% and decreased RMSE by 42–50% relative to the reference instrument, while also reducing the temporal drift observed in the raw data. Overall, our colocation results show that ATMs and QAQs provide useful PM_2.5_ measurements for personal exposure and outdoor air monitoring, and that applying appropriate corrections improves agreement with reference instruments. In situations where reference data are unavailable, colocating CG monitors with one another can provide valuable insight into device performance and data reliability.

## Graphical abstract

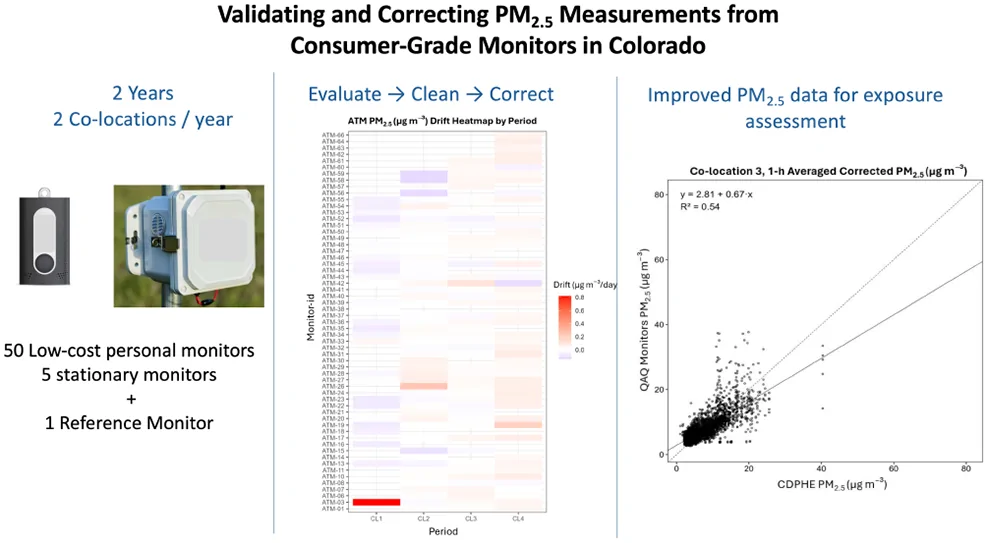

## Introduction

Urban communities are exposed to air pollution from various sources, including traffic, construction, and industrial facilities, which are linked to adverse health outcomes such as respiratory infections, cardiovascular diseases, and death.^1–7^ Airborne particles with diameters less than 2.5 μm (PM_2.5_) are of concern as PM_2.5_ exposure can lead to respiratory and cardiovascular inflammatory issues and increase the risk of dying prematurely.^8–14^ Studies have shown higher PM_2.5_-associated disease in neighborhoods with lower household incomes and a higher proportion of people of color.^15–17^ These disproportionately impacted (DI) communities are those with a historical backdrop of environmental injustice perpetuated by redlining and discriminatory laws against certain populations (e.g., Indigenous, immigrant, Hispanic, or Black). DI communities are characterized by a convergence of multiple factors that collectively influence health, the environment, and contribute to enduring disparities.^18^ Large health discrepancies and disproportionate air pollution exposure among communities of color and low-income communities within the U.S. have been reported.^19–21^

Consumer-grade monitors (CGMs) that incorporate low-cost sensors measuring air pollutant concentrations are being used to assess air pollution in urban areas,^22,23^ and are a promising and accessible tool for DI communities to use in measuring the air pollution in their local environments. Hyperlocalized deployments of large numbers of indoor and outdoor monitors have been made possible due to their low cost and the burgeoning consumer-grade air monitor manufacturing.^19,24,25^ In parallel with the increased use of CGMs in DI communities, smart technology is revolutionizing the way users assess their environment. Coupling CGMs with smartphones, the Internet of Things, and cloud computing now enables real-time air pollution measurement, awareness, and mitigation.^26–29^

The Social Justice and Environmental Quality Project in Denver, Colorado (SJEQ-D), was designed to assess the impact of planned community disruptions, specifically large construction projects, on personal PM_2.5_ exposure and outdoor concentrations in disproportionately impacted communities. A key strength of SJEQ-D is its use of CGMs to capture PM_2.5_ concentrations over extended periods and across multiple seasons in communities affected by diverse air pollution sources, including industry, traffic, and major construction activities.^30,31^

Because CGMs rely on low-cost sensors with variable accuracy and precision, appropriate validation is needed prior to deployment,^32^ particularly for long-term community studies. Prior studies indicate that CGMs often require region-specific validation and periodic correction, which can increase time and resource demands.^32^ Accordingly, before deploying monitors in SJEQ-D, we conducted a series of field colocation experiments with a regulatory reference instrument to evaluate monitor performance under local environmental conditions.

The objective of this study was to evaluate and apply established correction approaches to PM_2.5_ data collected by a large number of CGMs, with particular focus on their use in DI community exposure studies.^33^ This paper focuses on the performance evaluation of the two CGMs deployed in SJEQ-D under real-world, community-based deployment conditions. Results from the personal exposure assessment are presented elsewhere,^34^ and broader environmental and atmospheric analyses fall outside the scope of this paper.

Here, we emphasize a large-scale, multiseason colocation field study as a foundational methodological component of community-based environmental health research, rather than as an atmospheric chemistry or sensor development study. The novelty of this work lies in the deployment and colocation of a large number of CGMs (50 personal monitors and 5 outdoor monitors) over two years, enabling the evaluation of performance variability, identification of defective units, and characterization of monitor-specific drift under real-world conditions. This study does not aim to propose a new calibration methodology or establish regulatory equivalence. It documents how widely used consumer-grade PM_2.5_ monitors behave under sustained, real-world deployment with the goal of establishing fitness-for-purpose performance and informing practical deployment strategies for community exposure studies. A mechanistic investigation of sensor aging or component-level drift would require controlled laboratory experiments and is, therefore, beyond the scope of this field evaluation.

## Methods

The Atmotube Pro (Atmotech Inc., San Francisco, CA, ATM) was used in the SJEQ-D study. The Atmotube is a personal exposure monitor that uses a low-cost PM sensor, the Sensirion SPS30,^35^ and was introduced to the market in 2018.^36,37^ The ATM is designed to be carried around by the user, weighing 104 g, and measures PM_1_, PM_2.5_, and PM_10_ (using the Sensirion SPS30 sensor) mass concentrations, total volatile organic compounds (TVOCs, Sensirion SGPC3 sensor),^38^ temperature, and relative humidity (RH, Bosch BME280 sensor).^39^ Performance testing demonstrated that PM_2.5_ mass concentrations measured by the Sensirion SPS30 are moderately to strongly correlated with measurements made with research- and professional-grade instruments.^23,36,40–43^ Studies colocating ATMs in the field and in the lab demonstrated a strong correlation between ATMs and a range of particle monitoring instruments.^44–46^ For example, Shittu et al. (2025) showed that ATMs performed well against the reference-grade Fidas system.^45^ Pérez et al. (2024) conducted field tests of the ATM compared to the GRIMM 11-D as the comparison device, with the ATM demonstrating acceptable accuracy depending on particle size (R^2^ ranging from 0.47 to 0.93).^46^

The QuantAQ (QuantAQ, Inc., Somerville, MA, QAQ) was used in the SJEQ-D study to measure local outdoor air pollution. It is designed to be a stationary monitor and is installed on a stand or post outdoors. The QuantAQ uses two low-cost PM sensors, the Plantower PMS5003 ^47^ and the Alphasense OPC-N3,^48^ to measure the concentrations of PM_1_, PM_2.5_, and PM_10_. Studies showed that both sensors performed well compared to regulatory instruments.^36,41,49^ The QAQ performed well when colocated with research-grade instrumentation.^50^

Atmotube data were collected every minute and stored for up to 7 days in the ATM memory. ATM users access their data through the ATM smartphone app that provides real-time and cumulative data via graphs and an Air Quality Score (the AQS is based on a published algorithm^51^ combining TVOC and particulate concentrations and ranges from 0 (severely polluted air) to 100 (very clean)^52^). We downloaded the application programming interface (API) data as well as emailed the data directly from the sensor to our team’s email address.

To access the QAQ data, we downloaded the API data at a time interval of 1 min.^53^ The Colorado Department of Public Health and Environment (CDPHE) reference monitor used in this study is a GRIMM EDM180 reference monitor. The GRIMM is a regulatory FEM monitor for PM_2.5_ and is informative for PM_10_. At the colocation site used in this study, temperature, RH, wind speed, and direction were measured using R.M. Young meteorology equipment (models 41382 and 05305).^54–56^

During our main SJEQ-D, four community air sampling studies were conducted from 2021 to 2023. Before each cohort study, the ATMs and QAQs were colocated with CDPHE’s GRIMM reference monitor located at the Interstate 25 (I-25) Globeville CDPHE air pollution monitoring station (EPA AQS ID: 080310028)^57^ to validate the monitors’ performance and gather data for generating correction models for the ATM and QAQ particulate matter (PM_2.5_) data. The site is less than 50 m east of I-25 and less than 500 m from the I-70 and I-25 intersection (Latitude: 39.785622, Longitude: −104.988460). The colocation timeline and the number of monitors deployed in each colocation are shown in Table 1, which includes the number of working ATMs after removing the defective units, determined by intercomparison of measured concentrations.

**Table 1:** Colocation Timeline and the Number of Working Monitors

Colocation (CL)	Start-date-time MST^a^	End-date-time MST^a^	# QuantAQ MODULAIR-PM	# Atmotube PROs^b^
CL1 Fall	2021-11-15 00:00	2021-11-23 23:59	5	30 of 55 had ≥75% data
CL2 Spring	2022-04-20 00:00	2022-05-13 23:59	5	51
CL3 Summer	2022-08-16 00:00	2022-09-16 23:59	5	46
CL4 Winter	2023-01-10 00:00	2023-02-10 23:59	5	48

a MST is the Mountain Standard Time zone.

b In each CL, 55 ATM units were initially deployed.

Since ATMs are not waterproof, a structure was built to protect them from harsh weather, such as wind, rain, and snow, while allowing airflow through the structure (Figures S1 and S2, in the Supporting Information (SI)). The location of each monitor within the structure was kept consistent across all colocations; however, if the monitor was defective, it was replaced with a new monitor from the manufacturer. The QAQ monitors are waterproof, and the five QAQs were installed on a tripod and secured with zip ties (Figure S3).

During each colocation, our research team visited the site twice per week. During one of the visits, the team checked that the ATMs were all plugged in and operating, and that the protective structure was not damaged. During the second visit, the research team manually exported data from each ATM. During CL1, we relied on the syncing option of the Atmotube monitors, which meant sending data that was stored on the ATMs to the Atmotube cloud for storage (SI Figure S4 shows instructions explaining how to synchronize the ATM data). Due to data loss from occasional Bluetooth disconnections, data were exported manually during subsequent colocations, and no data were lost during colocations two through four. 30 out of 55 ATMs deployed in CL1 synchronized sufficient data for comparison with the reference monitor (≥75% completeness), while 25 ATMs did not.

## Data Processing and Analysis

Figure 1 describes the data processing method. The first step was to determine whether any of the ATMs and QAQs were defective by calculating the Pearson correlation matrix, comparing all the ATM and QAQ monitors with each other and with the reference GRIMM instrument. If the correlation coefficient (ρ) for a monitor was consistently <0.30, we then investigated the time series data for PM_2.5_, temperature, and RH to confirm whether the monitor was defective. For example, when we observed sudden extreme values (e.g., constant values for several days) in a PM_2.5_ time series, which were different from other ATM time series, we would flag this as a defective monitor and exclude the monitor’s data from our metadata and subsequent analyses. When similar patterns were also noticed in RH and temperature time series, these data were also excluded. In some cases, the ATM indicated a hardware defect (white blinking LED) or powered off and was replaced.

**Figure 1:**
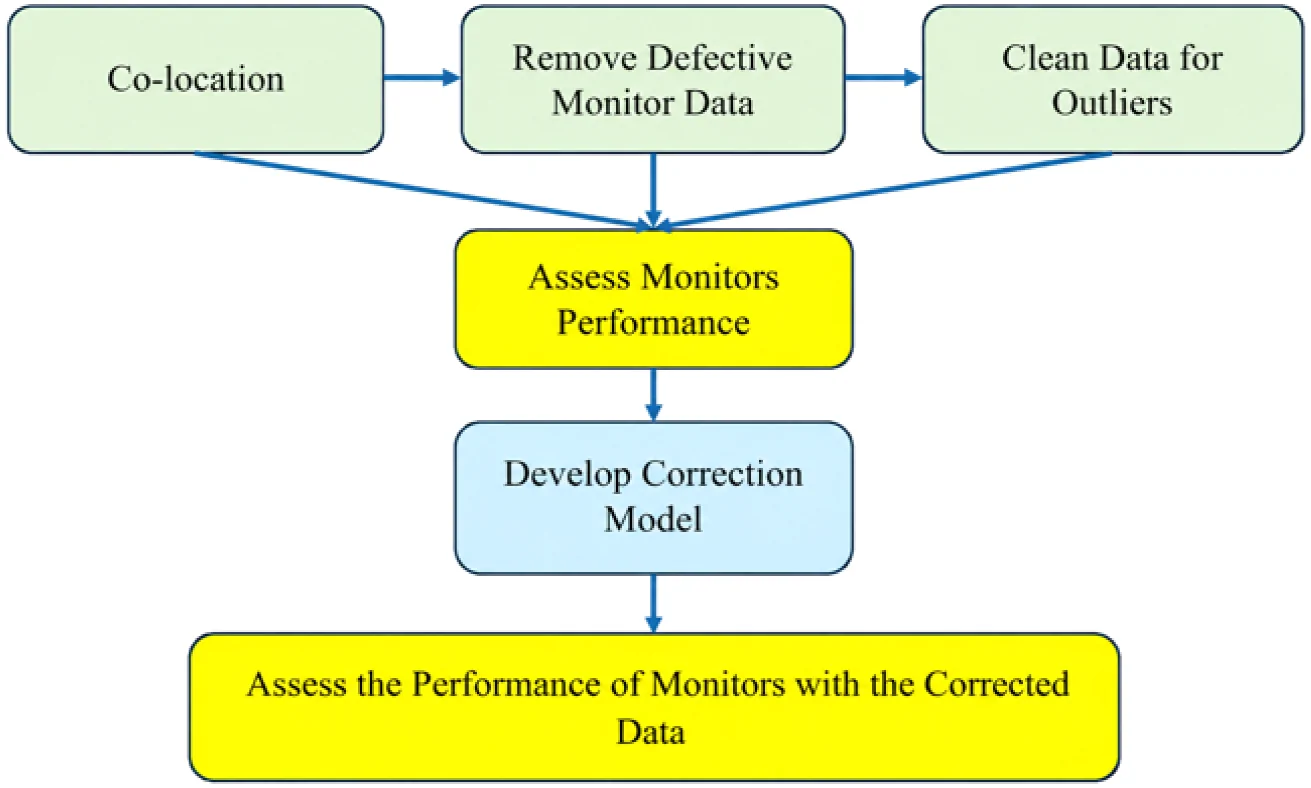
Diagram of the data processing method used in each colocation.

If a monitor was identified as defective during the colocation phase, it was replaced with a new unit from the manufacturer prior to community deployment. This ensured that only properly functioning monitors were distributed for use in the community cohort studies and minimized the impact of hardware failures on data completeness and quality.

After identifying and removing defective monitors, the raw data were reviewed for outliers, which were removed. PM_2.5_ measurements above 500 μg m^–3^ were replaced with not available (*NA*) because they exceeded the CGM measurement range; however, no data points were above this limit. The recommended operating conditions for the Sensirion sensor in the ATM are reported to be best between 20% and 80% RH;^58^ the recommended conditions for the QAQs are between 5 and 95% RH.^59^ Most consumer-grade PM monitors show that RH starts to significantly impact the PM_2.5_ values when RH exceeds 80–85% due to hygroscopic growth of particles.^60^ Because the objective of our study was to assess PM_2.5_ concentrations in a community exposure study as accurately as possible, we decided to remove data that were outside of these optimal RH operating conditions. We considered the proportion of data falling below one-half of the lower recommended operating limit (RH < 10%) to capture the influence of our study location’s dry ambient conditions on data (median RH values ranged from 24.2 to 33.1% for ATMs, and median RH from 26.0 to 49.3% for QAQs). All PM_2.5_ data in which the RH was below 10% and above 80% were replaced with NA; the RH data exclusions were 0 to 18% of the overall data set (SI Table S1). Across the ATMs, RH < 10% occurred in 7–18% of observations and RH > 80% in 0.1–4%. For the QAQs, 0.7–11% of the data set fell below 10% RH and 0–4% exceeded 80% RH. All PM_2.5_ measurements where the temperature was also measured either below −39.5 or above 85.5 °C were replaced with NA, consistent with the operating range of the temperature sensors in our CGMs.^58–60^ However, some monitors exhibited elevated sensor temperatures during outdoor deployment, likely due to enclosure heating from solar radiation despite the use of protective shelters or, in some cases, potential monitor malfunction. Monitors displaying persistent abnormal PM_2.5_, temperature, or RH behavior were identified during quality-control screening and excluded from subsequent analyses. We did not conduct a formal sensitivity analysis for RH < 10% conditions; therefore, results should be interpreted with this limitation in mind for dry environments.

Sensor drift for the ATM and QAQ PM_2.5_ data was evaluated by comparing each monitor’s hourly measurements with colocated reference data across all four colocation periods. For each monitor, we calculated the hourly bias (monitor – reference) and the mean of monitor and reference concentrations ((monitor + reference)/2) and examined systematic error using Bland–Altman plots.^61^ Drift over time was also quantified by fitting a linear regression of bias against time within each colocation period, where the slope represents the drift rate (μg m^–3^ day^–1^).

Hourly bias was modeled using multiple linear regression: Bias = α_0_ + α_1_ × RH + α_2_ × T + α_3_ × time. In this model, bias is the total monitor-reference difference in μg m^–3^, while the regression coefficients quantify the components of that bias associated with relative humidity, temperature, and long-term temporal drift. α_0_ is the intercept, α_1_ through α_3_ are regression coefficients, RH is the relative humidity (%), T is the temperature (°C), and time is expressed in days. The coefficient for time (α_3_, in μg m^–3^ day^–1^) quantifies the long-term change in monitor bias after adjusting for the effects of temperature and RH. The statistical significance of the regression coefficients was evaluated using two-sided *t*-tests.

In this study, both the Pearson correlation coefficient (ρ) and the coefficient of determination (R^2^) were used to evaluate monitor performance. The Pearson correlation coefficient quantified the strength and direction of the linear association between the consumer-grade monitor data and the reference instrument data, independent of bias. R^2^ was derived from applying a linear regression comparing ATM and QAQ PM_2.5_ data that were averaged on an hourly basis with the data collected by the GRIMM reference instrument;^62,63^ it represents the proportion of variance in the reference measurements explained by the CGMs, incorporating both correlation and model fit. In addition to R^2^, regression slopes, intercepts, root-mean-square error (RMSE), and normalized root-mean-square error (NRMSE) were calculated for performance evaluation. These standard performance metrics were calculated following U.S. EPA guidance (see SI Equations S1–S5).^64^ Note that the performance metrics were derived from linear regression models used for performance evaluation, while the multilinear regression model described below was used separately for correction of CGM data.

A correction model was derived for the CGMs to correct the PM_2.5_ data for possible ambient environmental impacts, using multilinear regression (MLR); this approach has been used previously.^62,65,66^ We explored different combinations of meteorological parameters and selected the model with the highest R^2^ and lowest standard error (SE) compared to other combinations of data. Parameters in the correction model are β_0_, the intercept; β_1_, the monitor PM_2.5_ coefficient; β_2_, the RH coefficient; and β_3_, the T_dew_ coefficient. T_dew_ for each monitor was calculated using the RStudio Weather package.^67,68^

1 $$PM_{Corrected}=\beta_{0}+\beta_{1}\times \left(\mathrm{monitor}PM_{2.5}\right)+\beta_{2}\times RH+\beta_{3}\times T_{dew}$$

We explored two methods to develop the correction models for each colocation: (1) an individual model by estimating parameter values for each CGM independently, and (2) an overall model by averaging the parameter values across all the individual models. We conducted a sensitivity analysis comparing results when the data for each CGM were corrected using the individual models, compared to when the data were corrected using the overall model. The results were similar, and we selected the overall model approach for ease of use in data processing and analyses. The Pearson pairwise correlation was calculated by comparing the corrected PM_2.5_ of each CGM with the GRIMM reference instrument to assess monitor performance improvement with the correction model.

## Results and Discussion

Most ATMs and all QAQs were working during our colocation studies, as determined by comparing Pearson correlation matrices, before and after removing the defective monitors’ data (see SI Figure S5, for example). The number of ATM monitors that were observed not to be functioning properly, and therefore removed from each colocation data set ranged from zero to eight.

Figure 2 shows the 1-h averaged PM_2.5_ concentration time series for Colocation 3 for data from an (a) ATM and a (b) QAQ, compared with the GRIMM reference instrument. The trends are captured by the ATM and QAQ; however, they measured lower concentrations compared to the GRIMM. The air pollution episode that occurred in early September, when the PM_2.5_ rose to a peak of 20 μg m^–3^, was measured more accurately compared to other dates.

**Figure 2:**
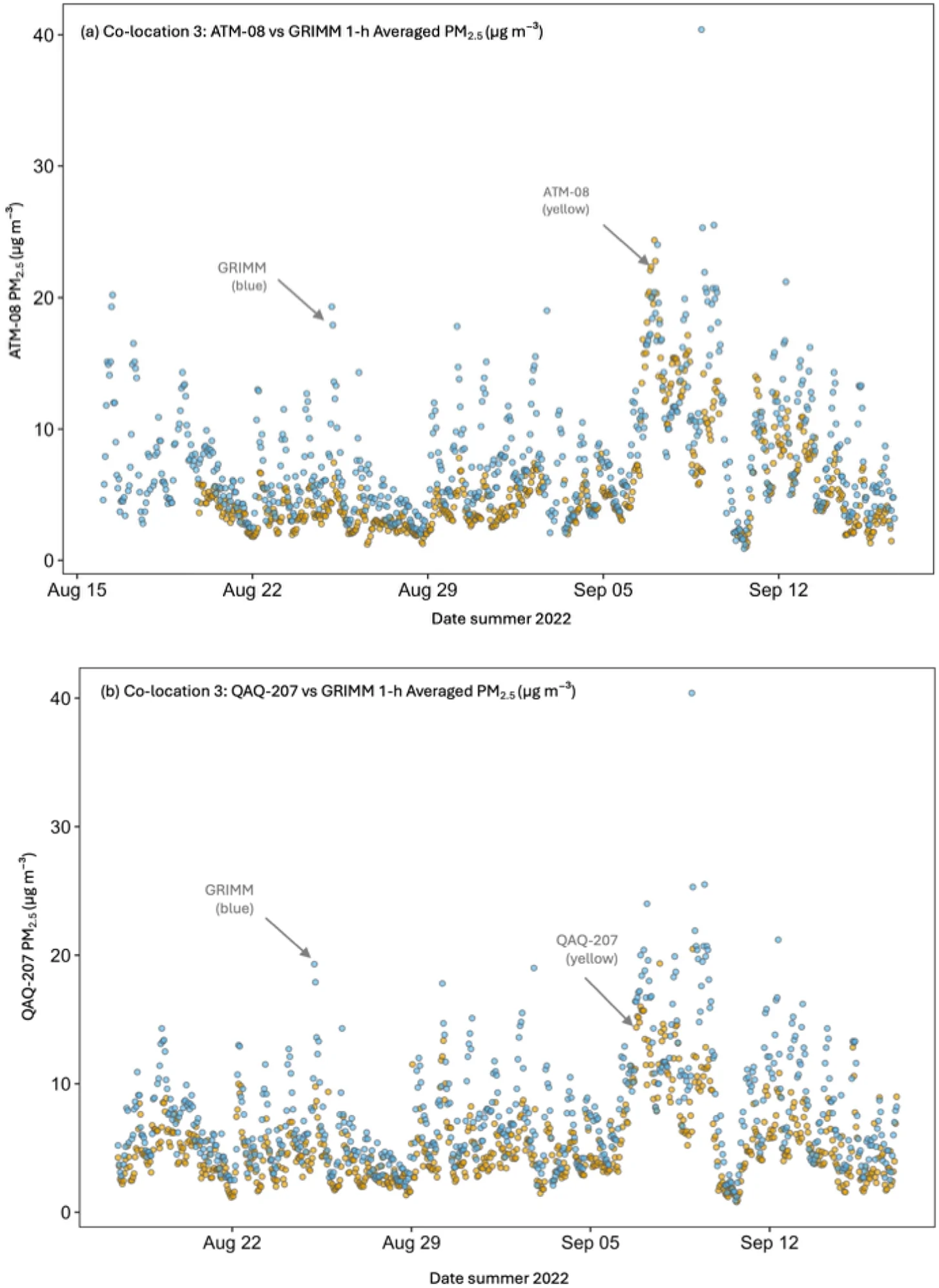
One-hour averaged PM_2.5_ concentrations (μg m^–3^) over time for Colocation 3 for data from an (a) ATM (ATM-08) and a (b) QAQ (QAQ-207), compared with the GRIMM reference instrument. Blue dots indicate the GRIMM data, and yellow dots are ATM or QAQ data.

The ATM, QAQ, and GRIMM monitors showed high intra- and intermonitor correlation. Mean ± SD intramonitor correlations for the ATMs and QAQs were ρ = 0.982 ± 0.029 and ρ = 0.782 ± 0.134, respectively. The mean correlation and root-mean-square error (RMSE) between the 1-h-averaged GRIMM and ATM data were ρ = 0.770, RMSE = 5.12 μg m^–3^. The mean correlation and RMSE between the 1-h-averaged GRIMM and QAQ data were ρ = 0.721, RMSE = 5.51 μg m^–3^.

Figure S6 in the SI presents the pairwise Pearson correlation between each of the ATM monitors and the other ATMs, as well as the GRIMM instrument at each colocation, after the removal of defective ATM data and outliers. The pairwise Pearson correlation results for the QAQ monitors are shown in SI Figure S7.

Meteorological data collected by CDPHE at the colocation site were compared to the temperature and relative humidity data from the ATM and QAQ monitors (see SI Tables S2, S3, S4, and SI Figures S10 and S11). These data compared well, with T_dew_ having an R^2^ value between 0.710 and 0.820, and RH having an R^2^ value between 0.820 and 0.920. The temperature measured by the ATMs had an R^2^ ∼ 0.840, and the QAQs R^2^ ∼ 0.940, for 1-h means. Similarly, the RH measured by the ATMs had an R^2^ ∼ 0.820, and the QAQs had an R^2^ ∼ 0.950, for 1-h means. Zheng et al. (2022) evaluated low-cost air quality monitors in a laboratory setting to assess indoor air quality at Dutch daycare centers.^37^ In their study, ATM monitors’ PM_2.5_ measurements were more accurate in a cold and dry environment (R^2^ = 1.00) than in a warm and humid environment (R^2^ = 0.400). In our study, the ATM monitors had the highest R^2^ = 0.730 in colocation 1, the fall colocation with a dry and cold environment, agreeing with the Zheng et al.’s (2022) study. In our study, Colocation 4, the winter colocation with a more humid but cold environment, had the second-highest R^2^ = 0.690. The performance of the ATMs in CL2 and CL3 was not as good, and both occurred with a higher temperature and RH < 35.0%.

The sensor drift analysis indicated that drift was monitor-, concentration- and deployment-period-dependent, with larger drift observed at higher concentrations and after approximately 1.5 years of use. Figure 3 is a Bland–Altman plot for the ATMs, showing increasing negative bias at higher PM_2.5_ concentrations; similar drift behavior was seen for the QAQs (Figure S8). The drift rate heatmap for ATMs (Figure 4) and QAQs (Figure S9) shows that during colocations 1 through 3, the drift over time was variable but minimal. However, by CL4 after almost 1.5 years of use, drift was consistently larger compared to earlier periods. Similar deployment-age-dependent deterioration has been observed during long-term field evaluations of low-cost PM sensors.^69^

**Figure 3:**
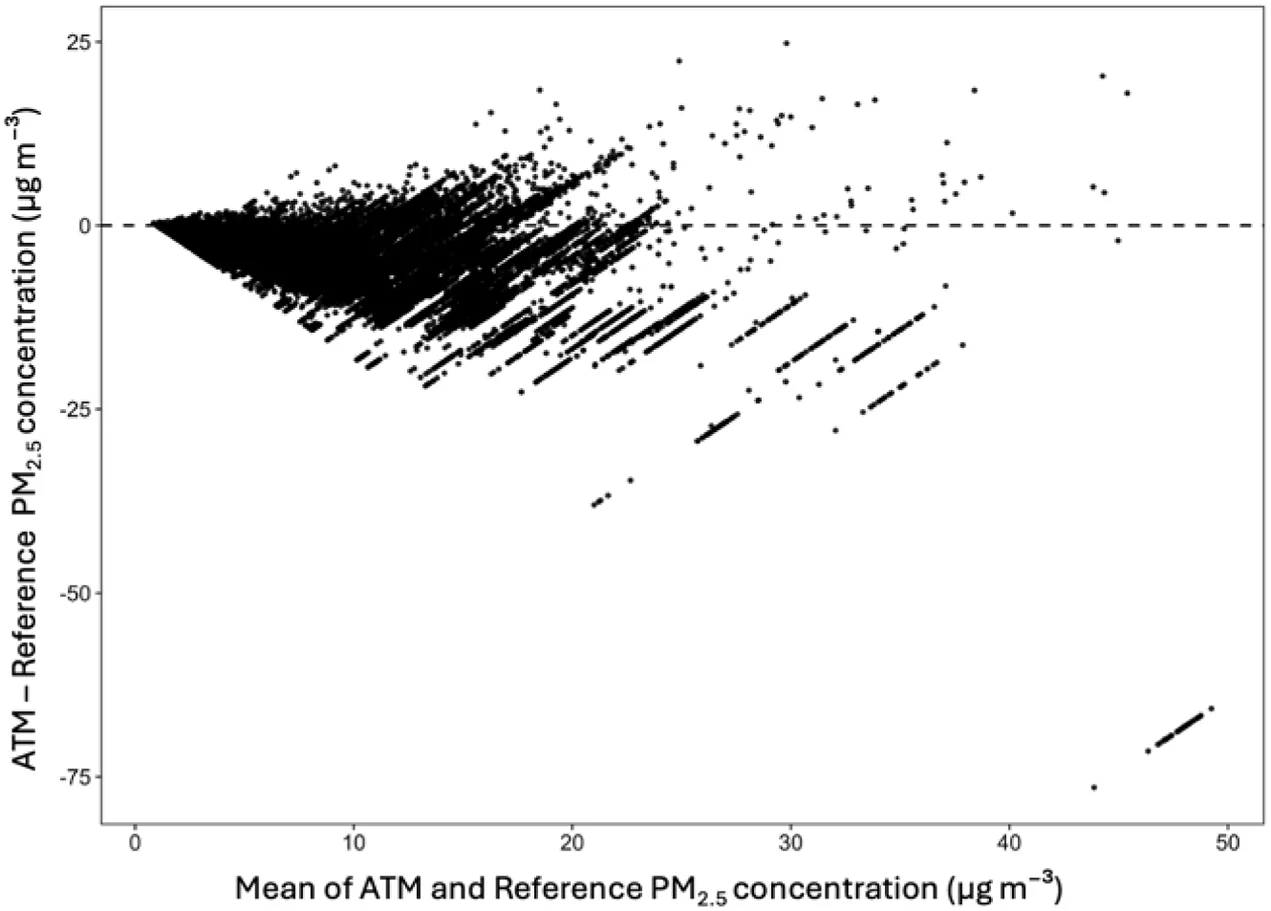
Bland–Altman analysis plots of the difference between ATM monitor and GRIMM reference PM_2.5_ concentrations (ATM monitor – reference) in units of μg m**^–3^** against the mean of ATM monitor and reference concentrations (ATM monitor + reference/2) in units of μg m**^–3^**. This plot allows visualization of bias and concentration-dependent error. The diagonal banding pattern arises from the discrete concentration values reported by the ATMs and the mathematical relationship between the Bland–Altman axes; it does not reflect differences in measurement timing.

**Figure 4:**
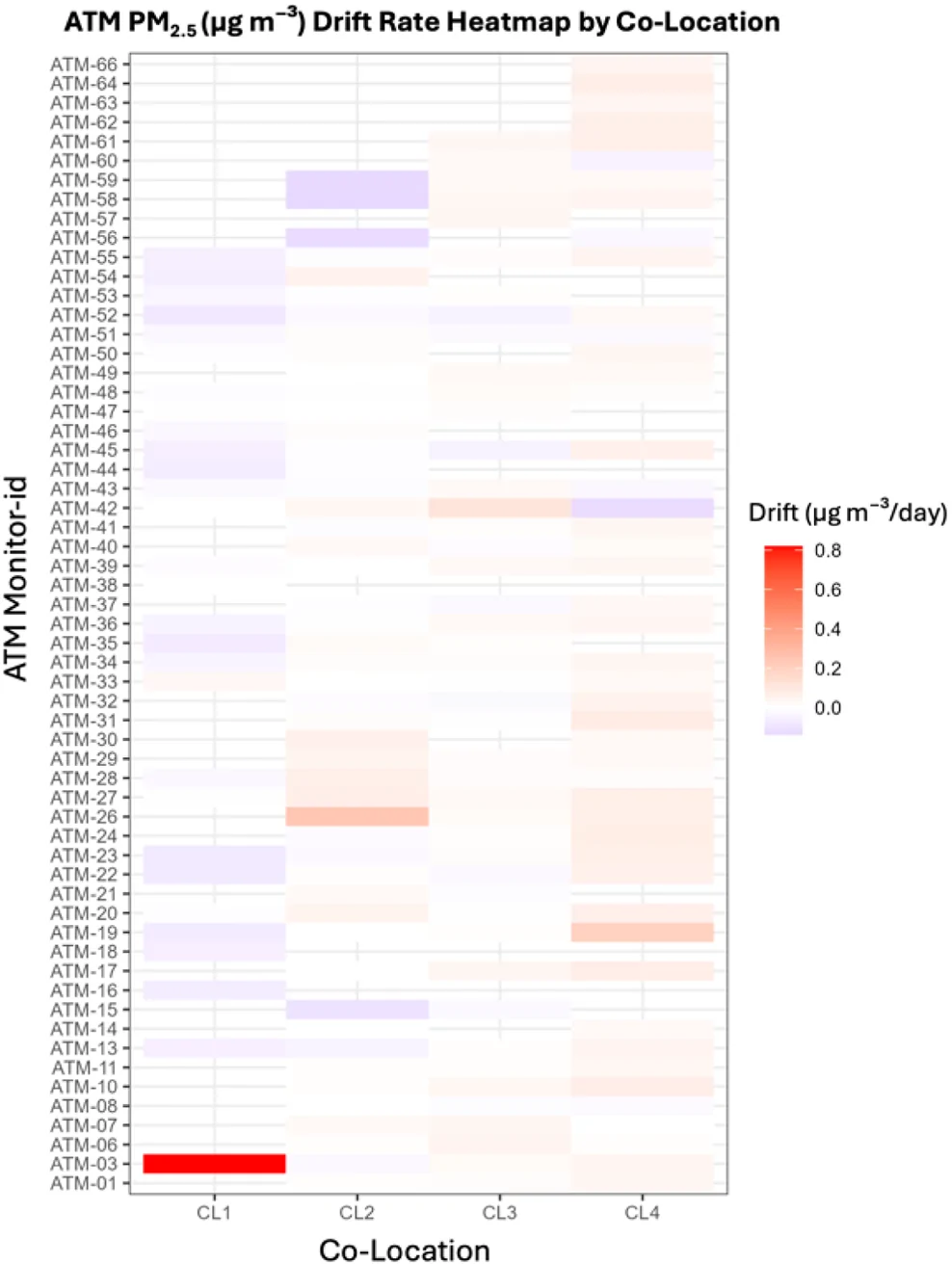
Heatmap of drift rate (μg m^–3^/day) for individual ATM PM_2.5_ concentrations for each colocation. Drift rates are the slope of a linear regression fit of bias against time within each colocation period.

Notably, the ATMs had higher drift rates compared to the QAQs, and a subset of ATM units exhibited substantial positive drift rates on the order of approximately 0.5–0.8 μg m^–3^/day during specific periods, while others remained largely stable. QAQ drift rates were generally smaller, ranging from −0.1 to 0.25 μg m^–3^/day indicating comparatively greater temporal stability of the QAQs under similar deployment conditions.

To better understand the source of drift, a multilinear regression model was applied to the data. Table 2 summarizes the regression coefficients for the intercept (α_0_), RH (α_1_), temperature (α_2_), and temporal drift (α_3_) for both ATM and QAQ monitor groups. Results show that RH and temperature coefficients were generally small for both monitor types, indicating that meteorological conditions contributed only modestly to variability in monitor bias. ATM monitors exhibited slightly positive mean RH and temperature coefficients, whereas QAQ monitors showed predominantly negative coefficients, suggesting differences in meteorological sensitivity between monitor groups. The statistical significance of regression coefficients varied among monitors. Relative humidity and temperature were statistically significant predictors of bias (*p* < 0.05) for 66% and 75% of ATM monitors, respectively, and for 100% and 60% of QAQ monitors, respectively. RH and temperature were retained in the model to account for environmental influences on sensor response and to isolate the temporal drift component.

**Table 2:** Summary of Regression Coefficients for ATMs and QAQs, Estimated from the Multilinear Regression of Bias on Relative Humidity, Temperature, and Time

Intercept (α_0_)	ATMs	QAQs
Mean	–1.29 × 10^2^	8.74 × 10
Median	3.20 × 10	8.58 × 10
Range	–1.86 × 10^3^ to 1.84 × 10^2^	–7.73 to 1.48 × 10^2^

RH (*α*_1_) (μg m^–3^ %RH^–1^)	ATMs	QAQs
Mean	1.79 × 10^–2^	–3.60 × 10^–2^
Median	1.93 × 10^–2^	–3.48 × 10^–2^
Range	–4.71 × 10^–2^ to 6.72 × 10^–2^	–4.70 × 10^–1^ to –2.02 × 10^–2^

Temperature (α_2_) (μg m^–3^ C^–1^)	ATMs	QAQs
Mean	2.03 × 10^–2^	–4.65 × 10^–3^
Median	2.90 × 10^–2^	–6.12 × 10^–3^
Range	–9.29 × 10^–2^ to 1.05 × 10^–1^	–2.84 × 10^–2^ to 1.99 × 10^–2^

Environmental drift^a^ (time) (α_3_) (μg m^–3^/day)	ATMs	QAQs
Mean	5.43 × 10^–7^	1.61 × 10^–7^
Median	5.32 × 10^–7^	2.54 × 10^–7^
Range	–4.41 × 10^–7^ to 2.90 × 10^–6^	–4.72 × 10^–7^ to 5.83 × 10^–7^

Temporal drift coefficients (α_3_) were also small for both CGMs, with mean drift rates of 5.43 × 10^–7^ (μg m^–3^ day^–1^) for ATMs and 1.61 × 10^–7^ (μg m^–3^ day^–1^) for QAQs. The time coefficient was statistically significant for 63% of ATMs and 80% of QAQs, indicating measurable temporal drift for many, but not all, monitors. Median drift rates were similarly low, and the observed ranges included both positive and negative values, indicating variability in drift direction among individual monitors. These small drift coefficients represent residual long-term changes in monitor bias after accounting for RH and temperature effects rather than total sensor bias. Because long-term temporal drift was not evaluated in prior SCAQMD assessments, these results provide additional insight into CGM performance across multiple colocation deployments.^44^

The observation that drift is specific to each monitor and deployment does not imply limited utility for long-term studies; rather, it indicates that drift is manageable through periodic screening, at least for the first 1–1.5 years. High intermonitor correlations persisted despite drift, indicating that these monitors are suitable for assessing relative exposures and temporal trends, which are key objectives in community-based and environmental justice studies. Drift rates were small after accounting for temperature and relative humidity effects, suggesting that periodic intercomparison and replacement, rather than global recalibration with every use, is a practical strategy for community deployments under 1.5 years long. Similar concentration-, deployment-age-, and monitor-specific drift behaviors have been reported in other long-term field studies of low-cost PM sensors, where drift emerges after extended use, meteorological variables explain a small portion of bias, and strong intersensor correlations are maintained.^66,69,70^

Identifying the mechanistic causes of monitor-specific drift—such as optical fouling, aerosol composition effects, component aging, or manufacturing variability—would require controlled aerosol challenges, component diagnostics, or laboratory aging studies. Such analyses cannot be reliably inferred from field colocation data alone. The purpose of this work is therefore not to attribute drift to specific physical mechanisms, but to quantify its magnitude and operational implications under real-world deployment conditions.

Figure 5 shows scatterplots of 1-h averaged PM_2.5_ measurements comparing the ATMs with the GRIMM reference instrument. The ATM monitors consistently underestimated PM_2.5_, particularly above 30 μg m^–3^. We also observed a larger bias at higher concentrations (Figure 3). This deviation in concentration-dependent underestimation is consistent with known limitations of low-cost optical particle sensors.^71–73^ At elevated particle concentrations, nonlinear sensor response can occur due to coincidence errors, in which multiple particles simultaneously traverse the sensing volume and are detected as a single event, leading to undercounting of particle mass.^71^ Additionally, changes in particle size distribution and chemical composition under higher pollution or dust-influenced conditions can alter light-scattering properties, leading to systematic deviations from reference measurements that rely on gravimetric or beta-attenuation principles.^72,73^

**Figure 5:**
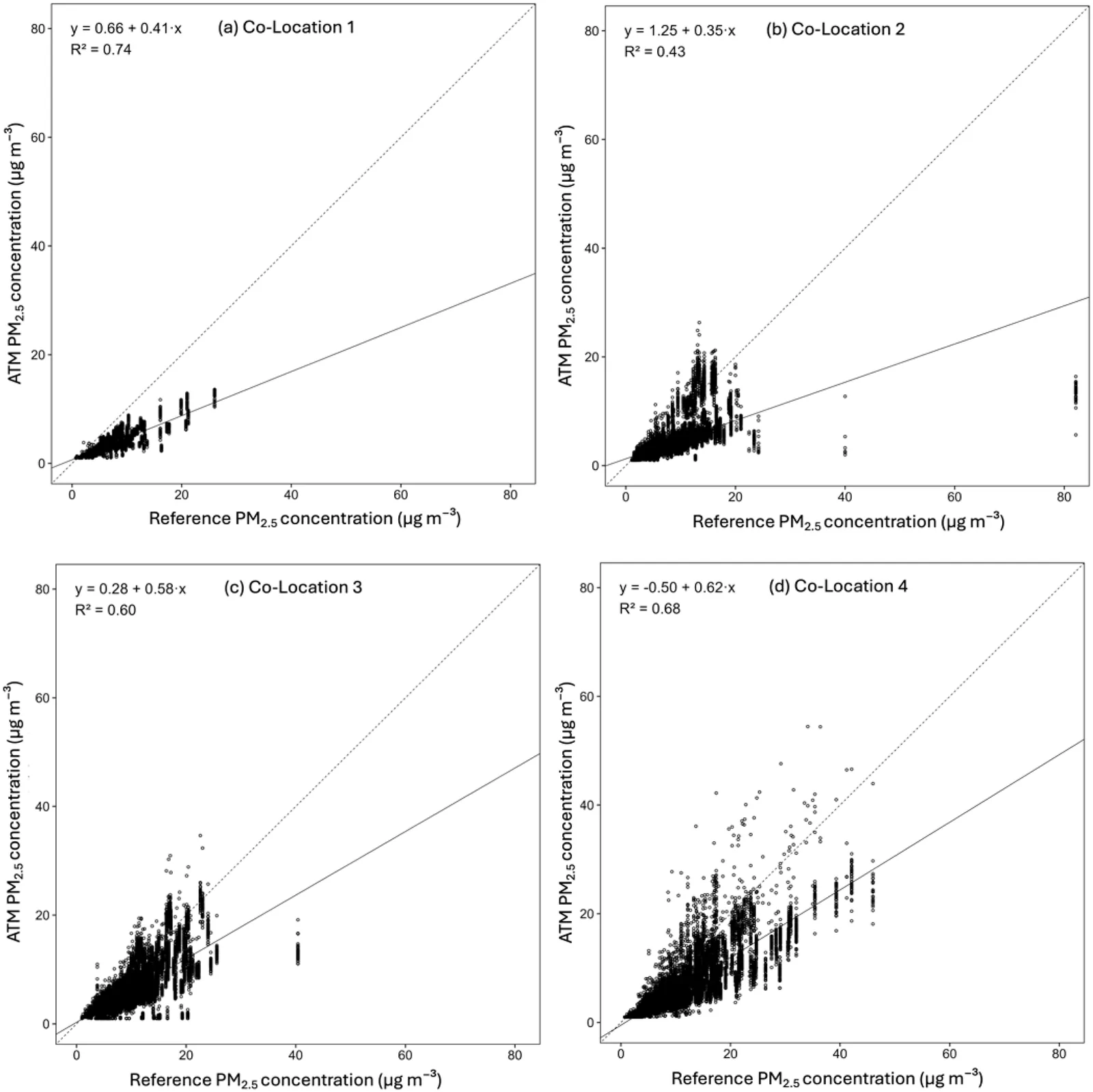
Scatterplots of the PM_2.5_ data from the ATM monitors (*y*-axis) versus the GRIMM reference instrument (*x*-axis) for colocations (a) 1, (b) 2, (c) 3, and (d) 4. The one-to-one line is shown on the graphs as dashed, and the linear regression model equation and R^2^ are shown on the plots.

Summary statistics for all four colocations are in SI Tables S5–S7. Colocation 4 had the highest mean PM_2.5_ levels across all monitors (6.59–11.4 μg m^–3^). Box and whisker plots for hourly PM_2.5_ data from the ATMs, QAQs, and GRIMM are in SI Figure S12. Figure 6 displays scatterplots comparing QAQ monitors with the GRIMM, which also underestimated PM_2.5_, and this underestimation becomes more pronounced at higher concentrations.

**Figure 6:**
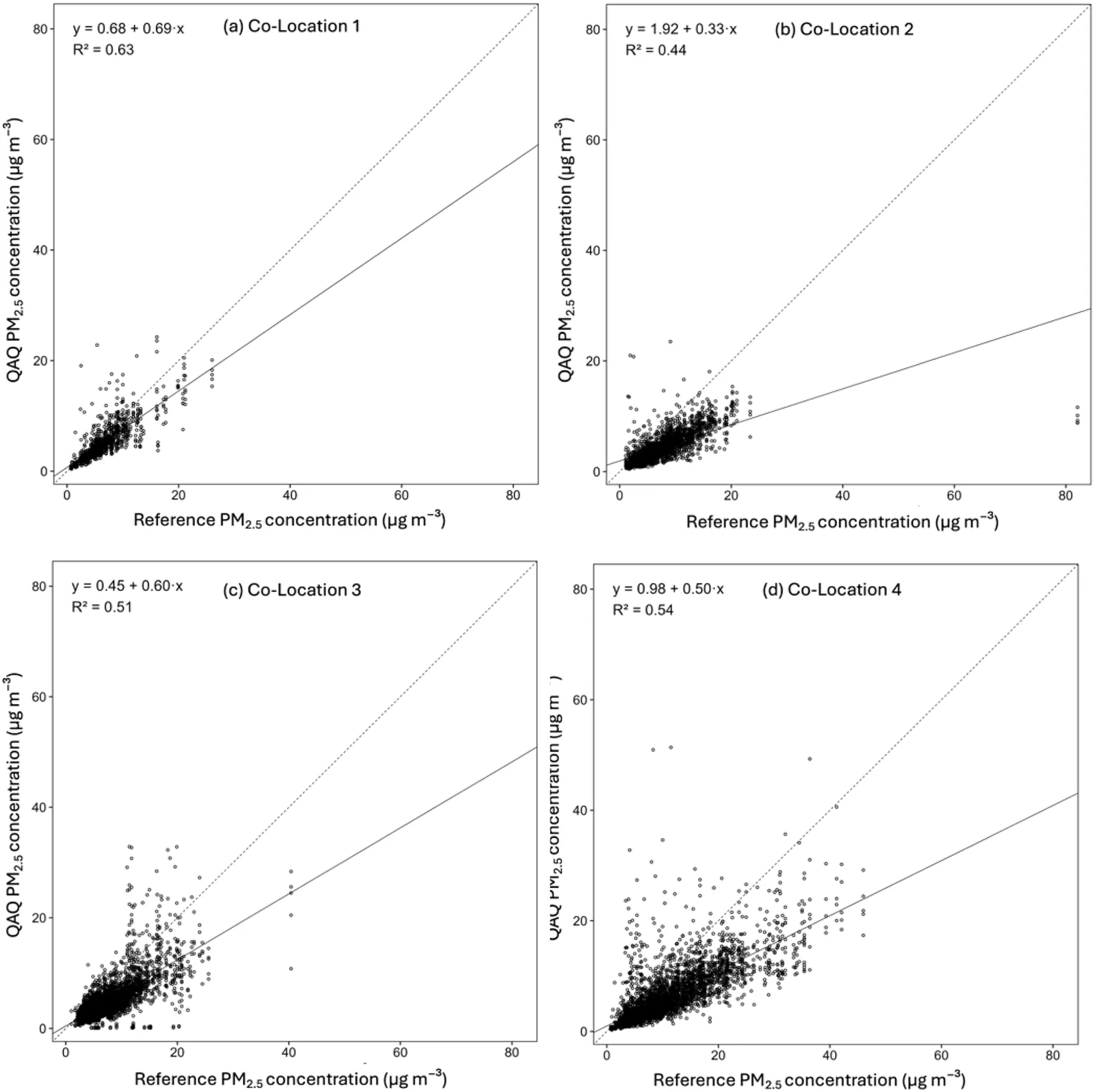
Scatterplots of the PM_2.5_ data from the QuantAQ monitors (*y*-axis) versus the GRIMM reference instrument (*x*-axis) for colocations (a) 1, (b) 2, (c) 3, and (d) 4. The one-to-one line is shown on the graphs as dashed, and the linear regression model equation and R^2^ are shown on the plots.

Table 3 displays the mean accuracy performance metrics for the comparison between the ATM and QAQ data and the GRIMM reference data. The values that meet the recommended accuracy standards are highlighted in bold. All colocations achieved the recommended performance metrics for y-intercepts and RMSE, but none met the NRMSE value. Detailed performance model results for each ATM/QAQ monitor versus the reference instrument in each colocation are provided in SI Tables S8–S15.

**Table 3:** Mean Performance Metrics for Linear Regression Model of ATM and QAQ Data with GRIMM Reference Data during Colocations at the CDPHE I-25 Globeville Site^a^

	Atmotube					QuantAQ				
Colocation	R^2^	Regression slope	y-Intercept (μg m^–3^)	RMSE (μg m^–3^)	NRMSE (%)	R^2^	Regression slope	y-Intercept (μg m^–3^)	RMSE (μg m^–3^)	NRMSE (%)
CL1	**0.730**	0.410	**0.620**	**4.38**	65.7	0.660	**0.690**	**0.660**	**2.91**	43.8
CL2	0.460	0.380	**1.09**	**5.40**	72.1	0.510	0.350	**1.780**	**5.14**	68.7
CL3	0.570	0.590	**0.500**	**4.06**	52.0	0.590	0.610	**0.400**	**4.14**	52.9
CL4	0.690	**0.740**	**–0.980**	**5.89**	51.8	0.630	0.500	**0.970**	**6.98**	61.3
U.S. EPA Recommendations^64^										
	>0.700	0.650–1.35	–5.00 to +5.00	≤7.00	≤30.0%	>0.700	0.650–1.35	–5.00 to +5.00	≤7.00	≤30.0%

a Bold indicates that performance metrics met U.S. recommendations.

Per Zimmerman et al., we evaluated different multilinear regression correction models using various data combinations.^62^ A final model, incorporating the GRIMM PM_2.5_, RH, and T_dew_ data (see eq 1), that demonstrated the highest performance was selected and applied to correct the ATM and QuantAQ data (see SI Tables S16 and S17 for an example). We assessed whether we should adjust each CGM with its specific MLR model or apply the average MLR model parameters. The differences between these two approaches were minimal (see SI Tables S18-S20 for QAQs and S21–S23 for ATMs); therefore, we used the average parameters for this study and subsequent SJEQ-Denver data analysis.^34^ Table 4 shows the parameters for the final multilinear regression correction model in each colocation (see eq 1).

**Table 4:** Multilinear Regression Correction Model Parameters for the ATMs and QAQs

	ATM Parameters				QAQ Parameters			
Colocation	Intercept β_0_	PM_2.5_ β_1_	RH β_2_	T_dew_ β_3_	Intercept β_0_	PM_2.5_ β_1_	RH β_2_	T_dew_ β_3_
CL1	6.89	2.11	–0.183	0.240	2.30	0.982	–0.029	–0.022
CL2	6.51	1.35	–0.104	0.193	2.21	1.39	–0.013	0.153
CL3	0.876	1.17	0.009	0.236	0.859	1.11	0.061	–0.082
CL4	7.90	1.36	–0.111	0.118	6.76	1.25	0.005	0.381

We applied the MLR correction model to our data and reassessed the U.S. EPA performance metrics by linear regression to evaluate any improvements in the comparison between the ATM/QAQ and the GRIMM reference data. Table 5 summarizes the performance metrics after applying the correction model. The correction model improved the R^2^ and slope values. For ATM monitors, the R^2^ values for the corrected data ranged between 0.53 and 0.77; however, the R^2^ values for CL2 and CL3 remained below the recommended value after the correction. Similarly, while the correction model improved the R^2^ for the QAQ monitors, ranging between 0.48 and 0.67, no values met the recommended value. All of the slope values met the recommended range after the correction and increased to 0.97–0.99.

**Table 5:** Mean Performance Metrics for Linear Regression Model between ATM and QAQ Data with GRIMM Reference Data after Applying the Correction Model^a^

	Atmotube					QuantAQ				
Colocation	R^2^	Regression slope	y-Intercept (μg m^–3^)	RMSE (μg m^–3^)^3^	NRMSE (%)	R^2^	Regression slope	y-Intercept (μg m^–3^)	RMSE (μg m^–3^)^3^	NRMSE (%)
CL1	**0.767**	**0.978**	**0.145**	**2.10**	**29.8**	0.671	**0.989**	**0.064**	**2.34**	33.9
CL2	0.525	**0.987**	**0.119**	**3.64**	48.9	0.484	**0.974**	**0.208**	**3.85**	50.6
CL3	0.648	**0.995**	**0.056**	**2.77**	34.4	0.640	**0.992**	**0.051**	**2.72**	34.5
CL4	**0.754**	**0.998**	**0.062**	**3.53**	32.0	0.664	**0.959**	**0.421**	**4.33**	38.2
U.S. EPA Recommendations^64^										
	>0.700	0.650–1.35	–5.00 to +5.00	≤7.00	≤30.0%	>0.700	0.650–1.35	–5.00 to +5.00	≤7.00	≤30.0%

a Bold indicates that performance metrics met U.S. recommendations.

One of the first field evaluations of ATM monitors was published in 2020 by the SCAQMD.^44^ These results showed a high correlation between the PM_2.5_ data compared with an FEM GRIMM (0.790 < R^2^ < 0.900, hourly averaged mean). Our results showed lower R^2^ for all colocations (0.525 < R^2^ < 0.767, Table 5). In a review of the best methods for correcting CGMs, an ATM monitor was compared to a reference PM_2.5_ instrument, the Teledyne T640.^74^ The results showed that the ATM had a lower correlation (R^2^ = 0.410, 24-h average) than what was reported by the SCAQMD and our study. Shittu et al. (2025) colocated eight ATM units with a reference-grade monitor over 18 weeks.^45^ Their results showed that ATMs had an *R* ^2^ > 0.700 for hourly averaged PM_2.5_. They concluded that a multiple linear regression calibration was sufficient to improve the ATMs’ PM_2.5_ measurements.

A field evaluation of the QAQ monitor done by SCAQMD showed that the PM_2.5_ data correlated well with FEM GRIMM data: 0.870 < R^2^ < 0.900.^76^ Their results also showed that the QAQ overestimated PM_2.5_ compared to the GRIMM at high concentrations. In our study, R^2^ for the QAQ’s PM_2.5_ data compared to GRIMM data ranged from 0.480 < R^2^ < 0.670 (1-h averages).

Colocation 2 demonstrated the weakest agreement with the GRIMM, both before and after model correction, as reflected in lower R^2^ values and higher RMSE and NRMSE metrics. Because this pattern was observed for both ATMs and QAQs, it is likely attributable to environmental conditions during this period rather than sensor malfunction. Colocation 2 was characterized by low PM_2.5_ concentrations, cold temperatures, and low relative humidity (Table 6; SI Figures S10 and S11). CL2 represents a low-signal “stress-test” scenario for optical PM_2.5_ monitors. Prior controlled and field evaluations have shown that optical scattering-based sensors exhibit reduced correlation with reference instruments at low ambient PM_2.5_ concentrations due to diminished signal-to-noise ratios, with performance degrading substantially below 20 μg m^–3^.^76–79^ Importantly, this behavior reflects performance limits under adverse environmental conditions rather than typical operating behavior and does not indicate systematic sensor failure. For clarity, comparisons to other colocation periods (e.g., CL1 or CL4) are not emphasized here, as CL2 is discussed specifically to illustrate performance under low-signal environmental conditions rather than to provide a relative ranking across deployments.

**Table 6:** ATM and QAQ Temperature, RH, and PM_2.5_ in Each Colocation

	Median ATM			Median QAQ		
Colocation	Temp (°C)	RH (%)	PM_2.5_ (μg m^–3^)	Temp (°C)	RH (%)	PM_2.5_ (μg m^–3^)
CL1	4.38	23.3	6.21	5.67	25.5	6.17
CL2	3.19	28.9	6.76	5.48	27.8	6.94
CL3	17.7	32.7	7.01	20.6	31.4	7.00
CL4	2.92	37.5	8.97	1.84	48.6	10.4

Figures 7 and 8 show scatterplots of PM_2.5_ data from the ATM and QAQ monitors compared to GRIMM data after applying the MLR correction model. The Pearson correlation matrices for the ATM and QAQ data compared to each other and to the GRIMM in each colocation after correction are provided in SI Figures S13 and S14 (ATM vs GRIMM, mean ρ = 0.818; QAQ vs GRIMM, mean ρ = 0.778). The correction process resulted in improved performance and more precise data from the CGMs. Note that the CGMs performed well even without any corrections (Table 3). In a study in California, three ATM monitors were colocated with DRX DustTrak 8533 Aerosol Monitors^59^ to measure roadside-related PM_2.5_.^80,81^ The study reported that the correlation coefficient comparing the ATMs and the DRX ranged ρ = 0.830–0.920, and that the ATMs presented good intermonitor correlation (*r* > 0.900),^75^ similar to our results.

**Figure 7:**
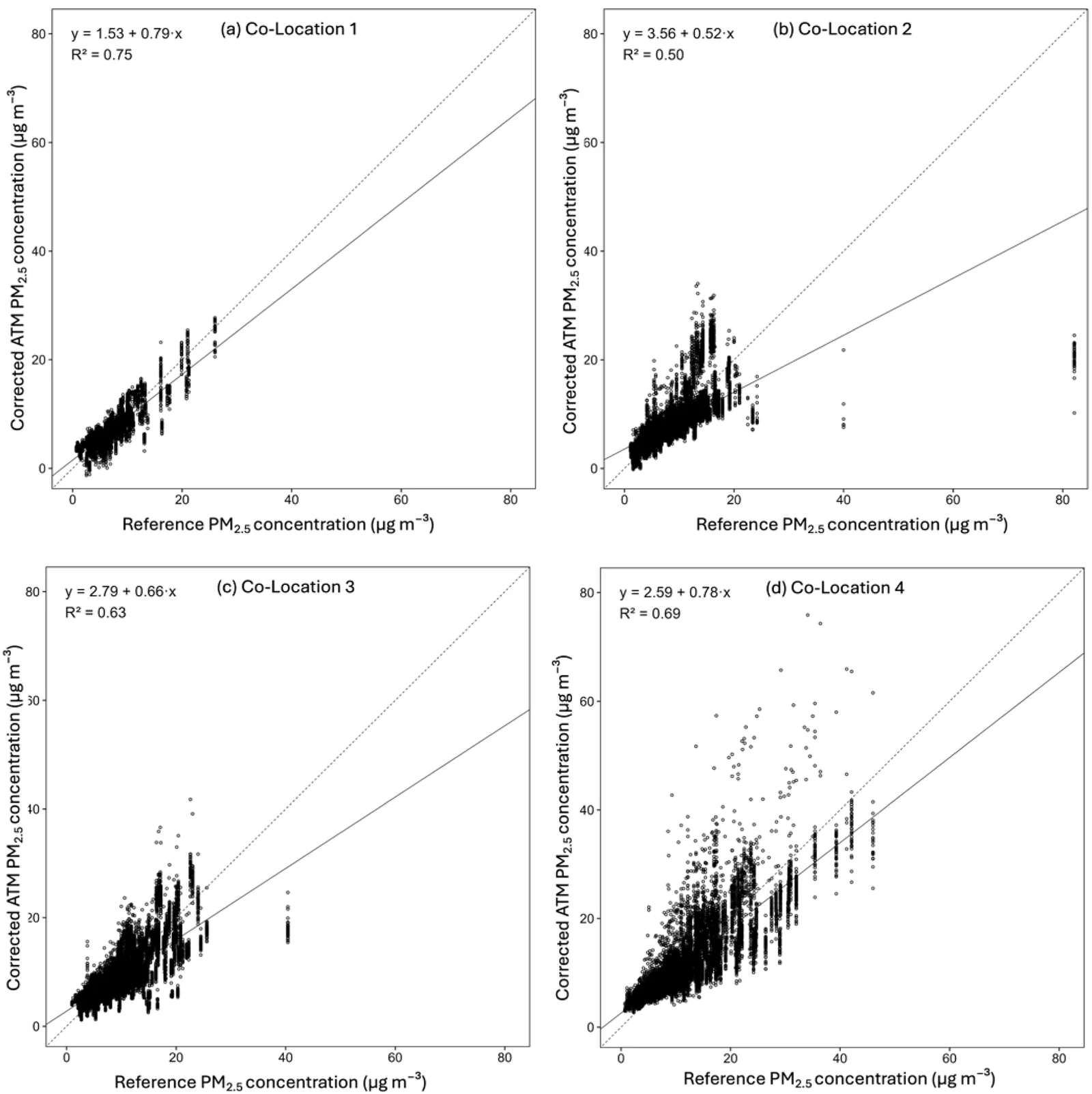
Scatterplots of corrected PM_2.5_ data from the ATM monitors (*y*-axis) versus the GRIMM reference instrument (*x*-axis) for colocations (a) 1, (b) 2, (c) 3, and (d) 4 after applying the multilinear regression correction model. The one-to-one line is shown on the graphs as dashed, and the linear regression model equation and R^2^ are shown on the plots.

**Figure 8:**
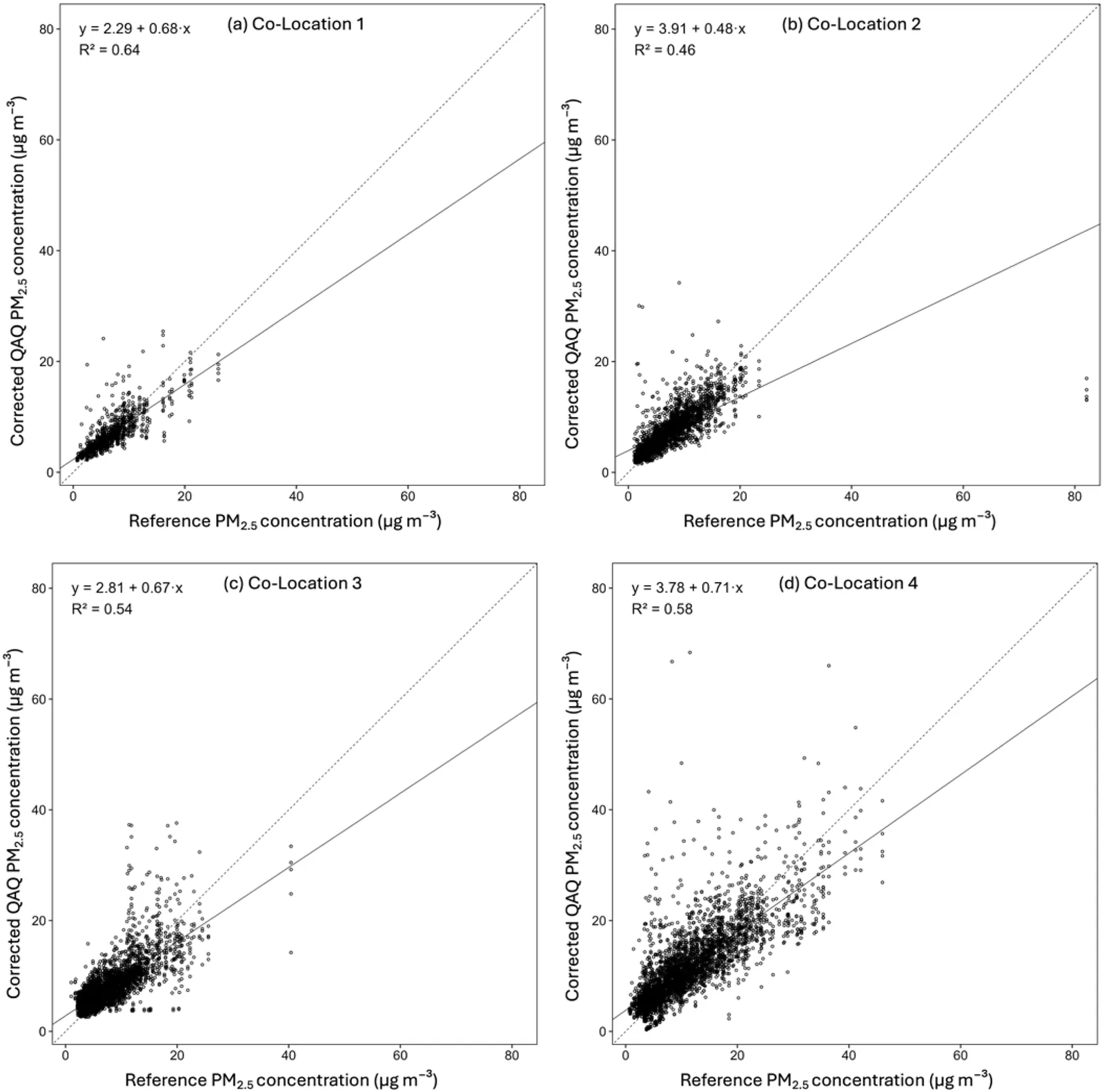
Scatterplots of corrected PM_2.5_ data from the QuantAQ monitors (*y*-axis) versus the GRIMM reference instrument (*x*-axis) for colocations (a) 1, (b) 2, (c) 3, and (d) 4 after applying the multilinear regression correction model. The one-to-one line is shown on the graphs as dashed, and the linear regression model equation and R^2^ are shown on the plots.

Atmotube monitors and QAQs have been used in several community air-quality studies, though not all have evaluated monitors’ performance or applied data corrections.^25,82–84^ Masri et al. (2022) used eight ATM monitors to measure workers’ PM_2.5_ exposure at an industrial facility in Santa Ana, California, and found elevated concentrations associated with welding activities.^85^ In a separate community-based study, the same group used 24 ATM monitors to characterize PM_2.5_ levels across residential and industrial neighborhoods in Santa Ana, reporting higher exposure concentrations in DI communities.^25^ In both cases, ATM monitors were selected based on the South Coast Air Quality Management District’s performance evaluation,^44^ but the authors did not independently validate monitor performance or correct the data.

Other studies have used QAQs. Yang et al. (2022) deployed QAQ monitors to measure PM concentrations and identify air pollutant sources,^50^ while Raheja et al. colocated two QAQs with a reference-grade Teledyne monitor in Accra, Ghana.^82^ Their results showed that QAQ PM_2.5_ measurements tracked the reference instrument and applying a multilinear regression model improved the hourly PM_2.5_ R^2^ values, consistent with the improvements observed in our study.

Zusman et al. (2020) derived regional calibration models for several U.S. cities by evaluating two widely used CGMs for particulate matter.^86^ They found that the calibration performance declined when models were applied outside the regions in which they were developed, largely because of differences in meteorological conditions. In our study, all colocations were conducted within the same region where the ATMs were ultimately deployed, but the comparisons were limited to outdoor PM_2.5_ measurements and did not include indoor or personal monitoring. Expanding future colocation studies to include indoor and personal exposure reference instruments would be an important advancement, as indoor environments have distinct pollution sources, such as cooking, cleaning, and personal care product use, and may also be influenced by mitigation strategies such as filtration.^87^

Colocation field studies involving CGMs with reference instruments may be required for assessing the accuracy and reliability of CGMs, though several issues can arise during such studies. Air quality can vary spatially and over time due to factors like traffic patterns, weather, and industrial activity within a city or region. Short-duration colocation studies may not capture such spatial and temporal variability adequately. And they can be time-consuming and expensive to complete. Correcting CGM data can be challenging, and improper correction can introduce bias into the data. Environmental factors such as humidity, temperature, and cross-interference from other gases can affect such monitors’ performance.^41,88,89^ Colocation studies must consider these factors when interpreting results.

In addition, the placement of monitors at the colocation site may influence the results. Factors such as the placement height, direct sunlight exposure, and potential obstructions to the sampling inlet must be considered. Consumer-grade monitors can degrade or become less accurate as they age.^90^ Long-term colocation studies may also need to account for changes in monitor performance over time.

Developing and applying a correction model for monitoring data may not always be necessary, especially when these monitors are intended for community-based applications. Communities may lack the resources and time required for comprehensive colocation studies, making deployment simplicity an important consideration. In this study, the raw PM_2.5_, temperature, and RH data were already well correlated with the FEM GRIMM instrument. Application of the correction model improved performance metrics; however, whether the magnitude of this improvement is consequential depends on the study, particularly in studies where relative changes and spatial or temporal contrasts are the primary objectives.

In our colocations, we identified defective monitors before deployment by colocating ATMs and QAQs with each other and applying Pearson correlation analyses. In addition to correlation-based methods, simpler approaches can be used to identify malfunctioning monitors, particularly in community-based deployments. Examples of simpler screening approaches include monitoring for time series with prolonged constant values (flat-lined readings), extreme deviations compared to nearby monitors, or unrealistic spikes and drops in PM_2.5_, temperature, or RH data. Observing device indicators, such as a white blinking LED signaling hardware failure, can also help to identify malfunctioning units. Additionally, comparing short-term trends by colocating devices with one another provides a practical way to assess consistency across monitors. Visual inspection of time series data and comparison across nearby devices offer an accessible and effective method for detecting faulty sensors without requiring an advanced statistical analysis. While Pearson’s correlation analysis was used in this study, these simpler field-based screening approaches can be effectively applied by community users.

Data cleaning processes should also be developed to handle erroneous data outside the sensors’ capabilities. Given the challenges, ensuring these monitors perform properly without a correction model might be practical for community use, thus empowering citizens and communities to actively participate in monitoring and improving air quality. Even without corrections, the monitors can help understand whether a user’s local outdoor or indoor environment is polluted or not, just with less accuracy, and can be used to indicate whether mitigation measures (e.g., air cleaners or exhausting the emissions from cooking) are effective when observing relative changes in the concentrations. This citizen science approach also enhances realistic data collection. Colocation with federal monitors should not be a burden for the citizen scientist, and it is the research and governmental institutions’ duty to ensure that such devices perform properly compared to reference monitors by implementing long-term and short-term regional colocation studies.

## Environmental Implications

In this study, we present a large-scale, multisession field evaluation of consumer-grade air quality monitors measuring PM_2.5_, conducted as a foundational methodological component of a community exposure research project. By colocating a large number of personal exposure and outdoor monitors prior to deployment, this work provides a practical framework for evaluating data quality, identifying defective units, and characterizing performance variability in community-based air quality studies. The central question addressed is not whether these monitors achieve regulatory-grade accuracy but how they perform under real-world deployment conditions and what validation steps are necessary to support their use in studies of personal exposure. The approach and findings are intended to inform researchers and community partners seeking to design, validate, and interpret similar monitoring efforts under realistic resource and infrastructure constraints.

We applied a correction model previously established and recommended by the U.S EPA with the aim of evaluating its effectiveness under local deployment conditions. Application of the model improved agreement with the reference instrument; however, the extent to which such a correction is necessary depends on study objectives, environmental conditions, and available resources. In settings where absolute concentration accuracy is required, reference-based correction may be needed, while in other contexts, such as studies emphasizing relative exposure comparisons or temporal trends or shorter study periods, predeployment screening and performance evaluation may be sufficient. The effectiveness of any correction approach remains environment- and monitor-specific, consistent with the existing EPA guidance. The correction coefficients and drift rates reported in this study are specific to the local environment, time period, and deployment conditions evaluated here and should not be assumed to be directly transferable to other regions or monitoring contexts.

In the absence of a regulatory air monitoring site, our results indicate that colocating CGMs with one another provides a practical approach for identifying malfunctioning units and assessing relative performance. Testing monitors under local environmental conditions allows researchers to account for factors influencing the sensor behavior prior to deployment. In situations where reference instruments are unavailable, intercomparison among CGMs can offer valuable insight into device performance and data reliability. While correction of raw data can improve agreement with reference measurements, the ATM and QAQ monitors demonstrated strong intermonitor correlation even without correction, supporting use that is adequate for community-based exposure studies. Our study also shows that longer-term deployments will benefit from intercomparison and replacement efforts to minimize bias, after 1–1.5 years. Overall, this study contributes to a clearer understanding of the strengths and limitations of CGMs and provides practical guidance for the responsible deployment and interpretation of air-quality data in environmental justice community-based research.

## Supplementary Material



## Notes

Description of colocation monitor setup (Figures S1–S4); correlation matrices before and after quality-control screening (Figures S5–S7); Bland–Altman and sensor drift analyses (Figures S8–S9); temperature and relative humidity comparisons (Figures S10–S11); PM2.5 distributions (Figure S12); corrected-data correlation matrices (Figures S13–S14); performance equations; and Tables S1–S23 containing quality-control statistics, monitor performance metrics, and correction model parameters (PDF)
