# Supplementary material for: Validation and Correction of PM2.5 Mass Concentrations from Consumer-Grade Personal Exposure and Outdoor Fixed Monitors: A Two-Year Colocation Study in Colorado
Source: ACS Environ Au. 2026 Aug 4;6(5):798–813. doi: 10.1021/acsenvironau.6c00020 (PMC13602343; doi:10.1021/acsenvironau.6c00020)
Supplement: Supplementary file 1 [file vg6c00020_si_001.pdf]

Supporting Information

Validation and Correction of $PM_{2.5}$ Mass Concentrations from Consumer-Grade Personal Exposure and Outdoor Fixed Monitors: A Two-Year Colocation Study in Colorado

Aniya Khalili Hollo[1], Dulce Gonzalez-Beltran[1], Allison Heckman[1], Sophie Castillo[2], Nicholas Clements[1], Esther Sullivan[3], Shivakant Mishra[4], and Shelly L. Miller[1,*]

[1]Department of Mechanical Engineering, University of Colorado Boulder, 427 UCB, 1111 Engineering Drive, Boulder, Colorado 80309, United States

[2]Environmental Engineering Program, 4001 Discovery Drive, University of Colorado Boulder, Boulder, Colorado 80309, United States

[3]Department of Sociology, University of Colorado Denver, 1380 Lawrence Street, Suite 420, Denver, Colorado 80204, United States

[4]Department of Computer Science, University of Colorado Boulder, 430 UCB, 1111 Engineering Drive, Boulder, Colorado 80309, United States

[*]Corresponding author info: Shelly.Miller@colorado.edu

Keywords: [fine particulate matter], [low-cost sensor], [urban air pollution], [monitor performance], [air quality monitoring], [personal exposure], [sensor evaluation]

List of SI Figures

Figure S1: Photos of the setup used in co-locations two, three, and four: a1) tarp covering the ATM monitors, a2) ATMs placed on top of crates to elevate from the rooftop (2.74 m high), b1) ATMs secured on the crates under the tarp, c1) QAQs secured with zip ties, and c2) QAQs mounted on a tripod. 1
Figure S2: Side view of the setup used in co-locations two, three, and four: a) tarp covers the ATM monitors from harsh weather conditions, b) ATM monitors were located on the top of crates under the tarp, c) crates to elevate ATMs from the rooftop, and d) I-25 Highway.. 2
Figure S3: Photos of the QAQ monitors' position during co-location 3 at the I-25 Globeville site, Denver, Colorado... 3
Figure S4: Instructions for syncing ATM data to the cloud using the sync option in the ATM app. ***Error! Bookmark not defined.***
Figure S5: For CL3, (a) the Pearson correlation matrix that was used to identify defective Atmotube Pro monitors; and (b) the recalculated Pearson correlation matrix with the defective monitor data removed. The defective Atmotube PRO monitors were 12, 14, 46, 50, 56. 5
Figure S6: Correlation matrix (before applying correction model) on cleaned 1-hour averaged ATMs $PM_{2.5}$ data for each co-location: a) CL1, b) CL2, c) CL3, and d) CL4... 9
Figure S7: Correlation matrix before applying correction model on cleaned 1-hour averaged QAQ $PM_{2.5}$ data. Data for each co-location: a) CL1, b) CL2, c) CL3, and d) CL4. 13
Figure S8: Bland–Altman plot comparing QAQ and reference $PM_{2.5}$ measurements. 14
Figure S9: Drift (µg m^{-3}/day) for individual ATM $PM_{2.5}$ by deployment period.. 15
Figure S10: Comparison between the ATM and CDPHE GRIMM data of the 1-hour averaged (a) temperature (°C) and (b) RH (%) for each co-location.. 16
Figure S11: Comparison between the QAQ and CDPHE GRIMM data of the 1-hour averaged temperature (℃) and RH (%) for each co-location... 17
Figure S12: Box plots showing the distribution of cleaned hourly averaged $PM_{2.5}$ data for (a) ATM monitors, (b) QAQ monitors, and (c) GRIMM monitor (bottom). 18
Figure S13: Correlation matrix after applying MLR model to ATM $PM_{2.5}$ data for each co-location after correction. a) CL1, b) CL2, c) CL3, and d) CL4. 23
Figure S14: Correlation matrix after applying MLR model on QAQ $PM_{2.5}$ data for each co-location after correction. a) CL1, b) CL2, c) CL3, and d) CL4. 29

List of SI Equations

Coefficient of Determination Eq. (S1). 26
Linear Regression Slope Eq. (S2). 26
Linear Regression y-Intercept Eq. (S3). 26
Root Mean Square Error RMSE Eq. (S4). 26
Normalized Root Mean Square Error Eq. (S5). 26

List of SI Tables

Table S1: Percentage of RH Data <10% and >80% Excluded in Each Co-location after Removing the Defective Monitors.....31
Table S2: Summary of Atmotube Monitors' Temperature and RH Data at Each Co-location (CL).....31
Table S3: Summary of CDPHE GRIMM Monitors' Temperature and RH Data at Each Co-location.....31
Table S4: Summary of QuantAQ Monitors' Temperature and RH Data at Each Co-location.....32
Table S5: Summary of Atmotube Monitors' $PM_{2.5}$ Data at Each Co-location.....33
Table S6: Summary of CDPHE GRIMM Monitors' $PM_{2.5}$ Data at Each Co-location.....33
Table S7: Summary of QuantAQ Monitors' $PM_{2.5}$ Data at Each Co-location.....33
Table S8: Mean Performance Metrics Comparing Individual ATM Data with Grimm Data at the I-25 Globeville Co-location Site (CL1).....34
Table S9: Mean Performance Metrics Comparing Individual ATM Data with Grimm Data at the I-25 Globeville Co-location Site (CL2).....35
Table S10: Mean Performance Metrics Comparing Individual ATM Data with Grimm Data at the I-25 Globeville Co-location Site (CL3).....36
Table S11: Mean Performance Metrics Comparing Individual ATM Data with Grimm Data at the I-25 Globeville Co-location Site (CL4).....38
Table S12: Mean Performance Metrics Comparing Individual QAQ Data with Grimm Data at the I-25 Globeville Co-location Site (CL1).....39
Table S13: Mean Performance Metrics Comparing Individual QAQ Data with Grimm Data at the I-25 Globeville Co-location Site (CL2).....40
Table S14: Mean Performance Metrics Comparing Individual QAQ Data with Grimm Data at the I-25 Globeville Co-location Site (CL3).....40
Table S15: Mean Performance Metrics Comparing Individual QAQ Data with Grimm Data at the I-25 Globeville Co-location Site (CL4).....41
Table S16: Model Performance Metrics for One QAQ Monitor (Number 207) from CL2 Using Four Different Multilinear Regression Models.....41
Table S17: Model Performance Metrics for One ATM Monitor (ATM-42) from CL2 Using Four Different Multilinear Regression Models.....42
Table S18: Parameters Used in the MLR Correction Model Based on Individual QAQ Coefficients for CL2.....42
Table S19: Model Parameters for Each QAQ After Correction Based on Individual QAQ Coefficients for CL2.....43
Table S20: Model Results for Correcting QAQ $PM_{2.5}$ Data Based on Individual vs. Mean Values in CL2.....43
Table S21: Parameters Used in the MLR Correction Model Based on Individual ATM Coefficients for CL2.....44
Table S22: Model Parameters for Each ATMs after Correction Based on Individual ATM Coefficients for CL2.....45
Table S23: Model Results for Correcting ATM $PM_{2.5}$ Data Based on Individual vs. Mean Values in CL2.....47

SI Figures

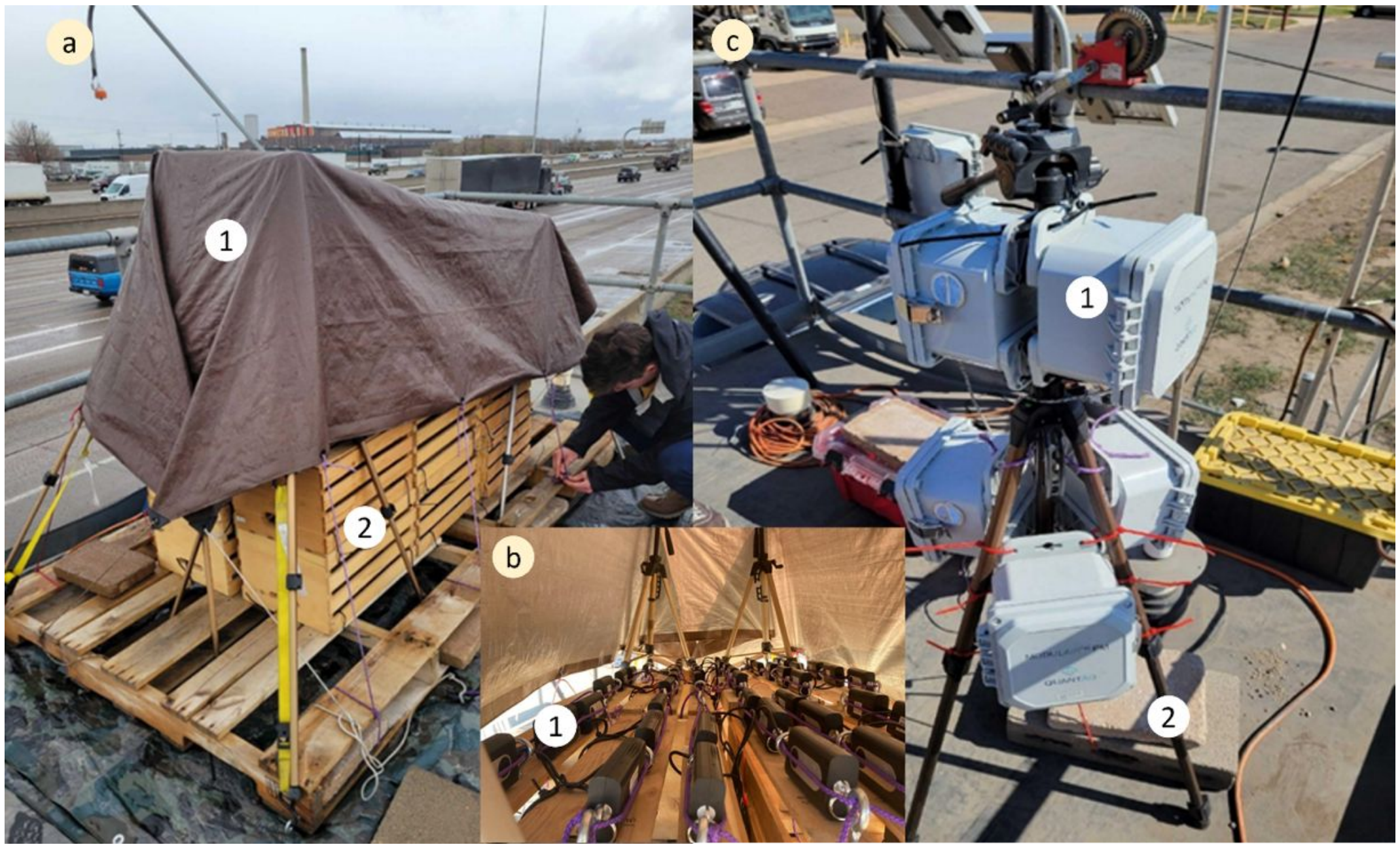

Figure S1: Photos of the setup used in co-locations two, three, and four: a1) tarp covering the ATM monitors, a2) ATMs placed on top of crates to elevate from the rooftop (2.74 m high), b1) ATMs secured on the crates under the tarp, c1) QAQs secured with zip ties, and c2) QAQs mounted on a tripod.

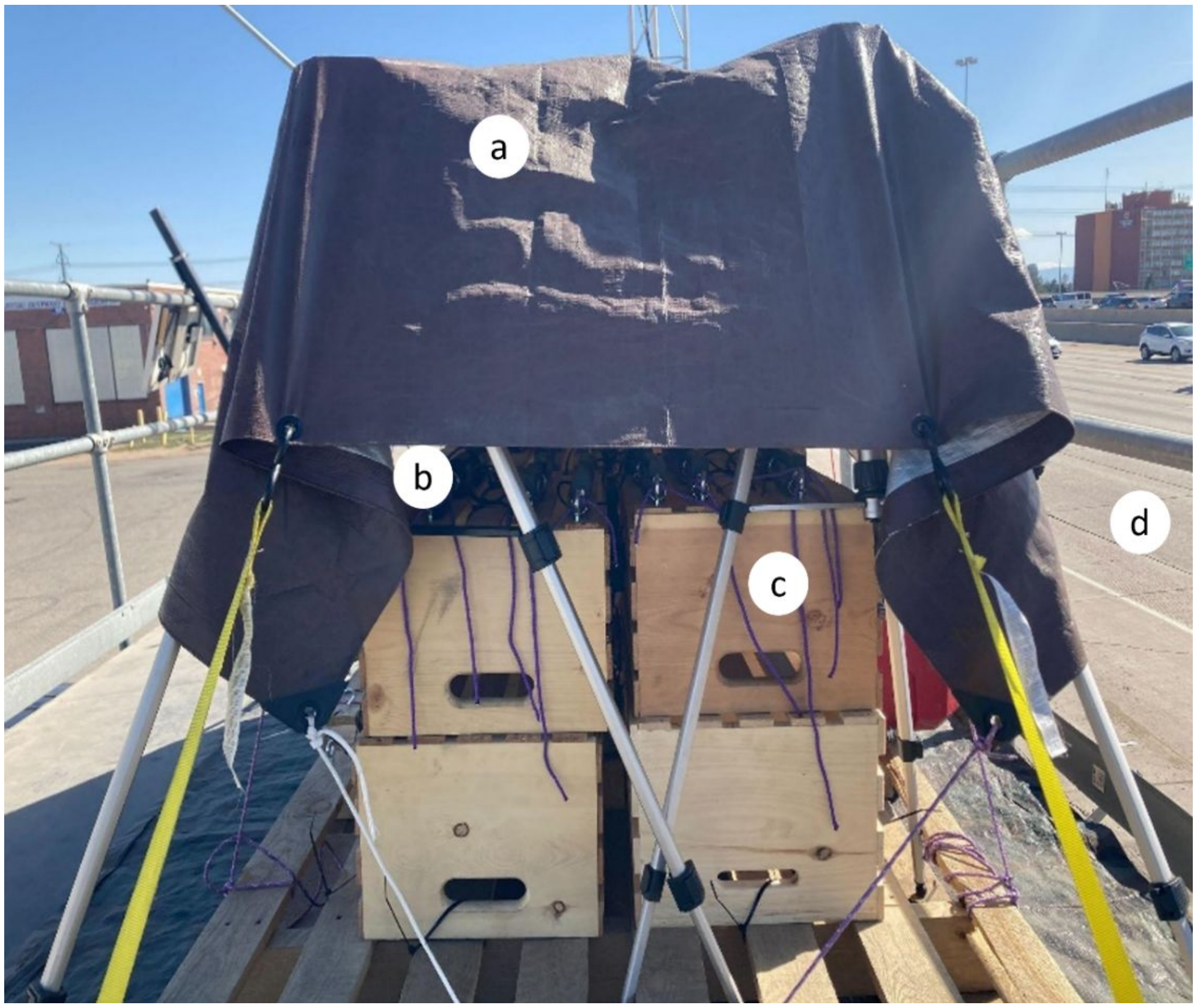

Figure S2: Side view of the setup used in co-locations two, three, and four: a) tarp covers the ATM monitors from harsh weather conditions, b) ATM monitors were located on the top of crates under the tarp, c) crates to elevate ATMs from the rooftop, and d) I-25 Highway.

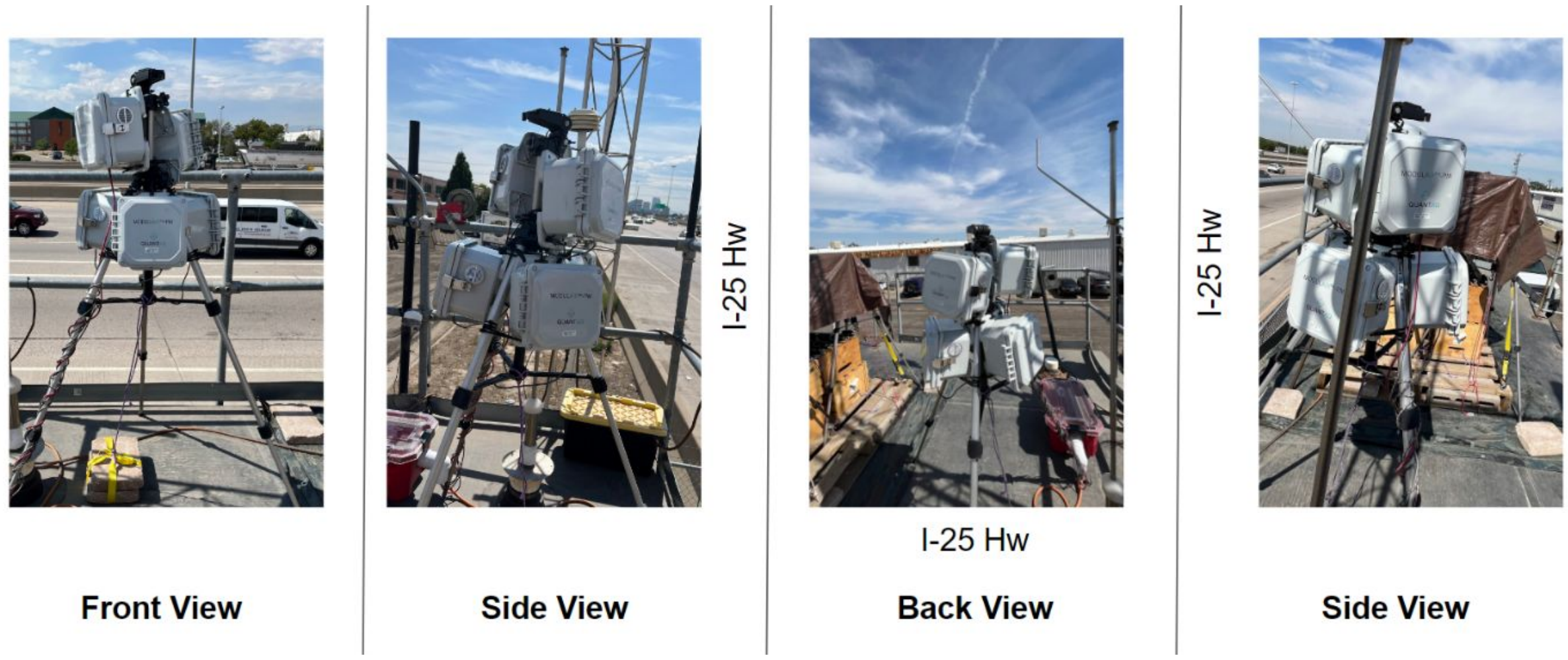

Figure S3: Photos of the QAQ monitors' position during co-location 3 at the I-25 Globeville site, Denver, Colorado.

- To synchronize, make sure your smartphone's Bluetooth is on, and under the section Data Synchronization turn on 'Upload historical data to the cloud.'
- Once Bluetooth is turned on, your Atmotube will automatically locate your smartphone and pair it with it.
- Open the Atmotube application on your smartphone and set your Atmotube next to your smartphone.
- IMPORTANT: 'Synchronizing #% will appear on the homepage of the application screen above "Average air quality score." It is particularly important that the syncing process reaches 100%. If it appears to stall, walk around until you can find a better Wi-Fi signal or cellular data connection to complete the sync. PLEASE NOTE:

Cellular data of 3 bars or higher is required to complete this step. Wi-Fi with 2 bars is required to complete this step.

Figure S4: Instructions for syncing ATM data to the cloud using the sync option in the ATM app.

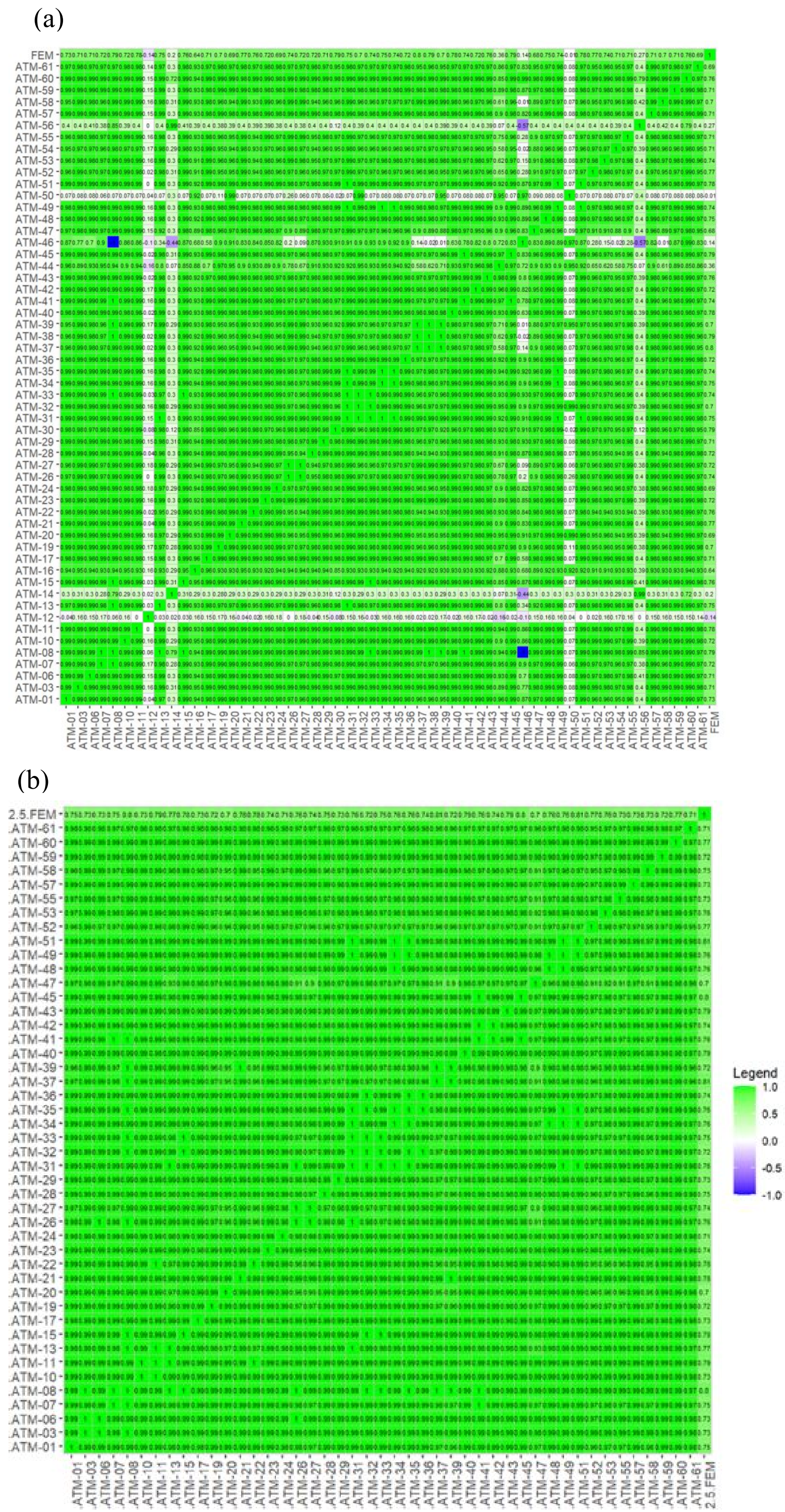

Figure S5: For CL3, (a) the Pearson correlation matrix that was used to identify defective Atmotube Pro monitors; and (b) the recalculated Pearson correlation matrix with the defective monitor data

(a)

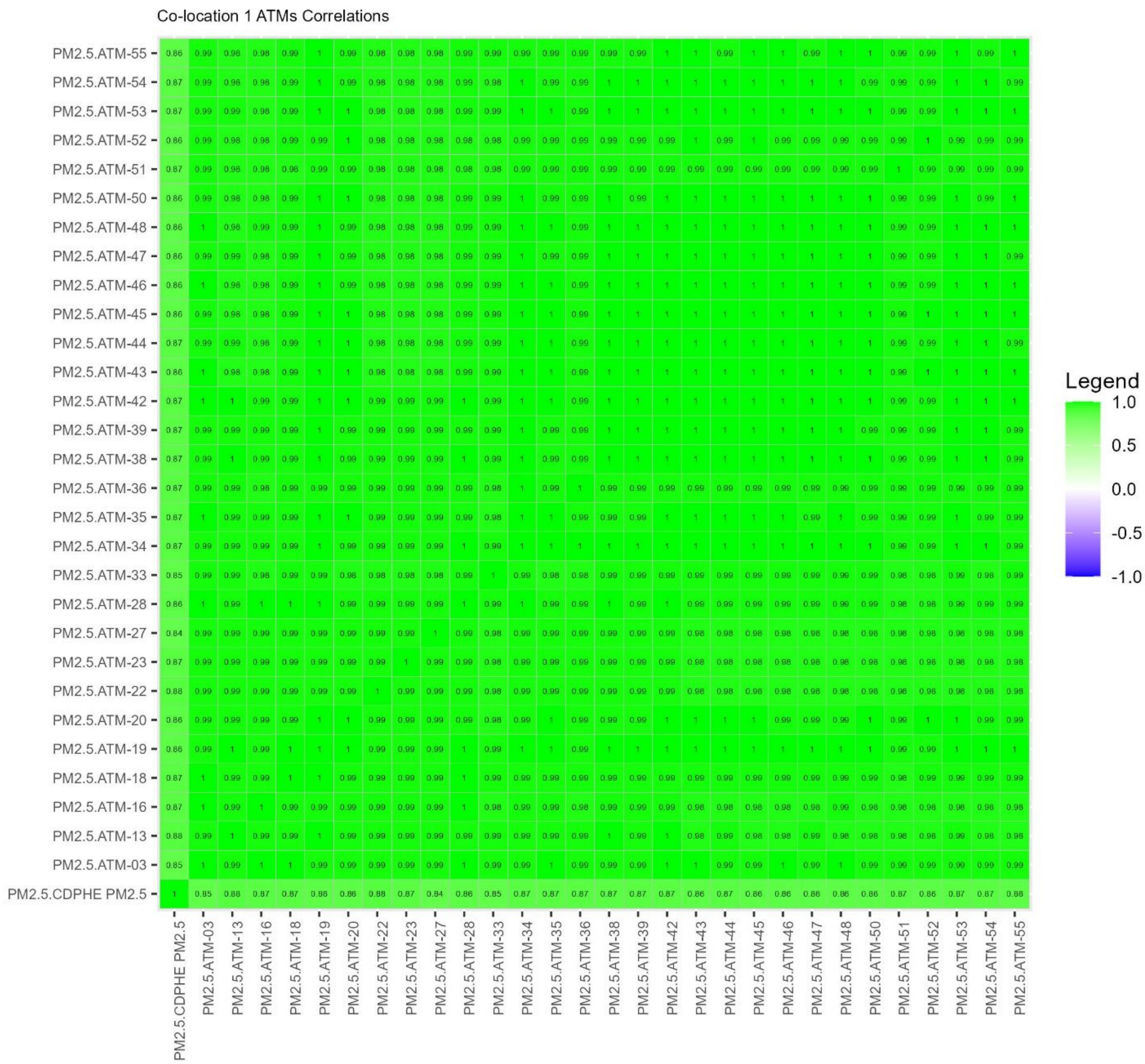
Co-location 1 ATMs Correlations
PM2.5.ATM-55
PM2.5.ATM-54
PM2.5.ATM-53
PM2.5.ATM-52
PM2.5.ATM-51
PM2.5.ATM-50
PM2.5.ATM-48
PM2.5.ATM-47
PM2.5.ATM-46
PM2.5.ATM-45
PM2.5.ATM-44
PM2.5.ATM-43
PM2.5.ATM-42
PM2.5.ATM-39
PM2.5.ATM-38
PM2.5.ATM-36
PM2.5.ATM-35
PM2.5.ATM-34
PM2.5.ATM-33
PM2.5.ATM-28
PM2.5.ATM-27
PM2.5.ATM-23
PM2.5.ATM-22
PM2.5.ATM-20
PM2.5.ATM-19
PM2.5.ATM-18
PM2.5.ATM-16
PM2.5.ATM-13
PM2.5.ATM-03
PM2.5.CDPHE PM2.5
Legend
1.0
0.5
0.0
-0.5
-1.0

(b)

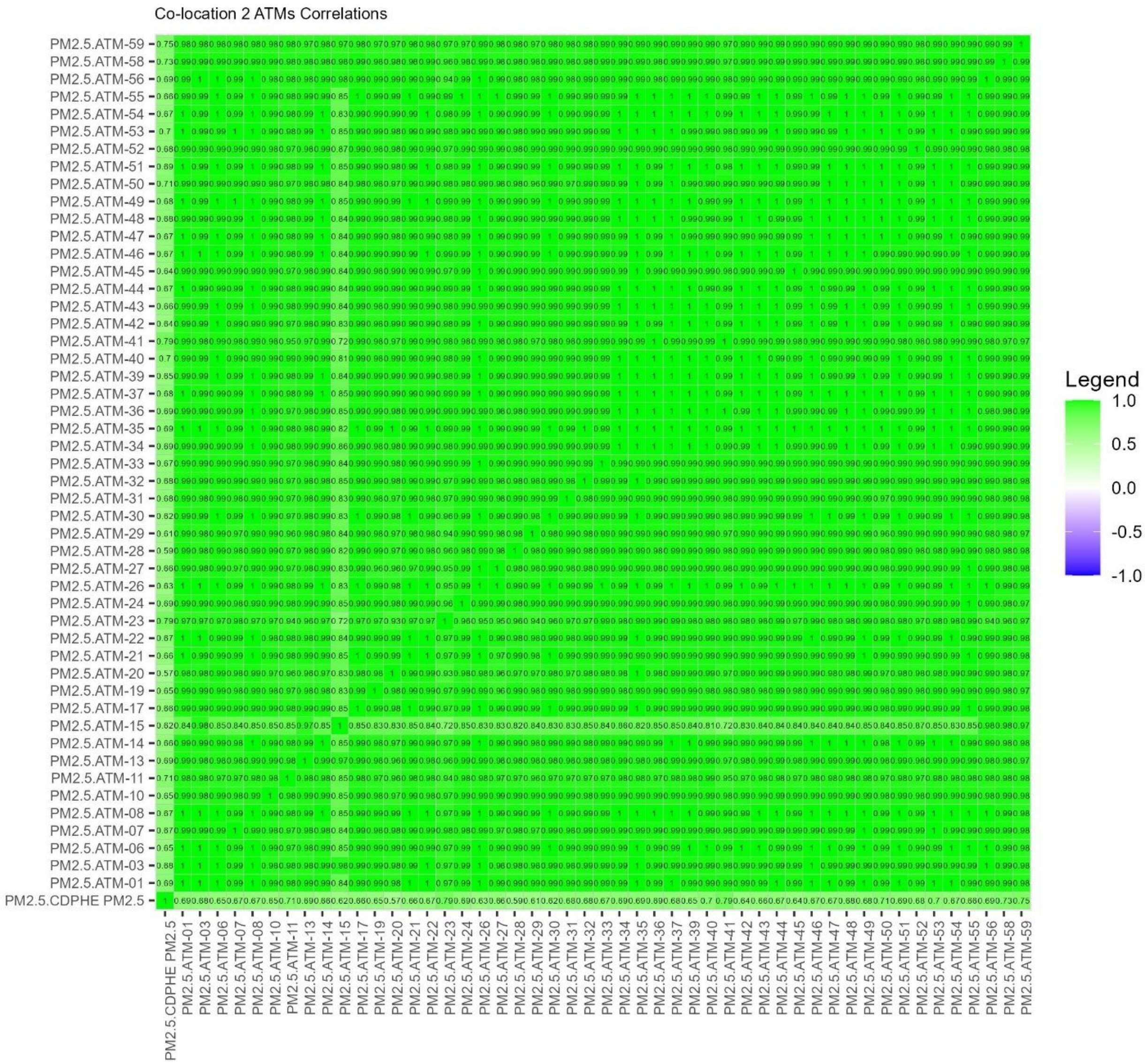
Co-location 2 ATMs Correlations
Legend
1.0
0.5
0.0
-0.5
-1.0
PM2.5.ATM-59
PM2.5.ATM-58
PM2.5.ATM-56
PM2.5.ATM-55
PM2.5.ATM-54
PM2.5.ATM-53
PM2.5.ATM-52
PM2.5.ATM-51
PM2.5.ATM-50
PM2.5.ATM-49
PM2.5.ATM-48
PM2.5.ATM-47
PM2.5.ATM-46
PM2.5.ATM-45
PM2.5.ATM-44
PM2.5.ATM-43
PM2.5.ATM-42
PM2.5.ATM-41
PM2.5.ATM-40
PM2.5.ATM-39
PM2.5.ATM-37
PM2.5.ATM-36
PM2.5.ATM-35
PM2.5.ATM-34
PM2.5.ATM-33
PM2.5.ATM-32
PM2.5.ATM-31
PM2.5.ATM-30
PM2.5.ATM-29
PM2.5.ATM-28
PM2.5.ATM-27
PM2.5.ATM-26
PM2.5.ATM-24
PM2.5.ATM-23
PM2.5.ATM-22
PM2.5.ATM-21
PM2.5.ATM-20
PM2.5.ATM-19
PM2.5.ATM-17
PM2.5.ATM-15
PM2.5.ATM-14
PM2.5.ATM-13
PM2.5.ATM-11
PM2.5.ATM-10
PM2.5.ATM-08
PM2.5.ATM-07
PM2.5.ATM-06
PM2.5.ATM-03
PM2.5.ATM-01
PM2.5.CDPHE PM2.5

(c)

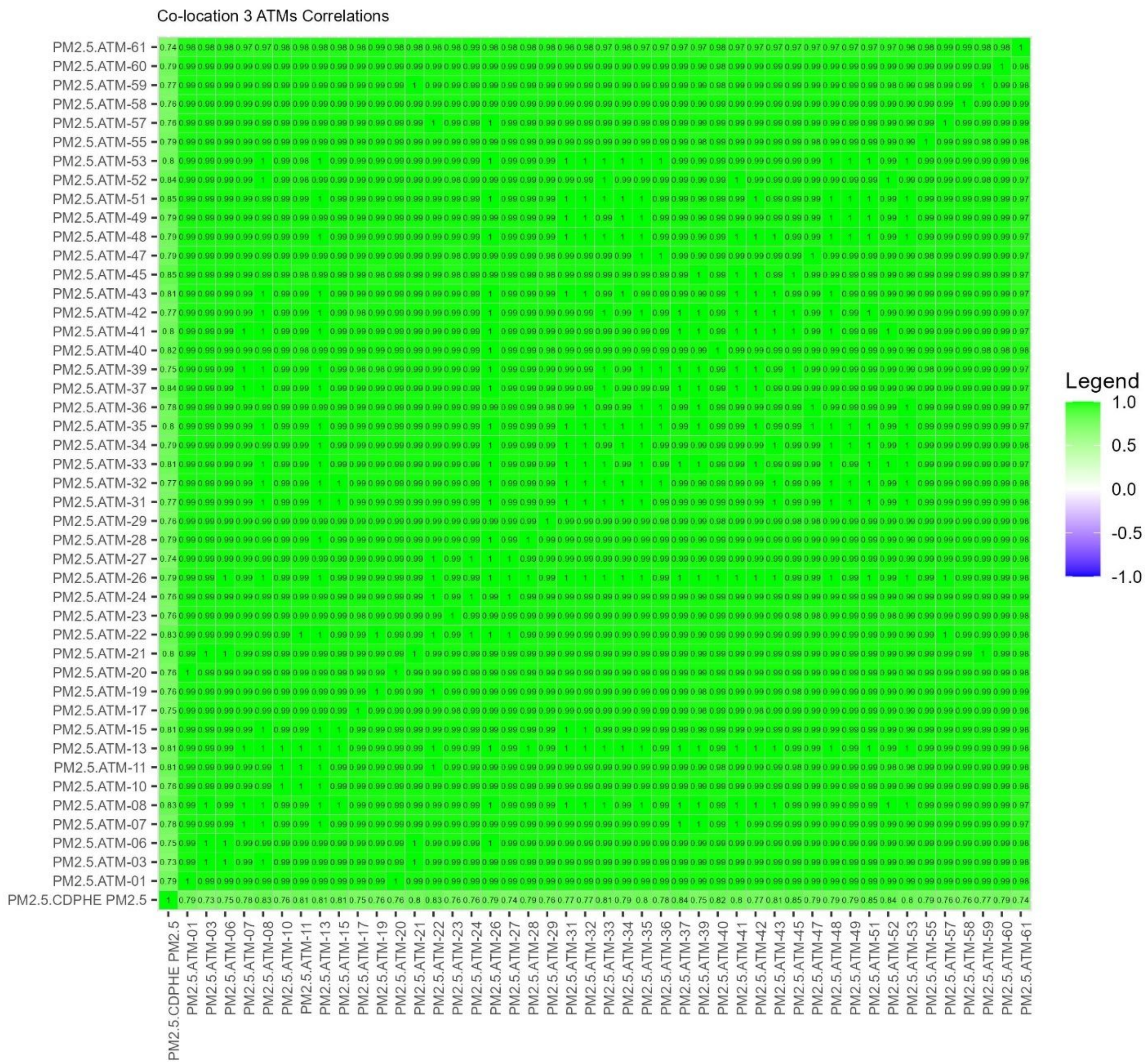
Co-location 3 ATMs Correlations
Legend
1.0
0.5
0.0
-0.5
-1.0
PM2.5.ATM-61
PM2.5.ATM-60
PM2.5.ATM-59
PM2.5.ATM-58
PM2.5.ATM-57
PM2.5.ATM-55
PM2.5.ATM-53
PM2.5.ATM-52
PM2.5.ATM-51
PM2.5.ATM-49
PM2.5.ATM-48
PM2.5.ATM-47
PM2.5.ATM-45
PM2.5.ATM-43
PM2.5.ATM-42
PM2.5.ATM-41
PM2.5.ATM-40
PM2.5.ATM-39
PM2.5.ATM-37
PM2.5.ATM-36
PM2.5.ATM-35
PM2.5.ATM-34
PM2.5.ATM-33
PM2.5.ATM-32
PM2.5.ATM-31
PM2.5.ATM-29
PM2.5.ATM-28
PM2.5.ATM-27
PM2.5.ATM-26
PM2.5.ATM-24
PM2.5.ATM-23
PM2.5.ATM-22
PM2.5.ATM-21
PM2.5.ATM-20
PM2.5.ATM-19
PM2.5.ATM-17
PM2.5.ATM-15
PM2.5.ATM-13
PM2.5.ATM-11
PM2.5.ATM-10
PM2.5.ATM-08
PM2.5.ATM-07
PM2.5.ATM-06
PM2.5.ATM-03
PM2.5.ATM-01
PM2.5.CDPHE PM2.5

(d)

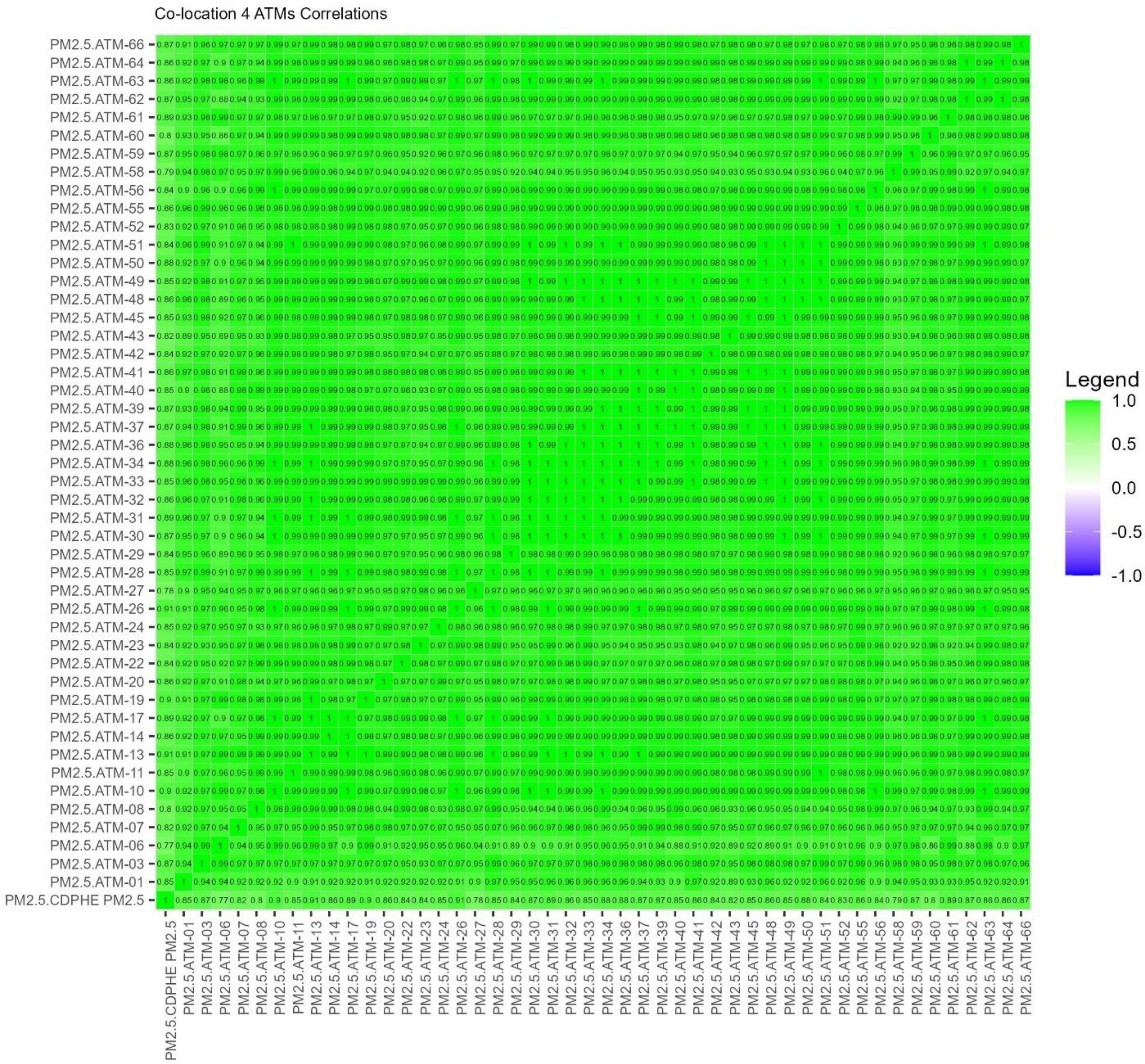

Figure S6: Correlation matrix (before applying correction model) on cleaned 1-hour averaged ATMs $PM_{2.5}$ data for each co-location: a) CL1, b) CL2, c) CL3, and d) CL4.

(a)

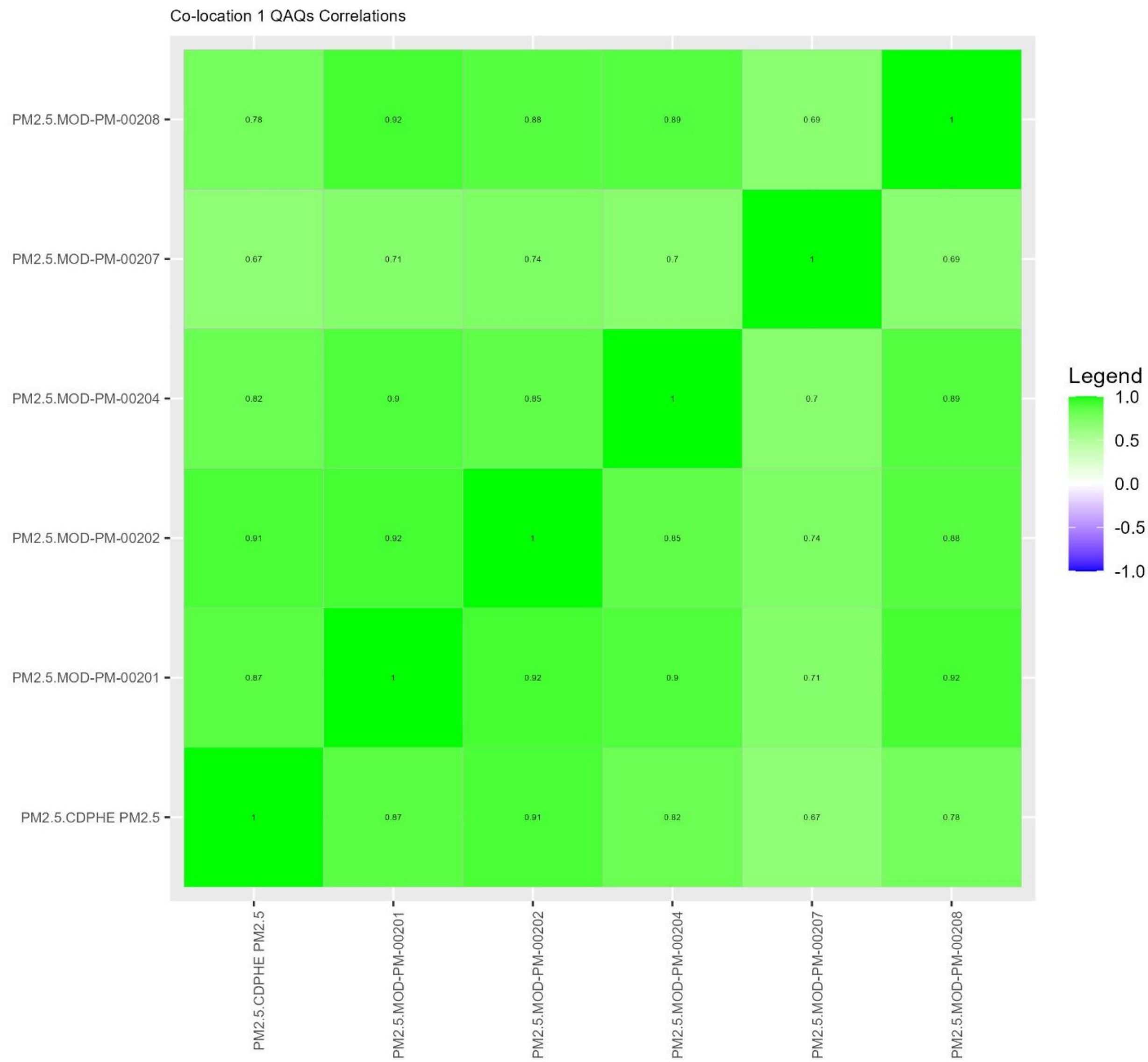
Co-location 1 QAQs Correlations
PM2.5.MOD-PM-00208
PM2.5.MOD-PM-00207
PM2.5.MOD-PM-00204
PM2.5.MOD-PM-00202
PM2.5.MOD-PM-00201
PM2.5.CDPHE PM2.5
0.78
0.92
0.88
0.89
0.69
1
0.67
0.71
0.74
0.7
1
0.69
0.82
0.9
0.85
1
0.7
0.89
0.91
0.92
1
0.85
0.74
0.88
0.87
1
0.92
0.9
0.71
0.92
1
0.87
0.91
0.82
0.67
0.78
Legend
1.0
0.5
0.0
-0.5
-1.0

(b)

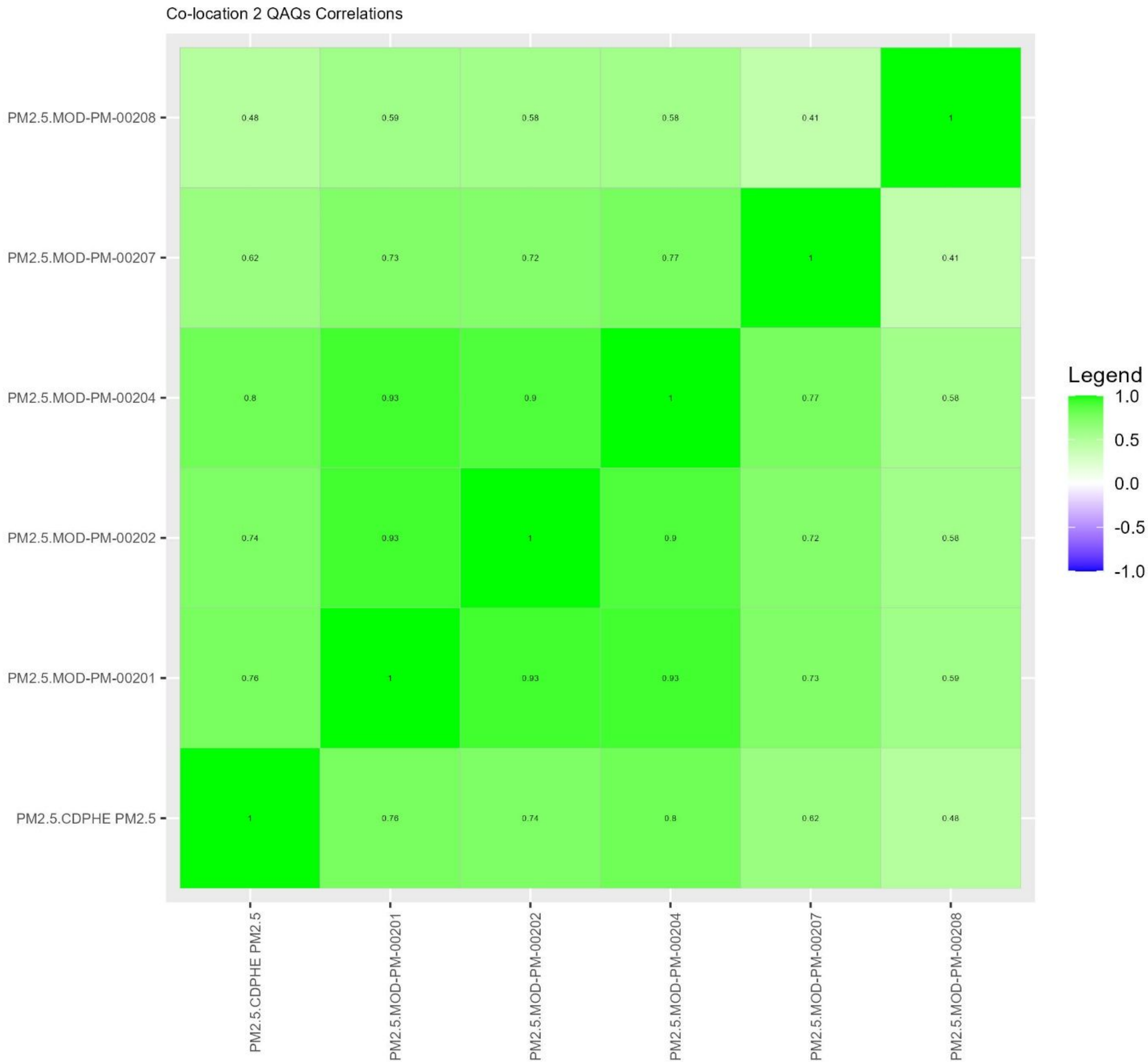
Co-location 2 QAQs Correlations
PM2.5.MOD-PM-00208
0.48
0.59
0.58
0.58
0.41
1
PM2.5.MOD-PM-00207
0.62
0.73
0.72
0.77
1
0.41
PM2.5.MOD-PM-00204
0.8
0.93
0.9
1
0.77
0.58
PM2.5.MOD-PM-00202
0.74
0.93
1
0.9
0.72
0.58
PM2.5.MOD-PM-00201
0.76
1
0.93
0.93
0.73
0.59
PM2.5.CDPHE PM2.5
1
0.76
0.74
0.8
0.62
0.48
PM2.5.CDPHE PM2.5
PM2.5.MOD-PM-00201
PM2.5.MOD-PM-00202
PM2.5.MOD-PM-00204
PM2.5.MOD-PM-00207
PM2.5.MOD-PM-00208
Legend
1.0
0.5
0.0
-0.5
-1.0

(c)

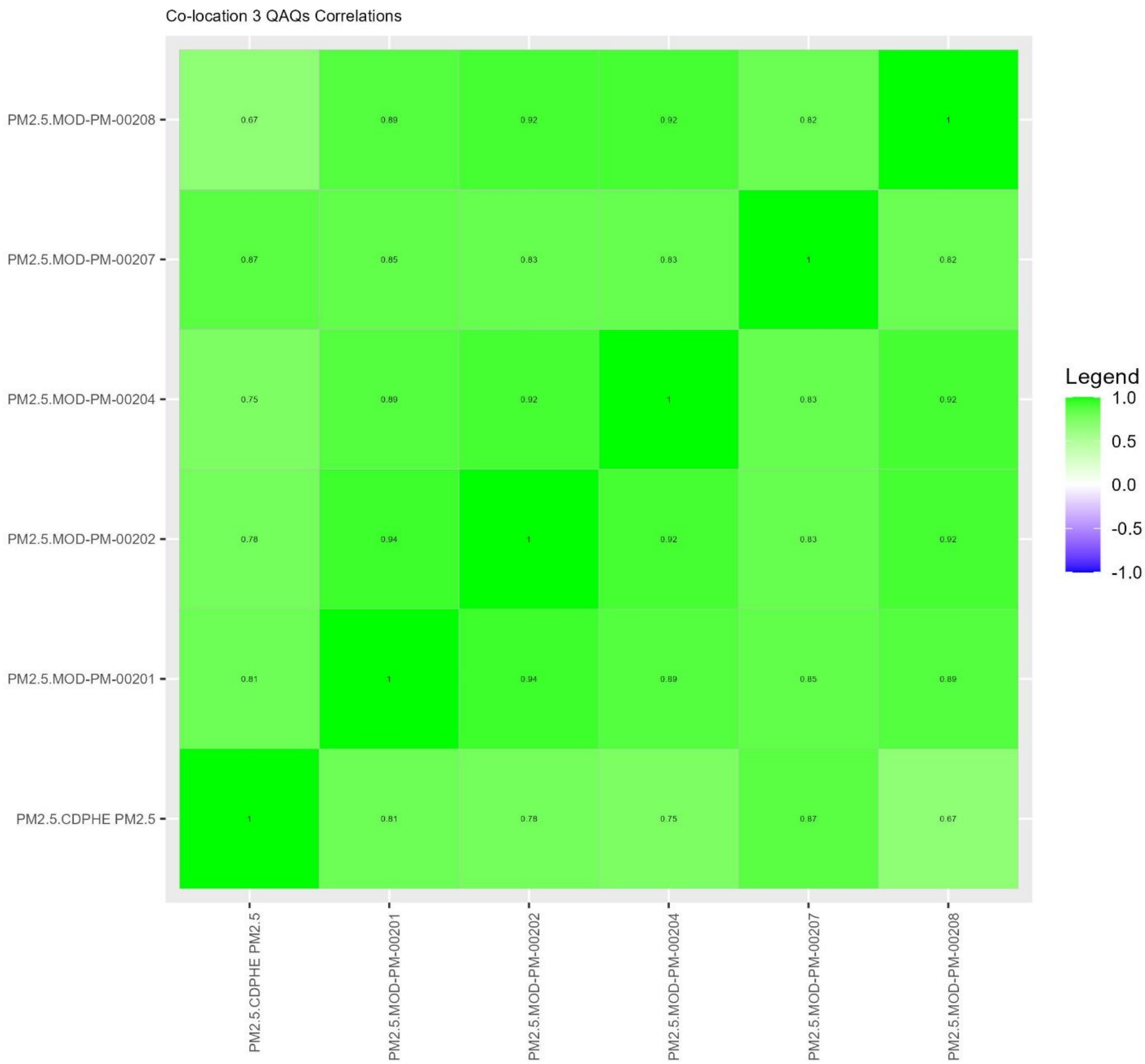
Co-location 3 QAQs Correlations
PM2.5.MOD-PM-00208
PM2.5.MOD-PM-00207
PM2.5.MOD-PM-00204
PM2.5.MOD-PM-00202
PM2.5.MOD-PM-00201
PM2.5.CDPHE PM2.5
Legend
1.0
0.5
0.0
-0.5
-1.0

(d)

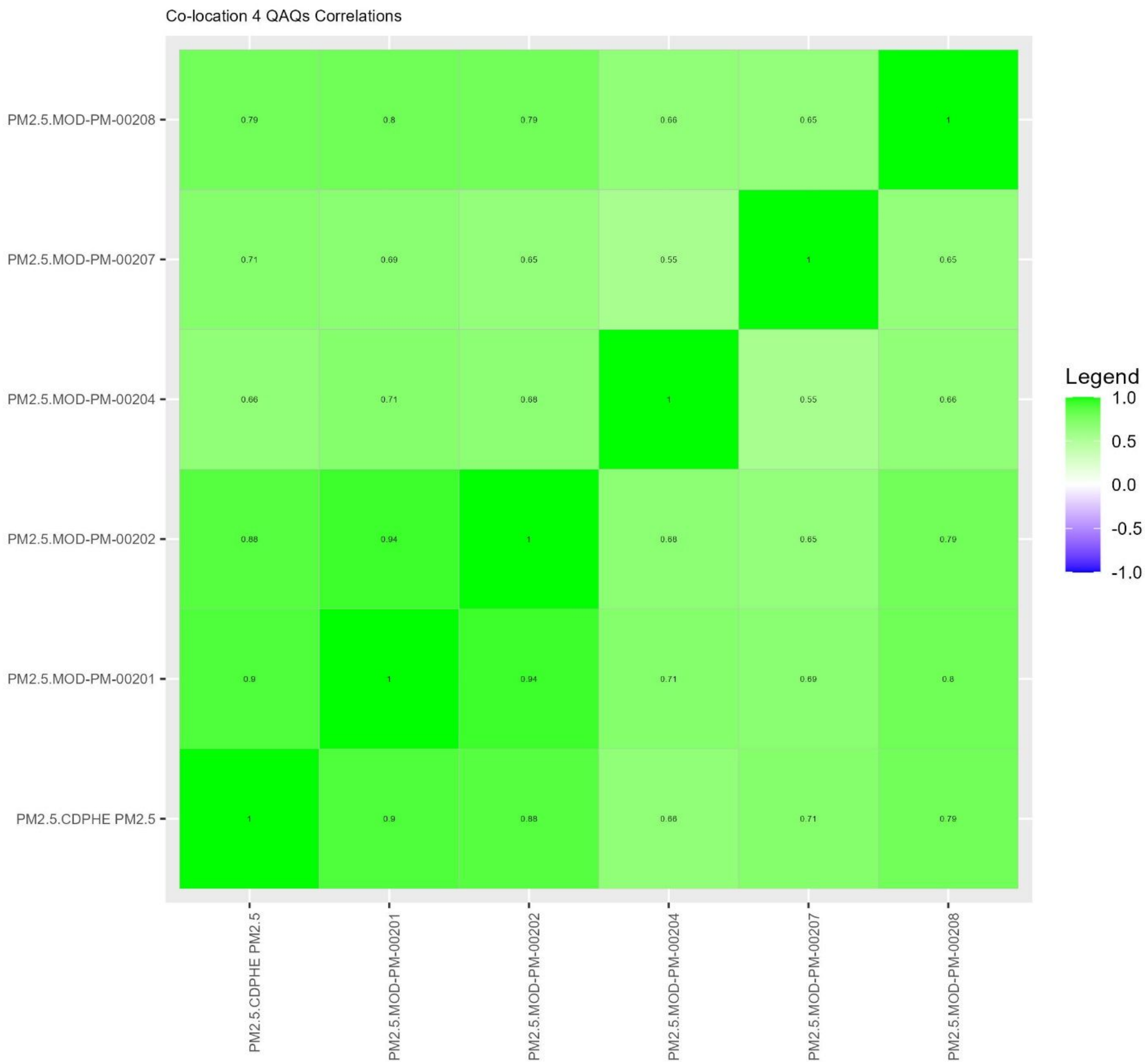

Figure S7: Correlation matrix before applying correction model on cleaned 1-hour averaged QAQ $PM_{2.5}$ data. Data for each co-location: a) CL1, b) CL2, c) CL3, and d) CL4.

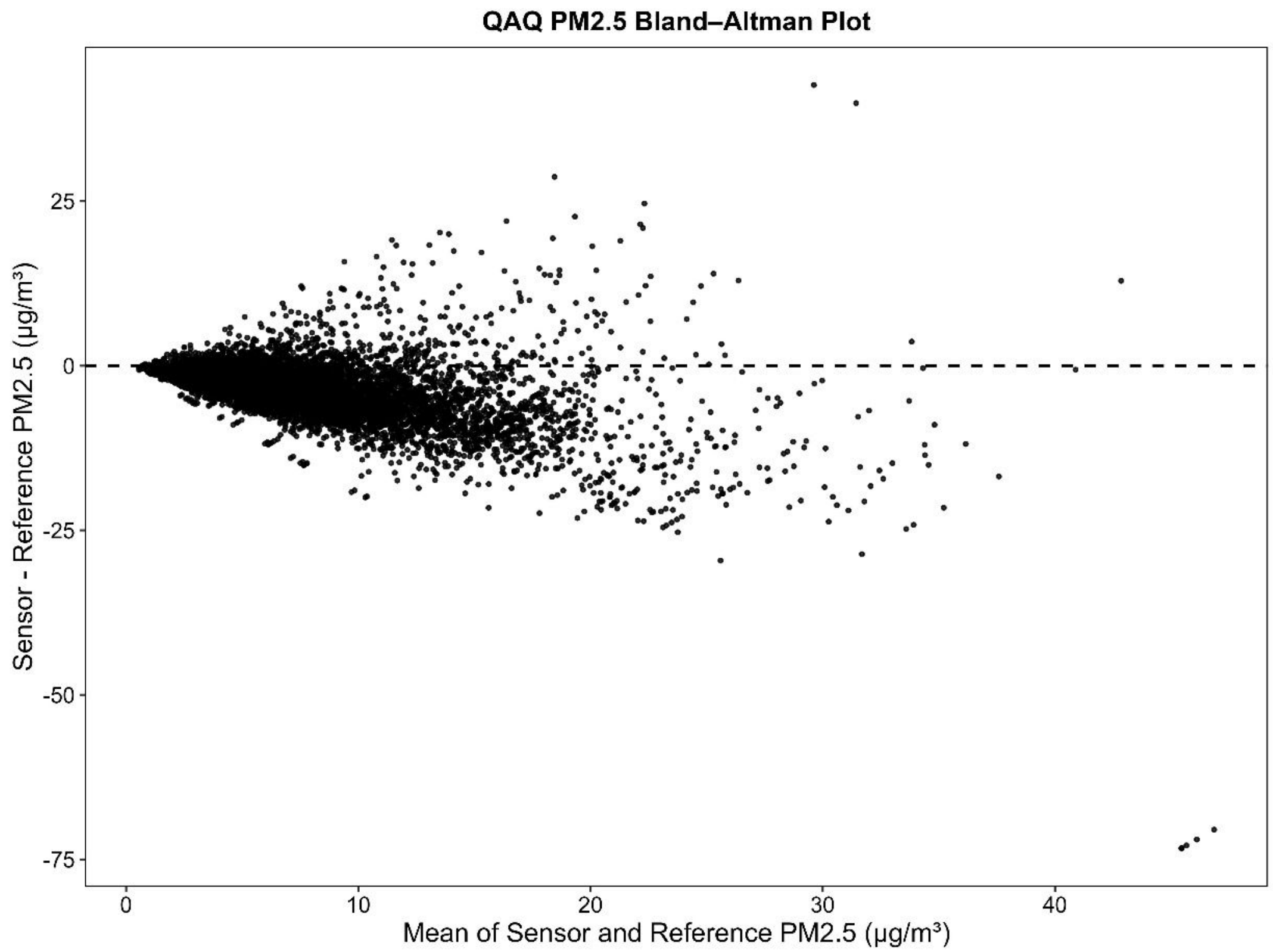

Figure S8:Bland–Altman plot comparing QAQ and reference $PM_{2.5}$ measurements.

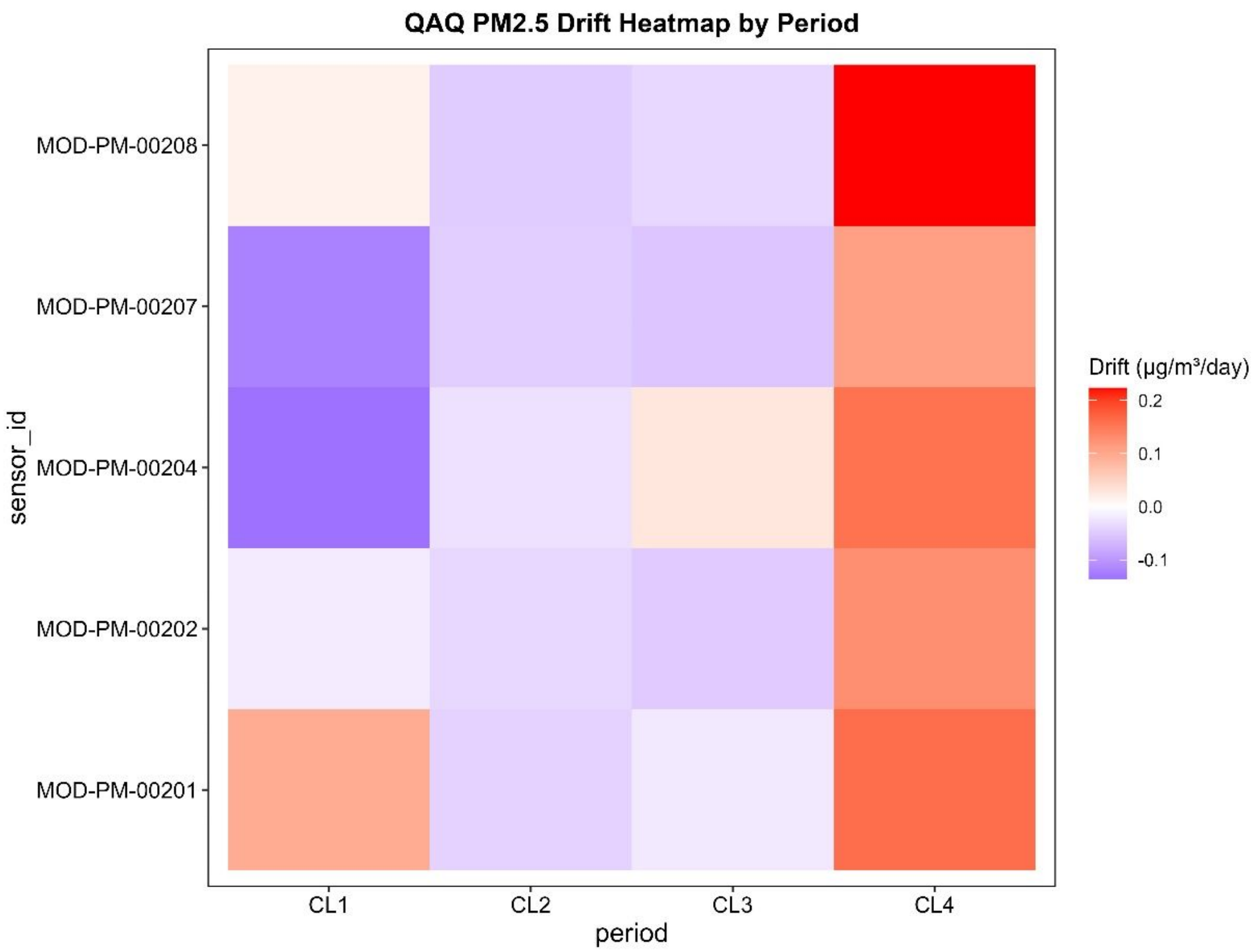

Figure S9: Drift (µg m^{-3}/day) for individual QAQ $PM_{2.5}$ by deployment period.

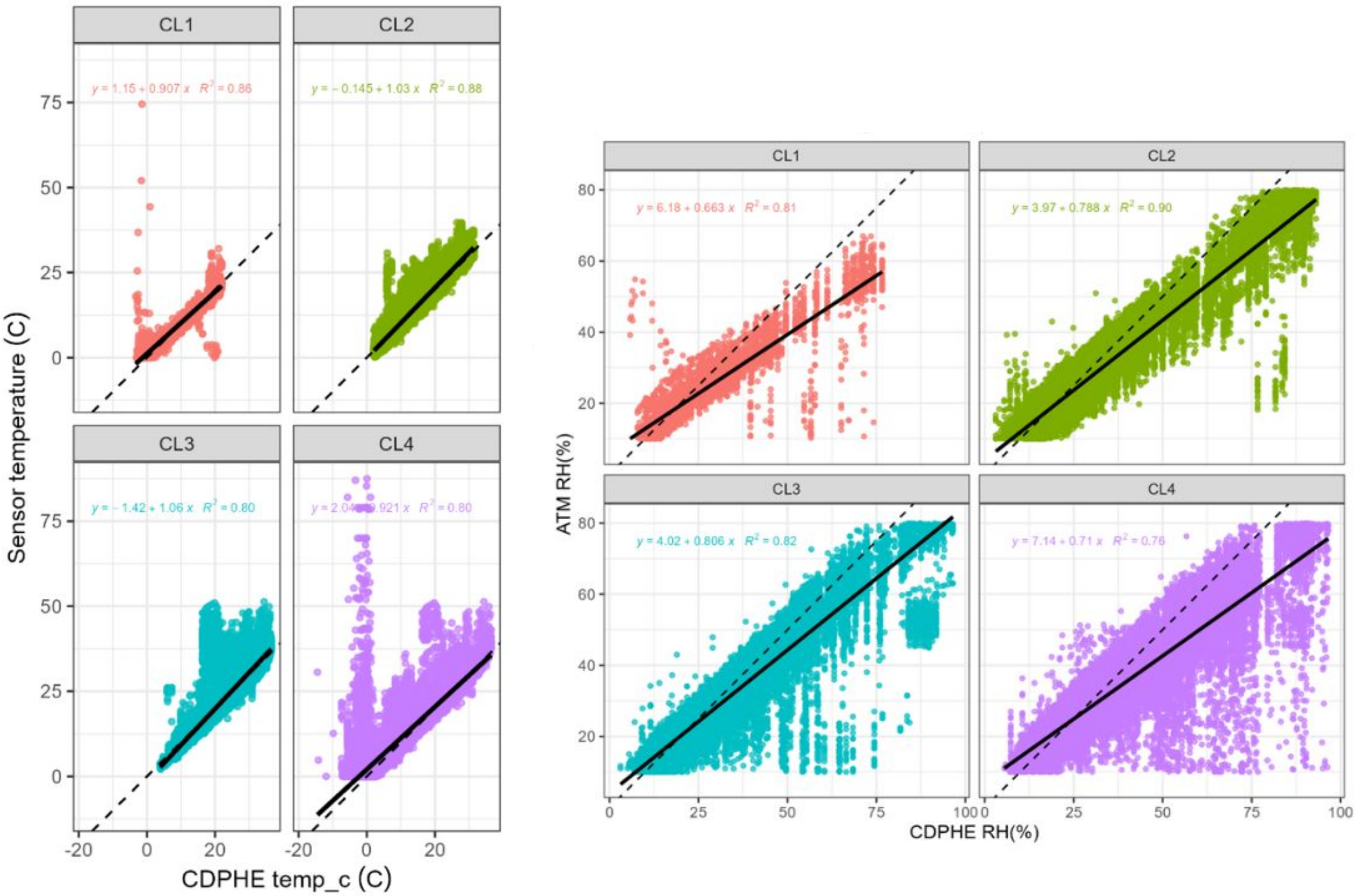

Figure S10: Comparison between the ATM and CDPHE GRIMM data of the 1-hour averaged (a) temperature (°C) and (b) RH (%) for each co-location. Elevated sensor temperatures observed in some deployments likely reflect enclosure heating during outdoor operation rather than ambient air temperature. Monitors exhibiting persistent abnormal temperature or RH behavior were identified through quality-control screening and excluded from subsequent analyses.

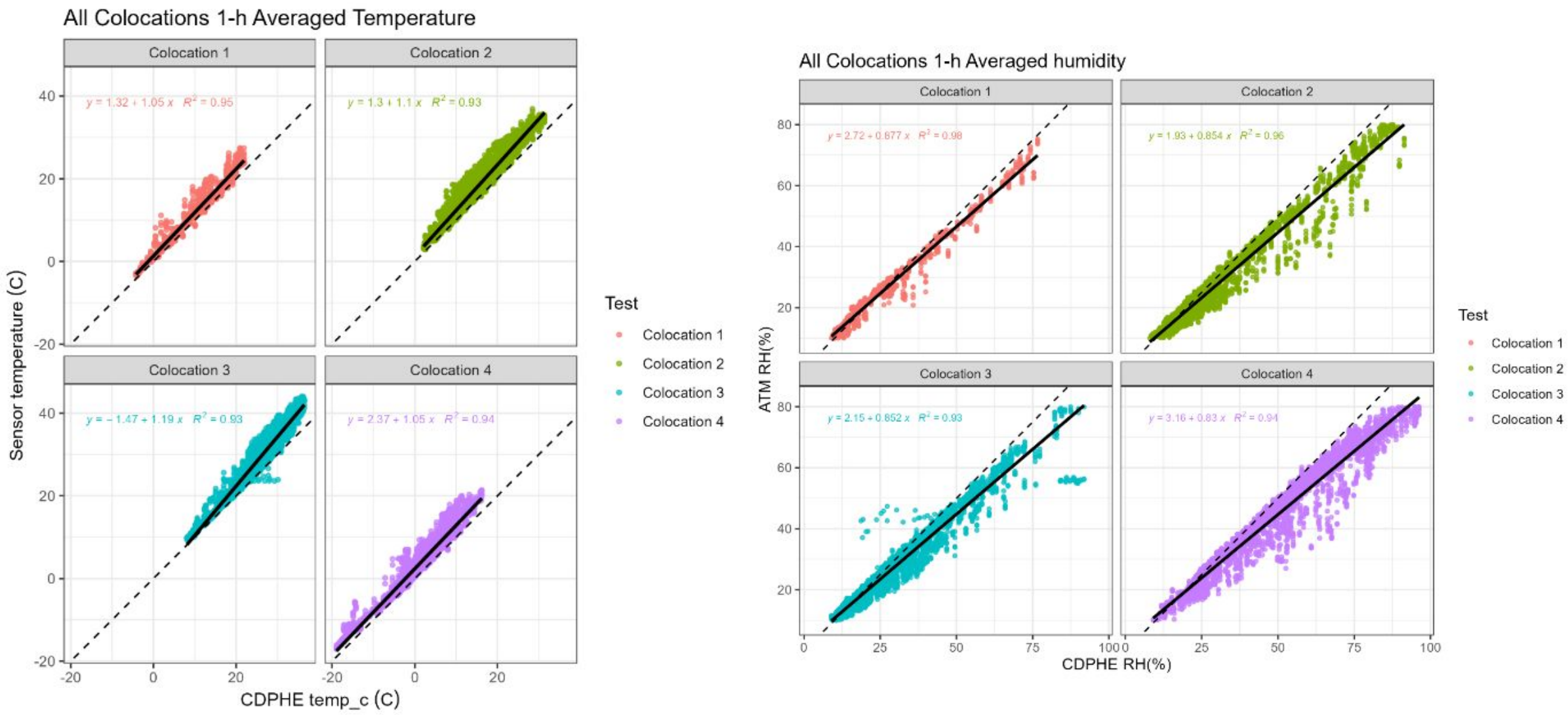

Figure S11: Comparison between the QAQ and CDPHE GRIMM data of the 1-hour averaged temperature (°C) and RH (%) for each co-location.

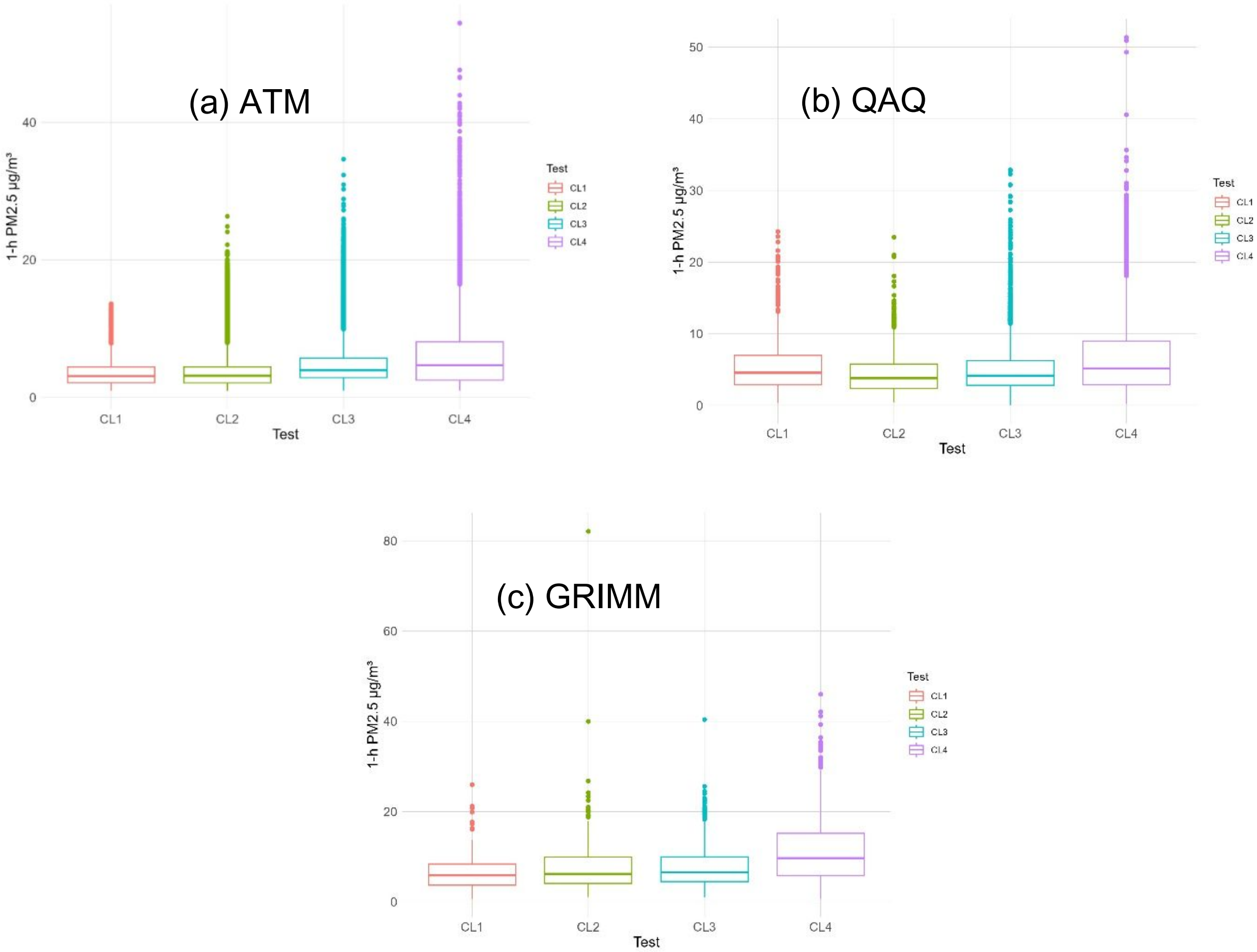

Figure S12: Box plots showing the distribution of cleaned hourly averaged $PM_{2.5}$ data for (a) ATM monitors, (b) QAQ monitors, and (c) GRIMM monitor (bottom).

(a)

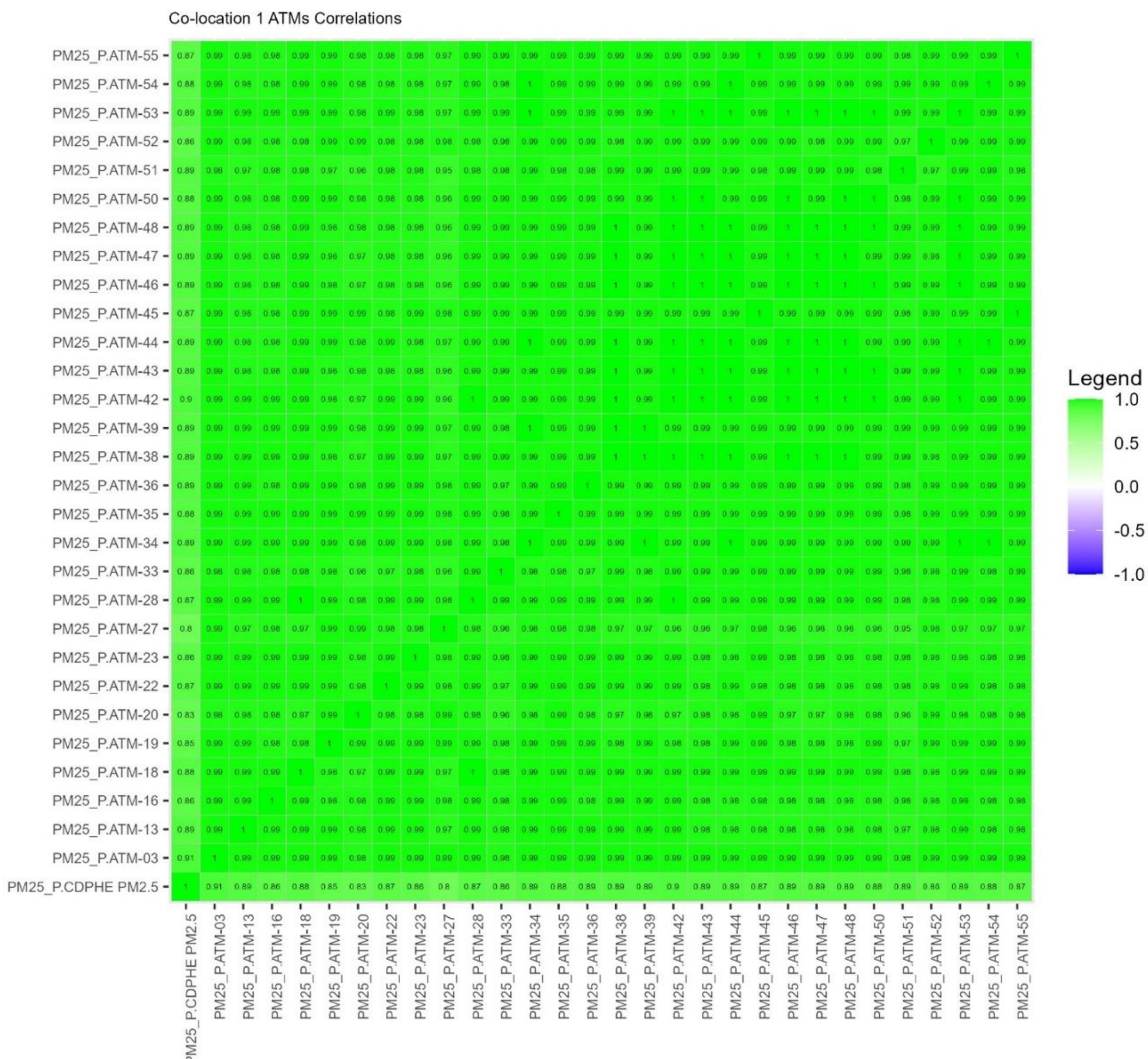
Co-location 1 ATMs Correlations
PM25_P.ATM-55
PM25_P.ATM-54
PM25_P.ATM-53
PM25_P.ATM-52
PM25_P.ATM-51
PM25_P.ATM-50
PM25_P.ATM-48
PM25_P.ATM-47
PM25_P.ATM-46
PM25_P.ATM-45
PM25_P.ATM-44
PM25_P.ATM-43
PM25_P.ATM-42
PM25_P.ATM-39
PM25_P.ATM-38
PM25_P.ATM-36
PM25_P.ATM-35
PM25_P.ATM-34
PM25_P.ATM-33
PM25_P.ATM-28
PM25_P.ATM-27
PM25_P.ATM-23
PM25_P.ATM-22
PM25_P.ATM-20
PM25_P.ATM-19
PM25_P.ATM-18
PM25_P.ATM-16
PM25_P.ATM-13
PM25_P.ATM-03
PM25_P.CDPHE PM2.5
Legend
1.0
0.5
0.0
-0.5
-1.0

(b)

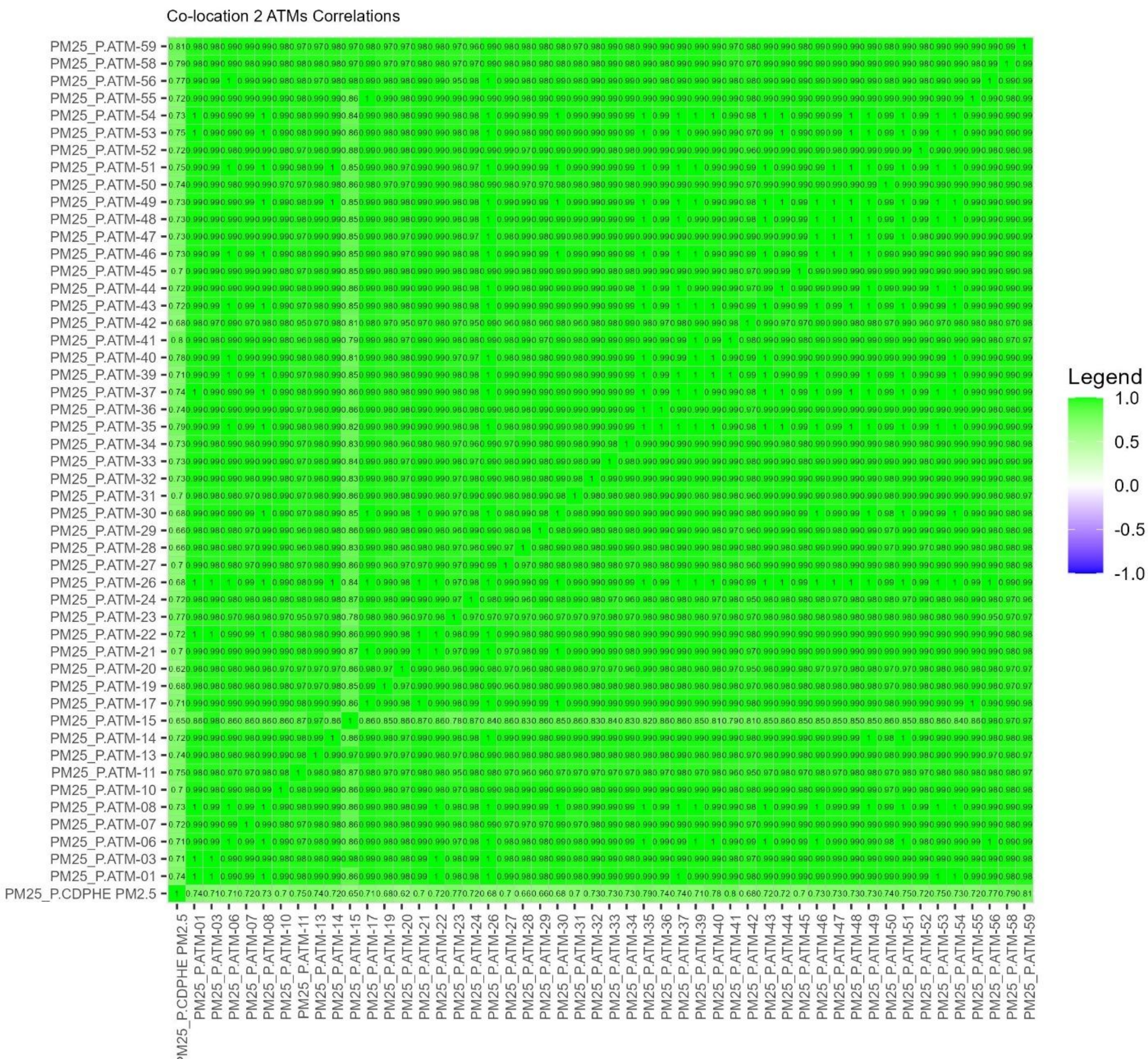
Co-location 2 ATMs Correlations
PM25_P.ATM-59
PM25_P.ATM-58
PM25_P.ATM-56
PM25_P.ATM-55
PM25_P.ATM-54
PM25_P.ATM-53
PM25_P.ATM-52
PM25_P.ATM-51
PM25_P.ATM-50
PM25_P.ATM-49
PM25_P.ATM-48
PM25_P.ATM-47
PM25_P.ATM-46
PM25_P.ATM-45
PM25_P.ATM-44
PM25_P.ATM-43
PM25_P.ATM-42
PM25_P.ATM-41
PM25_P.ATM-40
PM25_P.ATM-39
PM25_P.ATM-37
PM25_P.ATM-36
PM25_P.ATM-35
PM25_P.ATM-34
PM25_P.ATM-33
PM25_P.ATM-32
PM25_P.ATM-31
PM25_P.ATM-30
PM25_P.ATM-29
PM25_P.ATM-28
PM25_P.ATM-27
PM25_P.ATM-26
PM25_P.ATM-24
PM25_P.ATM-23
PM25_P.ATM-22
PM25_P.ATM-21
PM25_P.ATM-20
PM25_P.ATM-19
PM25_P.ATM-17
PM25_P.ATM-15
PM25_P.ATM-14
PM25_P.ATM-13
PM25_P.ATM-11
PM25_P.ATM-10
PM25_P.ATM-08
PM25_P.ATM-07
PM25_P.ATM-06
PM25_P.ATM-03
PM25_P.ATM-01
PM25_P.CDPHE PM2.5
Legend
1.0
0.5
0.0
-0.5
-1.0

(c)

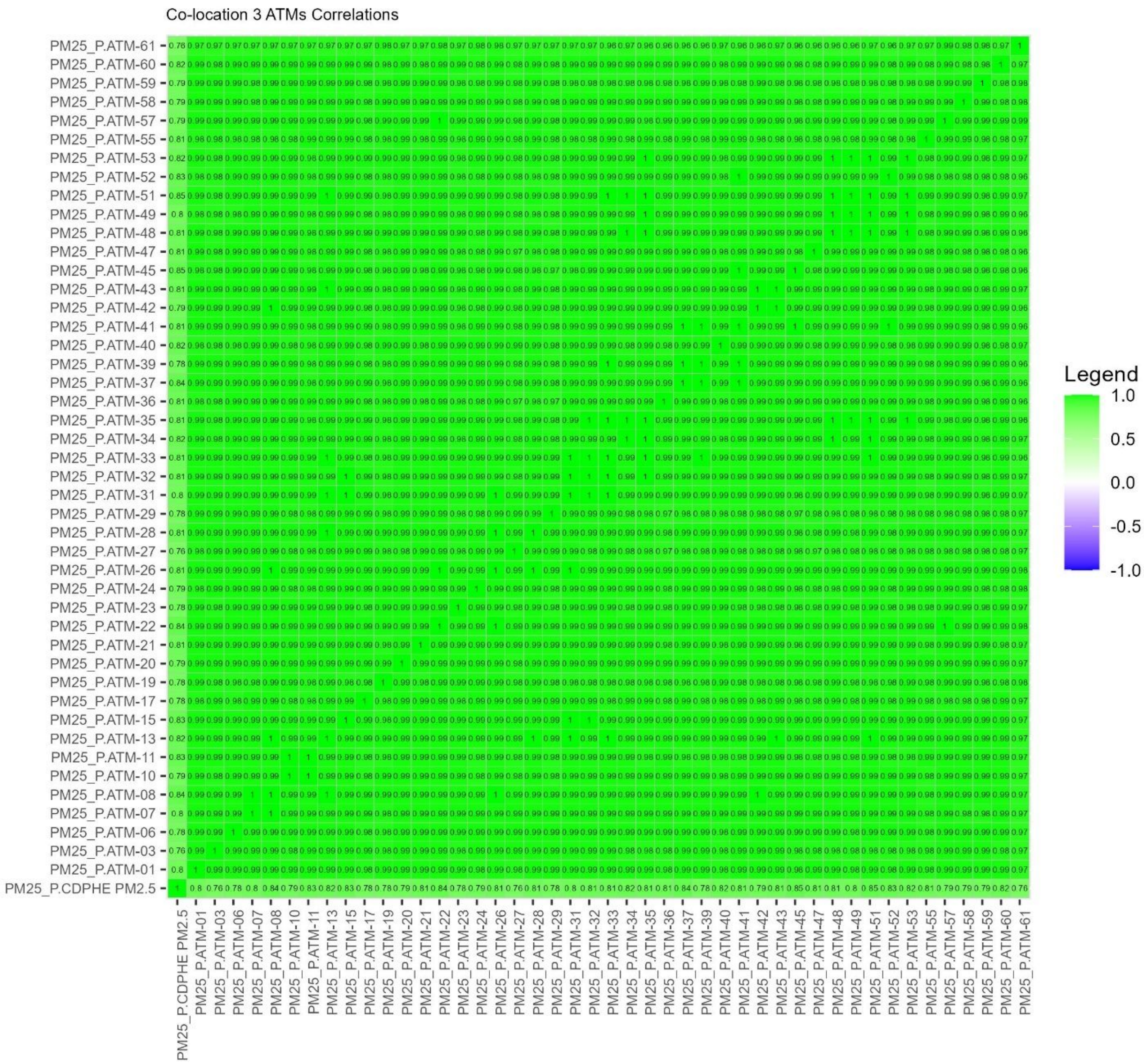
Co-location 3 ATMs Correlations
PM25_P.ATM-61
PM25_P.ATM-60
PM25_P.ATM-59
PM25_P.ATM-58
PM25_P.ATM-57
PM25_P.ATM-55
PM25_P.ATM-53
PM25_P.ATM-52
PM25_P.ATM-51
PM25_P.ATM-49
PM25_P.ATM-48
PM25_P.ATM-47
PM25_P.ATM-45
PM25_P.ATM-43
PM25_P.ATM-42
PM25_P.ATM-41
PM25_P.ATM-40
PM25_P.ATM-39
PM25_P.ATM-37
PM25_P.ATM-36
PM25_P.ATM-35
PM25_P.ATM-34
PM25_P.ATM-33
PM25_P.ATM-32
PM25_P.ATM-31
PM25_P.ATM-29
PM25_P.ATM-28
PM25_P.ATM-27
PM25_P.ATM-26
PM25_P.ATM-24
PM25_P.ATM-23
PM25_P.ATM-22
PM25_P.ATM-21
PM25_P.ATM-20
PM25_P.ATM-19
PM25_P.ATM-17
PM25_P.ATM-15
PM25_P.ATM-13
PM25_P.ATM-11
PM25_P.ATM-10
PM25_P.ATM-08
PM25_P.ATM-07
PM25_P.ATM-06
PM25_P.ATM-03
PM25_P.ATM-01
PM25_P.CDPHE PM2.5
Legend
1.0
0.5
0.0
-0.5
-1.0

(d)

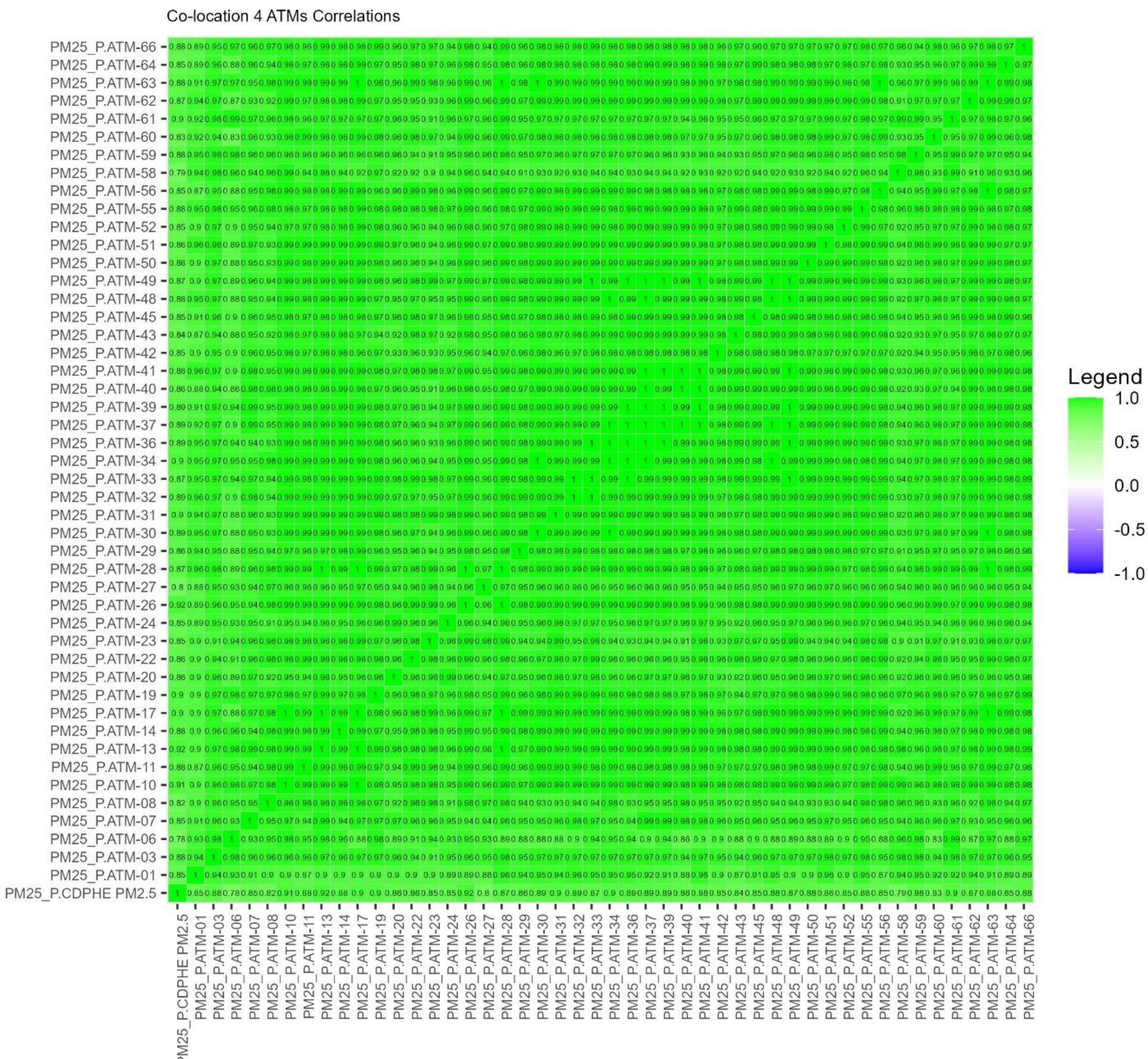

Figure S13: Correlation matrix after applying MLR model to ATM $PM_{2.5}$ data for each co-location after correction. a) CL1, b) CL2, c) CL3, and d) CL4.

(a)

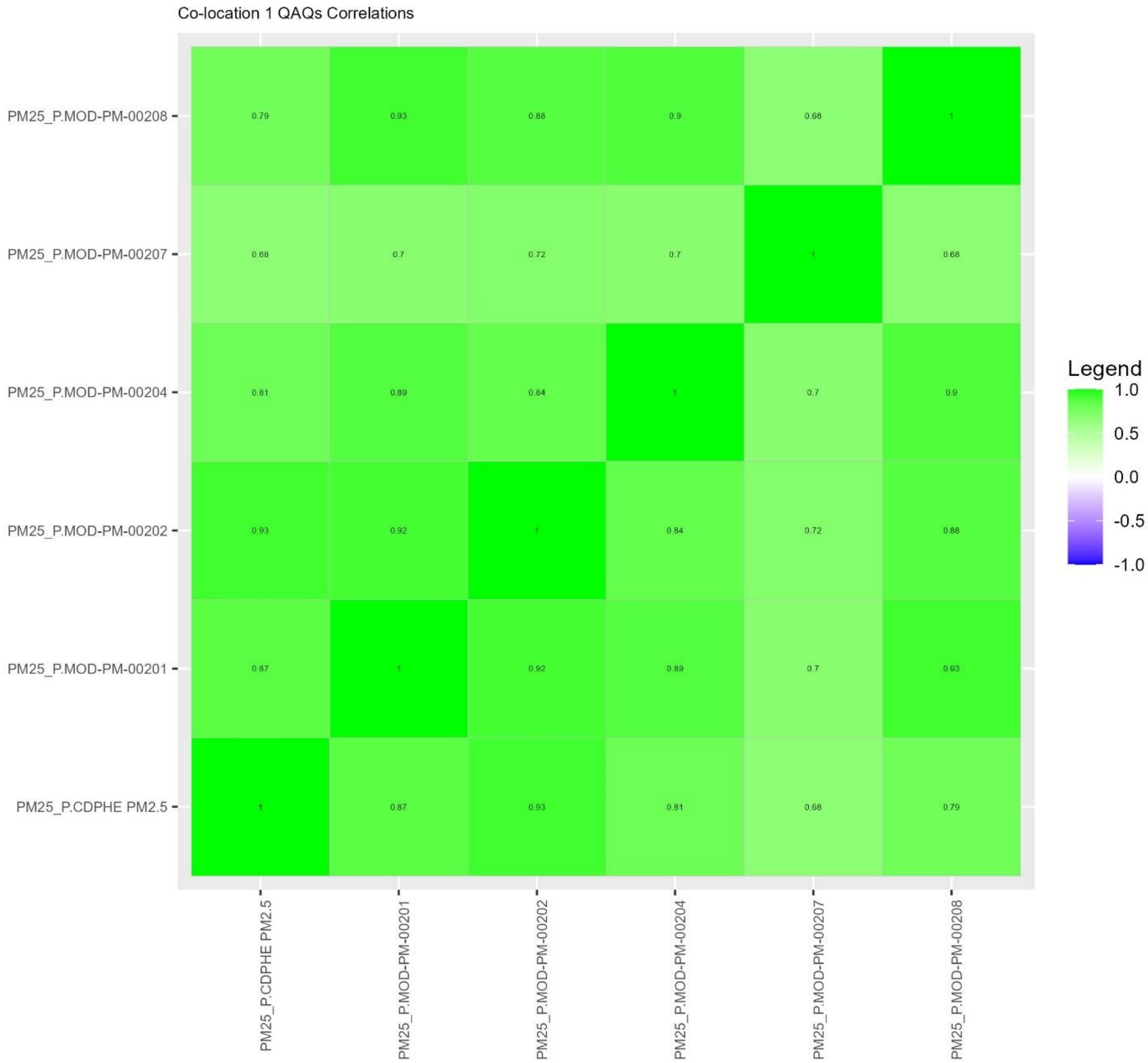

Co-location 1 QAQs Correlations
PM25_P.MOD-PM-00208
PM25_P.MOD-PM-00207
PM25_P.MOD-PM-00204
PM25_P.MOD-PM-00202
PM25_P.MOD-PM-00201
PM25_P.CDPHE PM2.5
0.79
0.93
0.88
0.9
0.68
1
0.68
0.7
0.72
0.7
1
0.68
0.81
0.89
0.84
1
0.7
0.9
0.93
0.92
1
0.84
0.72
0.88
0.87
1
0.92
0.89
0.7
0.93
1
0.87
0.93
0.81
0.68
0.79
PM25_P.CDPHE PM2.5
PM25_P.MOD-PM-00201
PM25_P.MOD-PM-00202
PM25_P.MOD-PM-00204
PM25_P.MOD-PM-00207
PM25_P.MOD-PM-00208
Legend
1.0
0.5
0.0
-0.5
-1.0

(b)

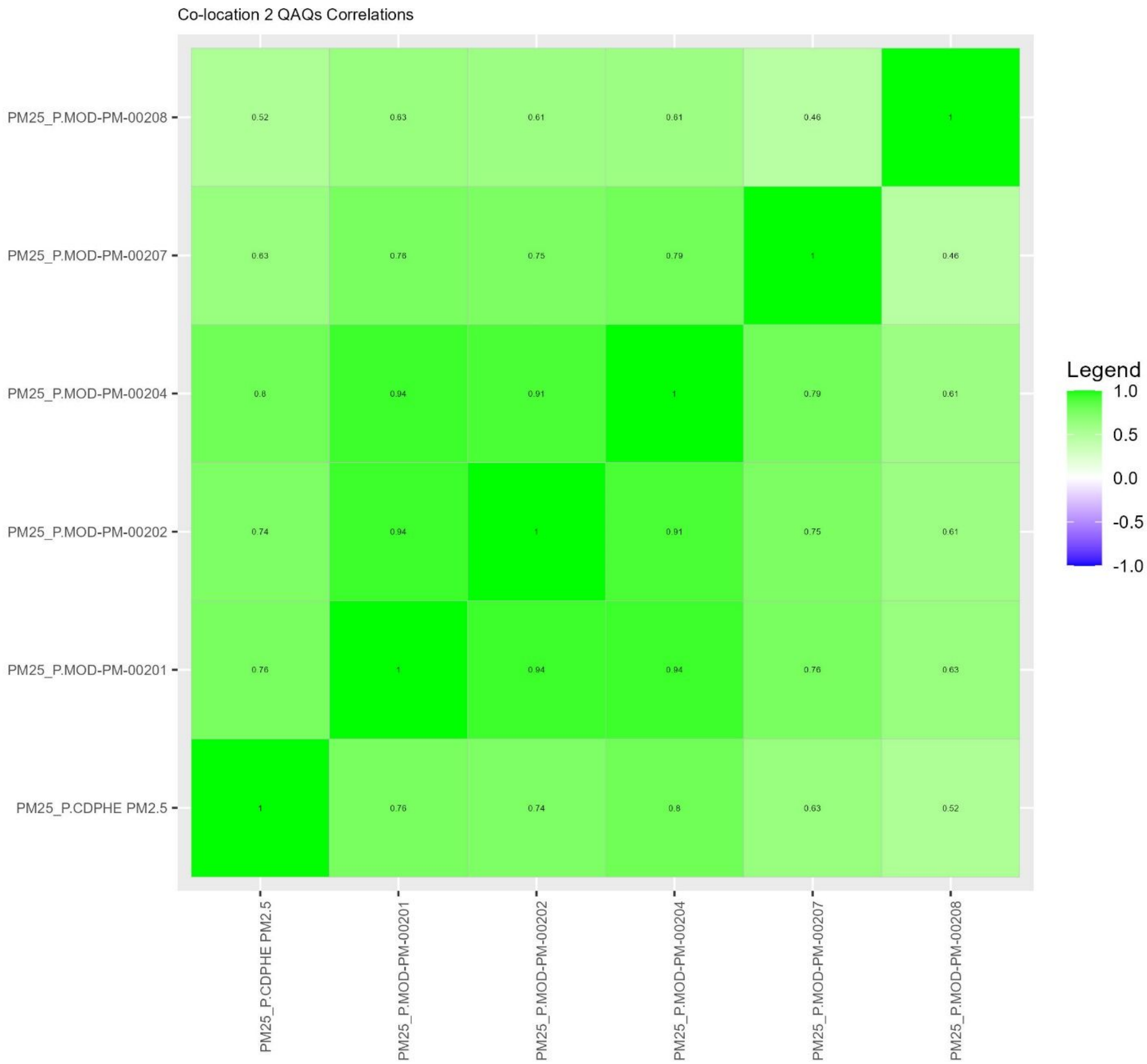
Co-location 2 QAQs Correlations
PM25_P.MOD-PM-00208
PM25_P.MOD-PM-00207
PM25_P.MOD-PM-00204
PM25_P.MOD-PM-00202
PM25_P.MOD-PM-00201
PM25_P.CDPHE PM2.5
0.52
0.63
0.61
0.61
0.46
1
0.63
0.78
0.75
0.79
1
0.46
0.8
0.94
0.91
1
0.79
0.61
0.74
0.94
1
0.91
0.75
0.61
0.76
1
0.94
0.94
0.78
0.63
1
0.76
0.74
0.8
0.63
0.52
PM25_P.CDPHE PM2.5
PM25_P.MOD-PM-00201
PM25_P.MOD-PM-00202
PM25_P.MOD-PM-00204
PM25_P.MOD-PM-00207
PM25_P.MOD-PM-00208
Legend
1.0
0.5
0.0
-0.5
-1.0

(c)

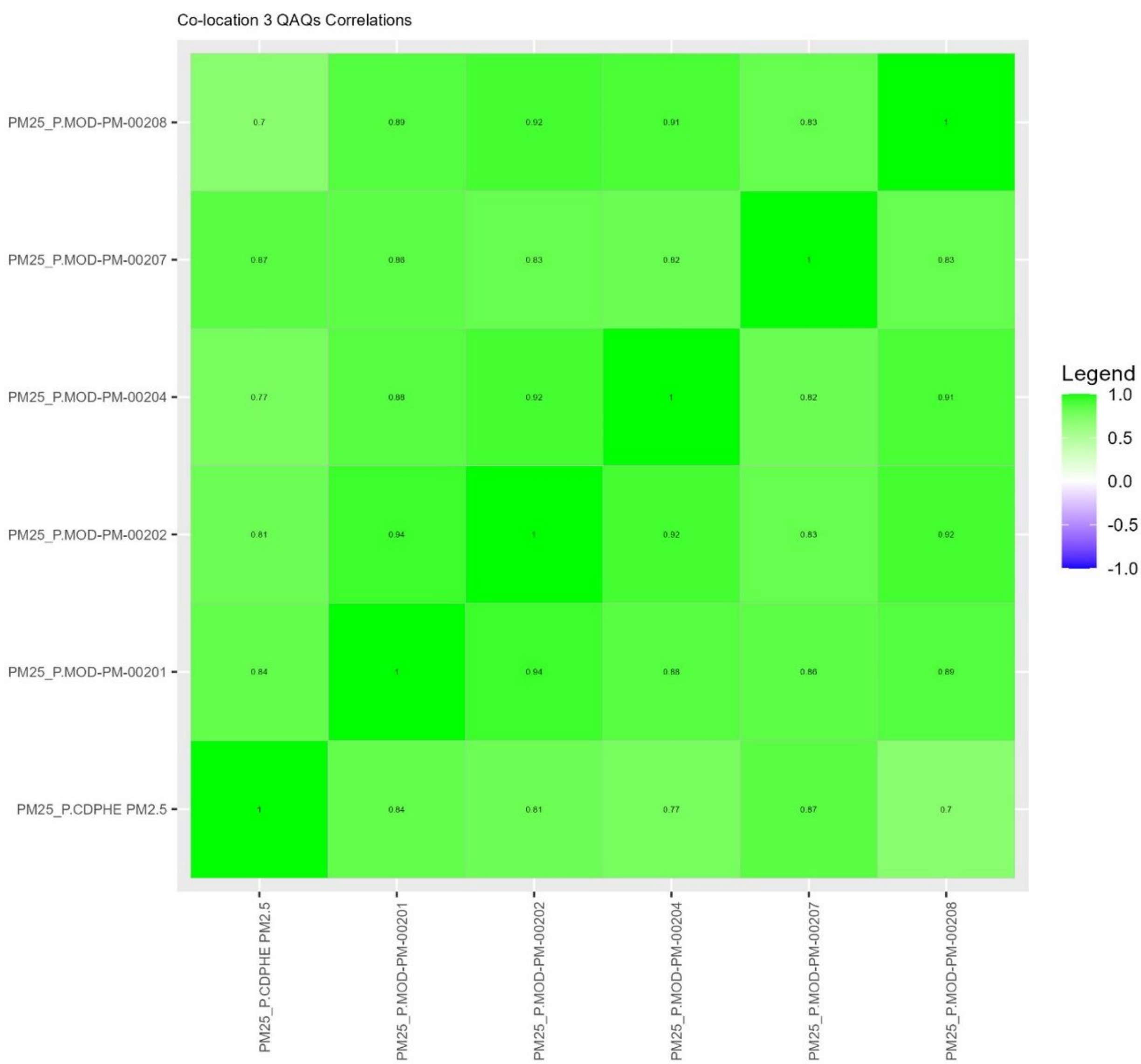

Co-location 3 QAQs Correlations
PM25_P.MOD-PM-00208
PM25_P.MOD-PM-00207
PM25_P.MOD-PM-00204
PM25_P.MOD-PM-00202
PM25_P.MOD-PM-00201
PM25_P.CDPHE PM2.5
0.7 0.89 0.92 0.91 0.83 1
0.87 0.86 0.83 0.82 1 0.83
0.77 0.88 0.92 1 0.82 0.91
0.81 0.94 1 0.92 0.83 0.92
0.84 1 0.94 0.88 0.86 0.89
1 0.84 0.81 0.77 0.87 0.7
PM25_P.CDPHE PM2.5
PM25_P.MOD-PM-00201
PM25_P.MOD-PM-00202
PM25_P.MOD-PM-00204
PM25_P.MOD-PM-00207
PM25_P.MOD-PM-00208
Legend
1.0
0.5
0.0
-0.5
-1.0

(d)

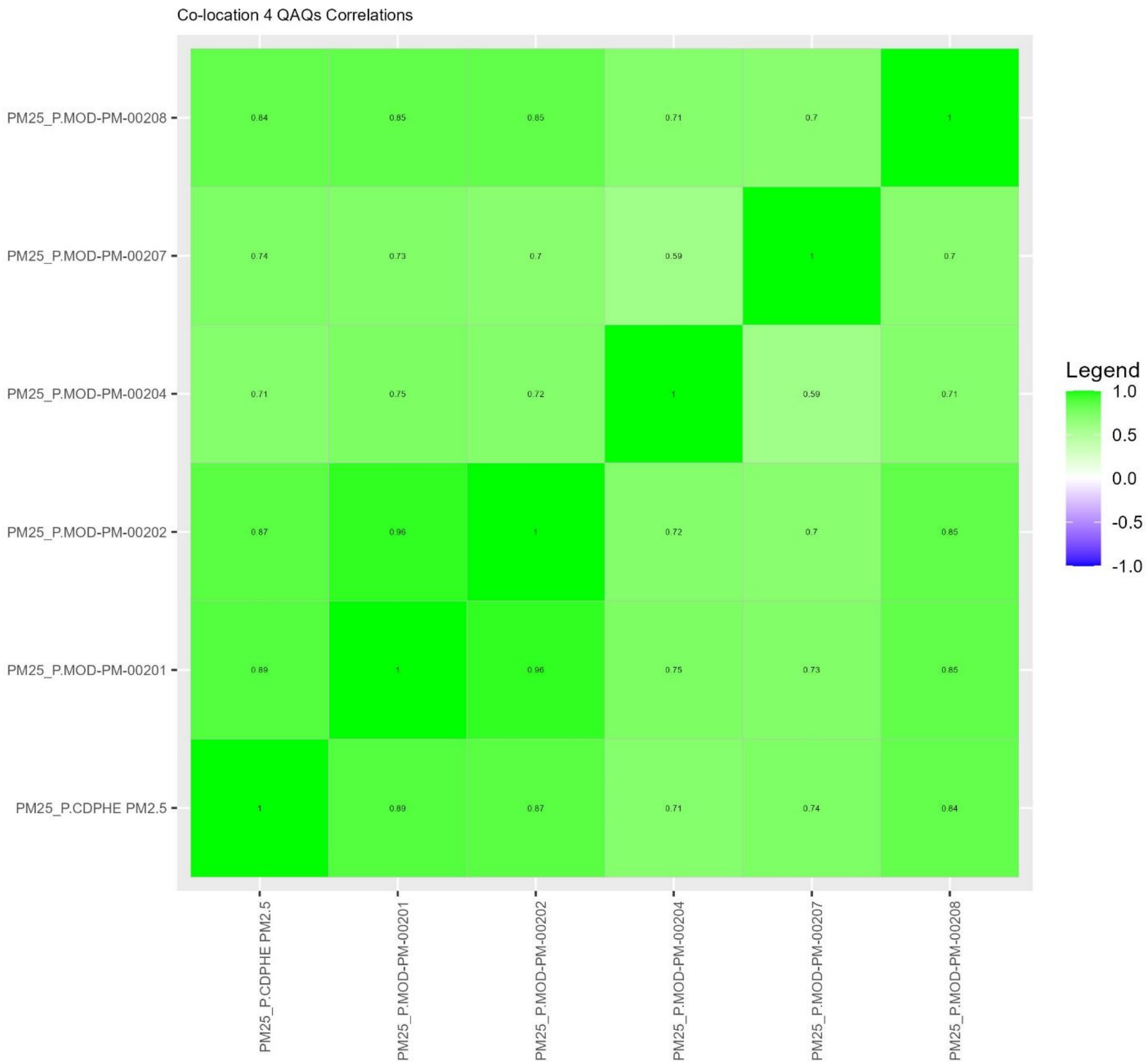

Figure S14: Correlation matrix after applying MLR model on QAQ $PM_{2.5}$ data for each co-location after correction. a) CL1, b) CL2, c) CL3, and d) CL4.

SI Equations

Equations S1-S5 were used to calculate standard performance metrics for the linear regression model comparing processed ATM and QAQ data to the GRIMM, following U.S. EPA guidance.

Coefficient of Determination $R^2 = \frac{\sum (y_i - x_i)^2}{\sum (y_i - \bar{y})^2}$ Eq. (S1)

Linear Regression Slope $m = \frac{\sum (y_i - \bar{y}) \times (x_i - \bar{x})}{\sum (x_i - \bar{x})}$ Eq. (S2)

Linear Regression y-Intercept b = $\bar{y} - m \times \bar{x}$ Eq. (S3)

Root Mean Square Error $RMSE_{multiple} = \sqrt{\frac{1}{N \times M} \sum_{j=1}^{M} \sum_{i=1}^{N} (y_{ij} - x_i)^2}$ Eq. (S4)

Normalized Root Mean Square Error NRMSE $= \frac{RMSE}{\bar{x}} \times 100$ Eq. (S5)

Where x denotes the GRIMM data, and y denotes the ATM or QAQ data.

SI Tables

Table S1: Percentage of RH Data <10% and >80% Excluded in Each Co-location after Removing the Defective Monitors

	ATMs				QAQs			
Co-location	RH<10 %	RH<20 %	RH>80 %	Median %	RH<10 %	RH<20 %	RH>80 %	Median %
CL1	8.04	31.9	0.12	24.2	2.56	24.0	0.0	27.2
CL2	10.2	32.2	1.73	26.1	10.6	33.3	3.28	26.0
CL3	7.13	24.2	2.39	33.1	5.11	26.1	3.69	31.3
CL4	17.8	32.0	4.22	33.1	0.72	8.64	2.35	49.3

Table S2: Summary of Atmotube Monitors' Temperature and RH Data at Each Co-location (CL)

ATM RH (%)	Min*	25th %ile	Median	Mean	75th %ile	Max
CL1	10.0	17.4	23.2	25.8	31.1	67.0
CL2	10.0	20.1	28.8	33.4	41.9	80.0
CL3	10.0	21.4	32.5	34.2	44.8	80.0
CL4	10.0	26.2	37.1	37.9	47.9	80.0

ATM Temperature (°C)	Min	25th %ile	Median	Mean	75th %ile	Max
CL1	0.00	4.97	9.58	10.3	15.3	30.7
CL2	0.167	9.33	13.9	14.7	19.8	39.7
CL3	5.00	18.2	22.7	23.3	28.5	51.3
CL4	0.00	1.83	4.00	5.25	7.80	36.0

*Min RH is 10% in all co-locations because data was cleaned for RH range 10-80%.

Table S3: Summary of CDPHE GRIMM Monitors' Temperature and RH Data at Each Co-location

RH (%)*	Min	25th %ile	Median	Mean	75th %ile	Max
CL1	5.80	15.9	23.9	28.7	37.6	76.6
CL2	3.10	17.6	27.9	34.3	44.9	93.1
CL3	5.50	21.8	35.0	37.8	48.7	96.5
CL4	7.30	38.4	55.1	54.4	70.5	96.3

Temperature (°C)	Min	25th %ile	Median	Mean	75th %ile	Max
CL1	-4.22	4.28	9.83	9.89	16.1	21.9
CL2	2.11	9.89	13.7	14.7	19.8	31.4
CL3	8.11	19.4	22.9	23.2	27.4	36.5
CL4	-18.9	-3.44	-0.056	-0.111	4.00	16.2

*We did not eliminate the RH values below 10.0% or above 80.0% from the CDPHE GRIMM data because their data is already quality controlled and the measurement technology is not based on LCSs.

Table S4: Summary of QuantAQ Monitors' Temperature and RH Data at Each Co-location

RH %	Min	25th %ile	Median	Mean	75th %ile	Max
CL1	10.0	19.0	25.5	29.5	36.8	75.2
CL2	10.0	19.6	27.8	32.8	41.5	80.0
CL3	10.0	20.8	31.4	32.8	42.6	80.0
CL4	10.1	33.8	48.6	47.6	62.4	80.0

Temperature (°C)	Min	25th %ile	Median	Mean	75th %ile	Max
CL1	3.54	5.19	10.9	11.3	17.2	27.5
CL2	2.86	11.7	16.6	17.4	23.2	36.9
CL3	9.47	21.3	25.4	26.3	31.4	44.0
CL4	17.1	1.72	1.88	2.29	6.20	21.4

Table S5: Summary of Atmotube Monitors' $PM_{2.5}$ Data at Each Co-location

$PM_{2.5}$ (μg m^{-3})	Min	25th %ile	Median	Mean	75th %ile	Max
CL1	1.00	2.07	3.05	3.42	4.25	13.6
CL2	0.953	2.08	3.07	3.88	4.47	47.4
CL3	1.00	2.93	4.05	5.15	6.03	34.6
CL4	1.00	3.04	5.55	7.83	10.2	58.9

Table S6: Summary of CDPHE GRIMM Monitors' $PM_{2.5}$ Data at Each Co-location

$PM_{2.5}$ (μg m^{-3})	Min	25th %ile	Median	Mean	75th %ile	Max
CL1	0.70	3.70	5.90	6.66	8.40	26.0
CL2	1.10	4.10	6.20	7.49	9.90	82.1
CL3	1.00	4.50	6.55	7.82	10.0	40.4
CL4	0.70	5.80	9.70	11.4	15.2	46.0

Table S7: Summary of QuantAQ Monitors' $PM_{2.5}$ Data at Each Co-location

$PM_{2.5}$ (μg m^{-3})	Min	25th %ile	Median	Mean	75th %ile	Max
CL1	0.383	2.93	4.58	5.39	6.99	24.2
CL2	0.428	2.42	3.77	4.40	5.68	23.5
CL3	0.041	2.75	4.11	5.07	6.20	32.9
CL4	0.229	2.92	5.17	6.59	8.95	51.4

Table S8: Mean Performance Metrics Comparing Individual ATM Data with Grimm Data at the I-25 Globeville Co-location Site (CL1)

ATM Sensor	R^2	Regression slope	y-intercept (μg m^{-3})	RMSE (μg m^{-3})	NRMSE (%)
ATM-03	0.720	0.410	0.800	4.86	73.0
ATM-13	0.750	0.400	0.630	4.39	66.0
ATM-16	0.700	0.410	0.760	4.22	63.4
ATM-18	0.760	0.430	0.590	4.19	63.0
ATM-19	0.740	0.390	0.620	4.49	67.4
ATM-20	0.720	0.350	0.800	4.53	68.0
ATM-22	0.760	0.420	0.580	4.26	63.9
ATM-23	0.750	0.410	0.580	4.34	65.1
ATM-27	0.720	0.330	0.700	4.72	70.8
ATM-28	0.750	0.420	0.670	4.18	62.8
ATM-33	0.660	0.380	0.620	4.59	68.9
ATM-34	0.700	0.430	0.570	4.23	63.5
ATM-35	0.740	0.350	0.660	4.75	71.4
ATM-36	0.730	0.410	0.600	4.33	64.9
ATM-38	0.730	0.420	0.560	4.29	64.4
ATM-39	0.740	0.430	0.560	4.27	64.1
ATM-42	0.730	0.450	0.590	4.07	61.1
ATM-43	0.690	0.440	0.590	4.21	63.1
ATM-44	0.750	0.400	0.580	4.45	66.8
ATM-45	0.690	0.380	0.560	4.63	69.4
ATM-46	0.710	0.420	0.580	4.28	64.2
ATM-47	0.680	0.450	0.590	4.09	61.4
ATM-48	0.730	0.440	0.580	4.14	62.1
ATM-50	0.740	0.450	0.560	4.08	61.3
ATM-51	0.740	0.430	0.620	4.22	63.3
ATM-52	0.710	0.360	0.620	4.73	71.0
ATM-53	0.750	0.420	0.630	4.27	64.1
ATM-54	0.730	0.410	0.570	4.36	65.4

ATM-55	0.720	0.350	0.580	4.77	71.7
Mean	0.730	0.410	0.620	4.38	65.7

Table S9: Mean Performance Metrics Comparing Individual ATM Data with Grimm Data at the I-25 Globeville Co-location Site (CL2)

ATM Sensor	R^2	Regression slope	y-intercept (μg m^{-3})	RMSE (μg m^{-3})	NRMSE (%)
ATM-01	0.430	0.370	1.32	5.30	70.7
ATM-03	0.430	0.340	1.26	5.65	75.4
ATM-06	0.400	0.360	1.30	5.42	72.3
ATM-07	0.460	0.350	1.30	5.43	72.5
ATM-08	0.440	0.370	1.33	5.30	70.7
ATM-10	0.430	0.370	1.19	5.41	72.2
ATM-11	0.520	0.390	1.12	5.17	69.1
ATM-13	0.530	0.380	1.03	5.34	71.3
ATM-14	0.470	0.360	1.16	5.45	72.8
ATM-15	0.390	0.310	1.44	5.68	75.8
ATM-17	0.450	0.360	1.07	5.53	73.8
ATM-19	0.410	0.340	1.21	5.61	74.8
ATM-20	0.340	0.270	1.55	6.02	80.4
ATM-21	0.430	0.360	1.22	5.46	72.9
ATM-22	0.460	0.370	1.17	5.37	71.6
ATM-23	0.650	0.270	1.32	6.04	80.7
ATM-24	0.470	0.300	1.04	5.96	79.6
ATM-26	0.400	0.370	1.44	5.87	78.3
ATM-27	0.430	0.310	1.23	5.78	77.2
ATM-28	0.380	0.370	1.38	5.48	73.2
ATM-29	0.370	0.290	1.34	5.99	80.0
ATM-30	0.400	0.350	1.40	5.51	73.6
ATM-31	0.440	0.320	1.14	5.74	76.6
ATM-32	0.470	0.290	1.09	5.93	79.1
ATM-33	0.470	0.350	1.13	5.53	73.8

ATM-34	0.490	0.380	1.15	5.33	71.1
ATM-35	0.490	0.490	0.270	4.65	62.1
ATM-36	0.490	0.360	1.15	5.48	73.2
ATM-37	0.470	0.360	1.21	5.34	71.3
ATM-39	0.440	0.350	1.29	5.46	72.9
ATM-40	0.470	0.500	0.310	4.69	62.6
ATM-41	0.600	0.310	1.22	5.70	76.1
ATM-42	0.420	0.500	1.32	4.84	64.6
ATM-43	0.460	0.360	1.30	5.43	72.6
ATM-44	0.490	0.340	1.15	5.54	74.0
ATM-45	0.420	0.310	1.19	5.83	77.8
ATM-46	0.460	0.370	1.17	5.48	73.1
ATM-47	0.470	0.400	1.13	5.22	69.8
ATM-48	0.490	0.370	1.15	5.36	71.6
ATM-49	0.480	0.360	1.17	5.35	71.5
ATM-50	0.500	0.380	1.27	5.34	71.3
ATM-51	0.490	0.400	1.16	5.24	69.9
ATM-52	0.480	0.310	1.13	5.79	77.3
ATM-53	0.520	0.370	1.14	5.39	71.9
ATM-54	0.470	0.350	1.33	5.52	73.7
ATM-55	0.450	0.320	1.19	5.69	76.0
ATM-56	0.510	0.640	-0.490	4.24	56.6
ATM-58	0.550	0.700	-0.320	3.70	49.4
ATM-59	0.520	0.660	-0.100	3.86	51.6
Mean	0.460	0.380	1.09	5.40	72.1

Table S10: Mean Performance Metrics Comparing Individual ATM Data with Grimm Data at the I-25 Globeville Co-location Site (CL3)

ATM Sensor	R^2	Regression slope	y-intercept (µg m^{-3})	RMSE (µg m^{-3})	NRMSE (%)
ATM-01	0.560	0.620	0.590	3.79	48.6
ATM-03	0.530	0.490	0.560	4.61	59.0
ATM-06	0.530	0.590	0.480	4.18	53.5

ATM-07	0.560	0.600	0.600	4.08	52.2
ATM-08	0.640	0.670	0.230	3.62	46.3
ATM-10	0.530	0.580	0.290	4.44	56.8
ATM-11	0.620	0.610	0.230	3.96	50.6
ATM-13	0.590	0.590	0.450	3.94	50.4
ATM-15	0.600	0.570	0.320	4.18	53.4
ATM-17	0.530	0.600	0.520	4.05	51.8
ATM-19	0.520	0.550	0.800	4.30	55.0
ATM-20	0.490	0.510	0.680	4.41	56.5
ATM-21	0.610	0.620	0.410	3.78	48.4
ATM-22	0.610	0.630	0.490	3.69	47.2
ATM-23	0.550	0.590	0.330	4.28	54.8
ATM-24	0.510	0.480	0.880	4.68	59.9
ATM-26	0.580	0.660	0.250	3.76	48.1
ATM-27	0.550	0.550	0.680	4.29	54.9
ATM-28	0.560	0.670	0.490	3.71	47.5
ATM-29	0.530	0.570	0.730	4.09	52.3
ATM-31	0.580	0.550	0.490	4.28	54.7
ATM-32	0.520	0.500	0.680	4.64	59.3
ATM-33	0.570	0.600	0.640	3.86	49.4
ATM-34	0.580	0.590	0.600	3.94	50.5
ATM-35	0.580	0.560	0.690	4.06	51.9
ATM-36	0.550	0.580	0.620	4.03	51.5
ATM-37	0.660	0.630	0.340	3.63	46.5
ATM-39	0.520	0.560	0.530	4.38	56.0
ATM-40	0.620	0.630	0.540	3.65	46.7
ATM-41	0.580	0.560	0.650	4.06	52.0
ATM-42	0.550	0.860	0.660	3.59	46.0
ATM-43	0.620	0.680	0.380	3.54	45.2
ATM-45	0.640	0.540	0.470	4.15	53.1
ATM-47	0.490	0.590	0.900	4.04	51.7
ATM-48	0.580	0.600	0.580	3.89	49.8
ATM-49	0.580	0.570	0.490	4.15	53.1
ATM-51	0.650	0.670	0.410	3.44	44.1

ATM-52	0.600	0.550	0.640	4.05	51.8
ATM-53	0.570	0.590	0.570	4.00	51.2
ATM-55	0.530	0.540	0.630	4.33	55.4
ATM-57	0.530	0.610	0.310	4.15	53.1
ATM-58	0.530	0.550	0.290	4.55	58.2
ATM-59	0.530	0.580	0.290	4.32	55.2
ATM-60	0.600	0.650	0.020	4.00	51.2
ATM-61	0.500	0.620	0.110	4.33	55.5
Mean	0.570	0.590	0.500	4.06	52.0

Table S11: Mean Performance Metrics Comparing Individual ATM Data with Grimm Data at the I-25 Globeville Co-location Site (CL4)

ATM Sensor	R^2	Regression slope	y-intercept (µg m^{-3})	RMSE (µg m^{-3})	NRMSE (%)
ATM-01	0.700	0.900	-1.44	5.26	46.2
ATM-03	0.690	0.830	-1.23	5.55	48.8
ATM-06	0.670	0.920	-1.501	5.51	48.4
ATM-07	0.720	0.840	-1.26	5.20	45.7
ATM-08	0.700	0.880	-1.15	5.22	45.9
ATM-10	0.690	0.960	-2.94	6.12	53.7
ATM-11	0.700	0.720	-0.490	5.19	45.6
ATM-13	0.680	0.650	-0.440	6.24	54.8
ATM-17	0.740	0.600	-0.380	5.59	49.0
ATM-19	0.690	0.880	-1.78	5.86	51.4
ATM-20	0.750	0.760	-1.08	5.37	47.2
ATM-22	0.700	0.850	-0.970	5.17	45.4
ATM-23	0.670	0.680	-0.180	5.51	48.4
ATM-24	0.720	0.670	-0.850	6.44	56.5
ATM-26	0.680	0.730	-0.720	5.67	49.8
ATM-27	0.550	0.570	0.060	7.18	63.0
ATM-28	0.640	0.710	-0.640	5.78	50.8
ATM-29	0.710	0.780	-1.91	6.12	53.8
ATM-30	0.720	0.730	-0.830	5.57	48.9

ATM-31	0.720	0.540	-0.240	6.84	60.0
ATM-32	0.730	0.720	-1.64	6.33	55.6
ATM-33	0.720	0.640	-0.710	6.24	54.8
ATM-34	0.760	0.650	-0.750	5.62	49.4
ATM-36	0.720	0.830	-2.31	5.96	52.3
ATM-37	0.750	0.590	-0.480	6.10	53.6
ATM-39	0.760	0.630	-0.700	5.83	51.2
ATM-40	0.710	0.740	-1.07	5.52	48.5
ATM-41	0.690	0.620	-0.500	6.25	54.9
ATM-42	0.670	1.170	-1.84	6.22	54.7
ATM-43	0.710	0.940	-1.60	4.71	41.3
ATM-45	0.710	0.500	-0.360	7.04	61.8
ATM-48	0.720	0.740	-0.940	5.56	48.8
ATM-49	0.720	0.710	-0.810	5.74	50.4
ATM-51	0.690	0.680	-0.510	5.77	50.6
ATM-52	0.680	0.560	-0.370	6.81	59.8
ATM-55	0.650	0.630	-0.360	6.11	53.7
ATM-58	0.650	0.820	-1.31	5.83	51.2
ATM-59	0.650	0.930	-1.53	5.74	50.4
ATM-60	0.690	0.740	-0.950	5.43	47.7
ATM-61	0.640	0.950	-2.18	6.26	54.9
ATM-62	0.610	0.630	-0.410	6.37	56.0
ATM-63	0.740	0.590	-0.350	6.07	53.3
ATM-64	0.590	0.600	-0.120	6.70	58.8
ATM-66	0.660	0.880	-1.50	5.73	50.3
Mean	0.690	0.740	-0.980	5.89	51.8

Table S12: Mean Performance Metrics Comparing Individual QAQ Data with Grimm Data at the I-25 Globeville Co-location Site (CL1)

QAQ Sensor	R^2	Regression slope	y-intercept (μg m^{-3})	RMSE (μg m^{-3})	NRMSE (%)
MODPM00201	0.760	0.750	0.220	2.52	37.8
MOD-PM-00202	0.820	0.690	0.360	2.56	38.5

MOD-PM-00204	0.670	0.680	0.950	2.70	40.6
MOD-PM-00207	0.440	0.690	1.27	3.53	53.1
MOD-PM-00208	0.610	0.640	0.520	3.25	48.8
Mean	0.660	0.690	0.660	2.91	43.8

Table S13: Mean Performance Metrics Comparing Individual QAQ Data with Grimm Data at the I-25 Globeville Co-location Site (CL2)

QAQ Sensor	R^2	Regression slope	y-intercept ($\mu g\ m^{-3}$)	RMSE ($\mu g\ m^{-3}$)	NRMSE (%)
MODPM00201	0.600	0.420	1.64	4.58	61.1
MOD-PM-00202	0.580	0.340	1.59	5.20	69.2
MOD-PM-00204	0.680	0.370	1.71	4.84	64.7
MOD-PM-00207	0.440	0.350	2.23	4.96	66.3
MOD-PM-00208	0.280	0.240	1.75	6.14	82.0
Mean	0.510	0.350	1.78	5.14	68.7

Table S14: Mean Performance Metrics Comparing Individual QAQ Data with Grimm Data at the I-25 Globeville Co-location Site (CL3)

QAQ Sensor	R^2	Regression slope	y-intercept ($\mu g\ m^{-3}$)	RMSE ($\mu g\ m^{-3}$)	NRMSE (%)
MODPM00201	0.650	0.540	0.060	4.47	57.1
MOD-PM-00202	0.600	0.650	0.190	3.79	48.5
MOD-PM-00204	0.550	0.860	0.280	3.63	46.5
MOD-PM-00207	0.720	0.570	0.780	3.57	45.6
MOD-PM-00208	0.440	0.400	0.690	5.23	66.9
Mean	0.590	0.610	0.400	4.14	52.9

Table S15: Mean Performance Metrics Comparing Individual QAQ Data with Grimm Data at the I-25 Globeville Co-location Site (CL4)

QAQ Sensor	R^2	Regression slope	y-intercept ($\mu g\ m^{-3}$)	RMSE ($\mu g\ m^{-3}$)	NRMSE (%)
MODPM00201	0.820	0.480	0.010	7.22	63.4
MOD-PM-00202	0.770	0.550	-0.120	6.58	57.7
MOD-PM-00204	0.440	0.550	1.93	6.61	58.1
MOD-PM-00207	0.500	0.530	2.23	6.27	55.0
MOD-PM-00208	0.620	0.370	0.800	8.23	72.2
Mean	0.630	0.500	0.970	6.98	61.3

Table S16: Model Performance Metrics for One QAQ Monitor (Number 207) from CL2 Using Four Different Multilinear Regression Models

Model ID	R^2	Standard Error
A*	0.502	2.94
B**	0.495	2.96
C***	0.501	2.94
D****	0.487	2.97

*Model A: uses $PM\_Corrected = \beta 0 + \beta 1 \times (GRIMM\ PM_{2.5}) + \beta 2 \times RH + \beta 3 \times T_{dew}$

**Model B: uses $PM\_Corrected = \beta 0 + \beta 1 \times (GRIMM\ PM_{2.5}) + \beta 2 \times RH$

***Model C: uses $PM\_Corrected = \beta 0 + \beta 1 \times (GRIMM\ PM_{2.5}) + \beta 2 \times T_{dew}$

****Model D: uses $PM\_Corrected = \beta 0 + \beta 1 \times (GRIMM\ PM_{2.5})$

Table S17: Model Performance Metrics for One ATM Monitor (ATM-42) from CL2 Using Four Different Multilinear Regression Models

Model ID	R^2	Standard Error
A*	0.651	2.47
B**	0.584	2.68
C***	0.416	4.32
D****	0.372	4.40

*Model A: uses PM_Corrected = β0 + β1× (GRIMM $PM_{2.5}$) + β2 × RH + β3 × T_{dew}

**Model B: uses PM_Corrected = β0 + β1× (GRIMM $PM_{2.5}$) + β2 × RH

***Model C: uses PM_Corrected = β0 + β1× (GRIMM $PM_{2.5}$) + β2 × T_{dew}

****Model D: uses PM_Corrected = β0 + β1× (GRIMM $PM_{2.5}$)

Table S18: Parameters Used in the MLR Correction Model Based on Individual QAQ Coefficients for CL2

QAQ	Intercept	$PM_{2.5}$ coefficient	RH coefficient	T_{dew} coefficient
MOD-PM-00201	-0.193	1.421	0.032	0.016
MOD-PM-00202	-0.167	1.675	0.026	0.023
MOD-PM-00204	-0.761	1.808	0.009	0.013
MOD-PM-00207	3.911	1.100	-0.037	0.239
MOD-PM-00208	7.287	0.962	-0.067	0.453
Mean	2.015	1.393	-0.007	0.149

Table S19: Model Parameters for Each QAQ After Correction Based on Individual QAQ Coefficients for CL2

QAQ Sensor	R^2	Regression slope	y-intercept (μg m^{-3})	RMSE (μg $m^{-3})^3$	NRMSE (%)
MOD-PM-00201	0.586	0.588	3.16	3.49	46.6
MOD-PM-00202	0.550	0.553	3.43	3.64	48.6
MOD-PM-00204	0.646	0.646	2.70	3.21	42.9
MOD-PM-00207	0.404	0.405	4.55	4.18	55.8
MOD-PM-00208	0.313	0.314	5.26	4.47	59.6
Mean	0.500	0.501	3.82	3.80	50.7

Table S20: Model Results for Correcting QAQ $PM_{2.5}$ Data Based on Individual vs. Mean Values in CL2

CL2	R^2	Standard Error
Individual	0.500	2.64
Mean	0.480	2.75

Table S21: Parameters Used in the MLR Correction Model Based on Individual ATM Coefficients for CL2

ATM	Intercept	PM2.5 coefficient	RH coefficient	Tdew coefficient
ATM-01	6.28	1.28	-0.104	0.177
ATM-03	6.74	1.37	-0.108	0.222
ATM-06	6.91	1.2	-0.107	0.209
ATM-07	5.77	1.4	-0.0979	0.158
ATM-08	6.09	1.3	-0.1	0.173
ATM-10	6.28	1.24	-0.0956	0.171
ATM-11	5.24	1.4	-0.0908	0.139
ATM-13	4.96	1.49	-0.0883	0.105
ATM-14	5.63	1.41	-0.0945	0.133
ATM-15	6.02	1.24	-0.0744	0.213
ATM-17	6.18	1.33	-0.0958	0.163
ATM-19	6.74	1.27	-0.102	0.203
ATM-20	6.14	1.4	-0.0928	0.141
ATM-21	6.25	1.3	-0.0983	0.174
ATM-22	6.17	1.32	-0.0999	0.18
ATM-23	-0.389	2.65	-0.0528	-0.137
ATM-24	5.6	1.67	-0.094	0.146
ATM-26	6.66	1.21	-0.119	0.0996
ATM-27	5.76	1.49	-0.1	0.0978
ATM-28	6.75	1.12	-0.103	0.148
ATM-29	6.09	1.4	-0.0937	0.117
ATM-30	6.38	1.27	-0.107	0.141
ATM-31	5.86	1.47	-0.0936	0.154
ATM-32	5.59	1.71	-0.0971	0.156
ATM-33	5.59	1.47	-0.0966	0.129
ATM-34	5.54	1.41	-0.0972	0.132
ATM-35	7.23	1.09	-0.0928	0.287
ATM-36	5.39	1.5	-0.0979	0.123
ATM-37	5.65	1.44	-0.1	0.149

ATM-39	6.24	1.35	-0.105	0.173
ATM-40	7.52	1.02	-0.0938	0.295
ATM-41	2.47	2.14	-0.0833	0.0195
ATM-42	6.38	1.01	-0.103	0.169
ATM-43	5.83	1.38	-0.101	0.143
ATM-44	5.41	1.55	-0.097	0.134
ATM-45	6.29	1.48	-0.101	0.179
ATM-46	6.1	1.39	-0.106	0.157
ATM-47	6.28	1.28	-0.105	0.178
ATM-48	5.7	1.43	-0.101	0.149
ATM-49	5.64	1.44	-0.0996	0.14
ATM-50	6.24	1.39	-0.11	0.197
ATM-51	5.78	1.34	-0.0999	0.149
ATM-52	4.93	1.69	-0.0884	0.107
ATM-53	5.02	1.5	-0.0913	0.103
ATM-54	5.44	1.44	-0.0985	0.102
ATM-55	5.73	1.52	-0.0976	0.146
ATM-56	7.4	0.805	-0.0727	0.315
ATM-58	7.01	0.783	-0.0716	0.328
ATM-59	7.11	0.805	-0.0759	0.318
Mean	5.870	1.379	-0.096	0.159

Table S22: Model Parameters for Each ATMs after Correction Based on Individual ATM Coefficients for CL2

ATM Sensor	R2	Regression slope	y-intercept (μg m−3)	RMSE (μg m−3)3	NRMSE (%)
ATM-01	0.541	0.54	3.16	3.59	48
ATM-03	0.507	0.49	3.42	3.99	53.3
ATM-06	0.505	0.511	3.46	3.68	49.1
ATM-07	0.52	0.515	3.55	3.75	50
ATM-08	0.533	0.543	3.36	3.58	47.8
ATM-10	0.492	0.506	3.36	3.77	50.3

ATM-11	0.562	0.572	2.98	3.57	47.7
ATM-13	0.551	0.56	3.03	3.67	49
ATM-14	0.512	0.532	3.39	3.69	49.3
ATM-15	0.428	0.435	4.25	4.05	54.1
ATM-17	0.502	0.51	3.65	3.73	49.8
ATM-19	0.461	0.43	3.99	4.13	55.2
ATM-20	0.387	0.38	4.51	4.42	59
ATM-21	0.486	0.473	3.73	3.97	53
ATM-22	0.523	0.528	3.42	3.7	49.4
ATM-23	0.664	0.677	2.24	3.31	44.2
ATM-24	0.53	0.518	3.39	3.73	49.8
ATM-26	0.472	0.48	3.62	4.47	59.7
ATM-27	0.504	0.473	3.51	3.95	52.7
ATM-28	0.443	0.451	3.85	4.04	53.9
ATM-29	0.438	0.436	4.08	4	53.4
ATM-30	0.471	0.478	3.64	3.92	52.3
ATM-31	0.492	0.481	3.62	3.81	50.9
ATM-32	0.523	0.553	4.04	3.76	50.2
ATM-33	0.527	0.528	3.6	3.71	49.5
ATM-34	0.531	0.542	3.72	3.75	50.1
ATM-35	0.638	0.691	1.92	2.43	32.4
ATM-36	0.549	0.548	3.39	3.73	49.8
ATM-37	0.54	0.555	3.13	3.63	48.4
ATM-39	0.508	0.525	3.28	3.74	49.9
ATM-40	0.633	0.685	2.07	2.36	31.5
ATM-41	0.672	0.619	2.72	3.25	43.4
ATM-42	0.471	0.502	3.68	3.89	52
ATM-43	0.513	0.527	3.35	3.81	50.9
ATM-44	0.525	0.54	3.22	3.69	49.3
ATM-45	0.486	0.496	3.48	3.84	51.2
ATM-46	0.535	0.55	3.28	3.7	49.4
ATM-47	0.532	0.543	3.19	3.67	49
ATM-48	0.54	0.558	3.17	3.64	48.6
ATM-49	0.533	0.55	3.29	3.64	48.6

ATM-50	0.551	0.552	3.21	3.65	48.7
ATM-51	0.558	0.567	3.16	3.62	48.3
ATM-52	0.527	0.543	3.42	3.68	49.1
ATM-53	0.559	0.577	3.16	3.58	47.9
ATM-54	0.53	0.539	3.45	3.75	50.1
ATM-55	0.518	0.529	3.36	3.71	49.5
ATM-56	0.632	0.625	2.59	2.4	32.1
ATM-58	0.678	0.672	2.26	2.21	29.5
ATM-59	0.698	0.67	2.13	2.14	28.6
Mean	0.531	0.537	3.32	3.62	48.4

Table S23: Model Results for Correcting ATM $PM_{2.5}$ Data Based on Individual vs. Mean Values in CL2

CL2	R^2	Standard Error
Individual	0.531	2.64
Mean	0.520	2.70
